# Borophene: Crucial Challenges and the Way Forward

**DOI:** 10.1002/advs.202517082

**Published:** 2026-03-15

**Authors:** Zhixuan Li, Jeyaraman Sankar, Prashant Kumar, Ajayan Vinu

**Affiliations:** ^1^ Global Innovative Centre For Advanced Nanomaterials School of Engineering College of Engineering Science and Environment The University of Newcastle Callaghan New South Wales Australia; ^2^ Department of Chemistry Indian Institute of Science Education and Research (IISER) Bhopal Bhopal Madhya Pradesh India; ^3^ Department of Applied Sciences School of Advanced Engineering University of Petroleum and Energy Studies, Energy Acres Dehradun Uttarakhand India

**Keywords:** borophene, electronic properties, energy storage, quantum 2D materials, synthesis techniques

## Abstract

Borophene, a monolayer of boron atoms, exhibits anisotropy in crystallographic structure and hence anisotropic physical and chemical behavior. It has emerged as a disruptive two‐dimensional (2D) quantum material due to its exceptional electronic mobility, Young's moduli with half‐auxetic nature of crystals and due to its chemical reactivity. Borophene exhibits polymorphism with structure dependent electronic nature. While metallic β_12_ & X_3_ phases and semiconducting α phase have already been explored, several new crystallographic phases are presently being explored. However, its cumbersome synthesis protocols remain a major challenge, hindering large‐scale production of defect free borophene. Despite its advantages, borophene faces limitations such as oxidation sensitivity, phase instability, and the absence of a band gap. Strategies like defect engineering, surface functionalization, and 2D‐2D hybridization offer potential solutions for band gap tuning and carrier injection. Device integration issues include imperfect interfaces, work function mismatch, interfacial charging etc. If these challenges can be overcome, borophene can then find applications in spintronics, photonics, flexible batteries, and quantum computing, paving the way for commercialization. This review highlights the current state‐of‐the‐art of borophene research and outlines strategies for overcoming existing barriers, positioning borophene as a functional atom‐thin layer in next‐generation devices and sensors.

## Graphical abstract

This review explores the challenges and progress in borophene research, focusing on synthesis strategies, structural properties, and potential applications. It highlights key experimental breakthroughs, discusses theoretical insights into borophene's unique features, and addresses pathways for overcoming stability and scalability issues. Future perspectives are also presented to advance borophene's integration into emerging technologies.

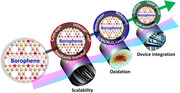

## 1 Introduction

**FIGURE 1:**
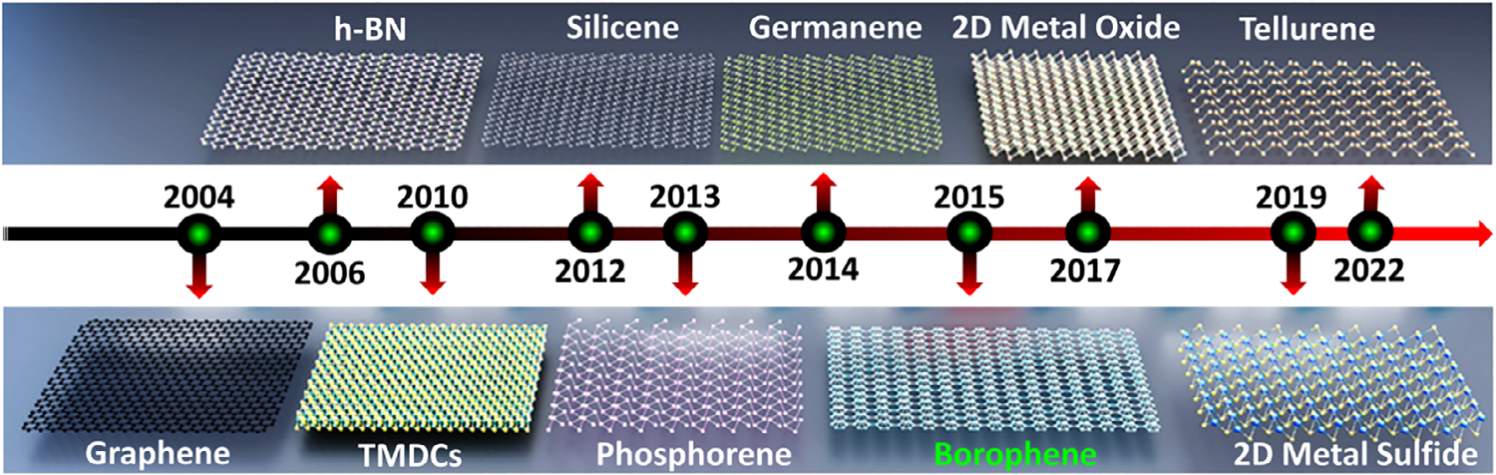
Timeline of key discoveries in two‐dimensional (2D) materials.

Quantum confinement in low‐dimensional materials gives them unique physical and chemical properties, enabling their use in niche and frontier applications. Superior electronic mobility, exceptional thermal conductivity, high Young's modulus, and enhanced chemical reactivity make these quantum materials invaluable for next‐generation technologies [1, 2]. Among quantum materials, 2D materials, which exhibit flat atomic sheets with electrons confined to two‐dimensional movement, have revolutionized material science and prompted recent advancements in technological applications [3, 4, 5, 6, 7, 8, 9, 10, 11, 12, 13, 14]. The evolution of 2D materials over the past two decades, as summarized in Figure 1, highlights key milestones from the discovery of graphene in 2004 to the emergence of materials like borophene and tellurene in recent years, showcasing the diversity of 2D materials.

The discovery of graphene, a sp^2^‐hybridized hexagonal atomic lattice of carbon, by Geim and Novoselov in 2004 [15], was a path breaking moment in materials science and engineering. Graphene's superlative attributes, including extremely high electronic mobility, record Young's modulus, and high thermal conductivity, positions it as the “miracle material” of the 21^st^ century [16, 17, 18, 19, 20]. Its success inspired the development of family of materials termed as “Xenes” which are mono‐elemental atom‐thin 2D sheets, including phosphorene [21, 22], silicene [23], germanene [24], stannene [25], arsenene, antimonene, bismuthene [26], and borophene [27, 28, 29, 30, 31, 32]. In parallel, metallic 2D materials have emerged, such as gallenene (p‐block) [33], beryllene (s‐block) [34], and d‐block metallenes like goldene [35], molybdenene [36] and several others [37, 38]. Beyond Xenes and metallenes, various 2D compounds like transition metal dichalcogenides (TMDCs), [39] MXenes [40], 2D transition metal oxides (2DTMOs) [41, 42, 43, 44, 45, 46, 47, 48, 49], boron nitride (BN) [50], carbon nitride [51, 52, 53, 54], gallium nitride (GaN) [55], 2D metal phosphides [56], 2D metal borides (MBenes) [57], etc., have expanded the landscape of 2D materials. Besides the discovery of several classes of 2D materials systems, novel approaches of 3D straining [58], substitutional doping [59, 60, 61], and orbital hybridization in 2D‐2D heterostacks [62, 63] have been developed which have resulted in designer materials with desirable physical and chemical properties eventually advancing their frontier applications in various domain.

The discovery of anisotropic crystals of borophene has been a significant milestone in the context of advancements of 2D materials due to its distinct physical and chemical properties. Quantum mechanical behavior of borophene positions it in special category and a lot more needs to be done to accomplish full‐fledged quantum devices [64]. Nanoarchitectonics protocols for borophene such as doping [65] and hybridization [66] have been discovered. Further, fabrication strategies for functional devices and sensors have been developed [67]. The single‐crystal nature of borophene at monolayer thickness minimizes electronic scattering, enabling ballistic carrier transport with superior mobility [68]. Distinctly, borophene's Dirac nature and anisotropic metallic behavior enhance electronic mobility, particularly along the ridgelines of its β_12_ and χ_3_ phases [69]. These attributes position borophene as a strong candidate for low power electronic chips, useful for ultra‐fast sensing of toxic gaseous molecules, and for portable health diagnostics via bio sensing such as for diabetic and cancer.

Beyond its electronic advantages, borophene demonstrates exceptional optical transparency, high thermal conductivity, and superior elastic moduli, making it versatile for various applications [70]. Its unique ability to exhibit multi‐center electron bonding (2e‐nc, where n = 2–7), topological and superconducting properties [71], metallic conductivity [72], and chemical/electrochemical reactivity [73] further strengthens its potential for applications in niche devices and sensors. These properties make borophene one of the most promising 2D materials for applications such as superconducting devices [74], thermoelectric generators [75], quantum computers [64], bioimaging and biosensing [76], hybrid ion supercapacitors [77], high‐capacity and high‐rate batteries [78], and fuel cells for green hydrogen production [79].

Despite its extraordinary properties, borophene faces several challenges that limit its practical deployment. Apart from synthetic challenges to produce high quality defect‐free borophene in a scalable manner, surface oxidation is a critical issue that hinders its stability and applicability across devices [80]. Additionally, the lack of a band gap in borophene restricts its ON/OFF ratio in electronic chips, posing challenges for electronic and optoelectronic applications [81]. Its limited electron availability (number of electron per atom) in the lattice constrains its catalytic performance [82]. While out‐of‐plane protrusions and periodic vacancies results in relatively stronger inter‐layer coupling, which renders exfoliation challenging, in‐plane chemical bonds in lattice poses challenges in nanoarchitectonics such as defect generation or doping. However, if these challenges are overcome, borophene can then be ready to be utilized in its dream applications. Furthermore, due to inefficient compatibility (i.e., work function mismatch) with transition metal electrodes limits its integration into devices and sensors [83]. Moreover, lack of protocols for fabricating borophene based hetero‐layers free from air bubble/voids is yet another issue. Although significant theoretical progress has been made in understanding borophene's lattice structures and nanoarchitectonics, developing practical devices and sensors remains mostly unrealized.

The present article illustrates how the discovery of borophene is highly desirable and very timely for targeted electronic, electrochemical, and catalytic applications where other existing nanomaterials, as well as 2D materials, lacked the qualities needed for superior performances, especially under high power operation, under extreme thermal conditions, under cycling, etc. The present review digs deeper into the issues concerning borophene. Various alternative synthetic approaches to replace the existing ones have been dealt with which possibly will help in large scale production of high quality borophene. The issues regarding its chemical phase purity (mainly surface oxidation), scalability, and reproducibility, have been detailed in brief. The review glances over the borophene's limitations vis‐à‐vis other competitive materials systems and outlines strategies to overcome them. To amend them to suit various prospective applications, approaches to nanoarchitectonics of borophene are suggested. Further, device‐related strategies have been dealt with, including surface passivation, work function engineering, interface engineering, and band alignment. A holistic summary of the rise of borophene and related materials as a new class of materials system has been presented and a futuristic outlook is provided to advance the journey forward.

## 2 Discovery of Borophene

**FIGURE 2:**
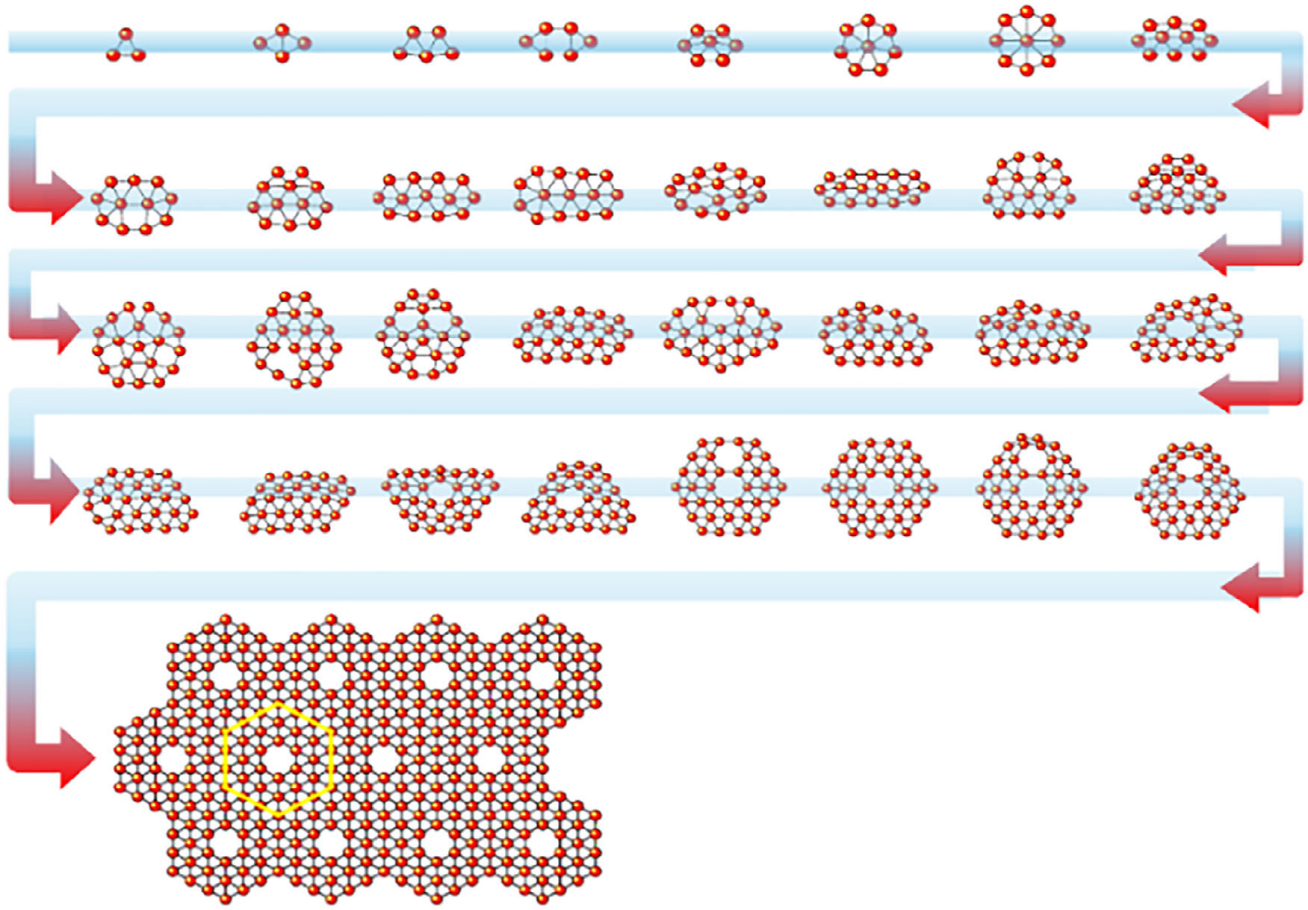
Boron clusters with a gradually increased number of atoms, eventually forming borophene. Reproduced with permission. [85] Copyright 2017, Macmillan Publishers Limited.

Boron atoms, with their electron‐deficient nature, were long thought incapable of forming stable 2D sheets. Theoretical frameworks predicting stable phases of boron sheets existed, but challenges, including the toxicity and high cost of diborane precursors, hindered such structures' experimental realization. The fullerene‐like icosahedral structure of diborane posed additional complexity, as assembling it into a planar 2D lattice seemed not feasible. In the 1990s, physicist I. Boustani carried out computational simulations to investigate the stability of boron clusters across various sizes and geometries [84]. His groundbreaking studies revealed that boron sheets may have preferably quasi‐planar configurations over the expected icosahedral arrangements. These discoveries are graphically summarized in Figure 2, illustrating the minimum‐energy structures of size‐selected boron clusters [85]. The figure highlights boron clusters' dominant planar or quasi‐planar nature across various sizes, where hexagonal vacancies and triangular motifs are key structural elements.

**FIGURE 3:**
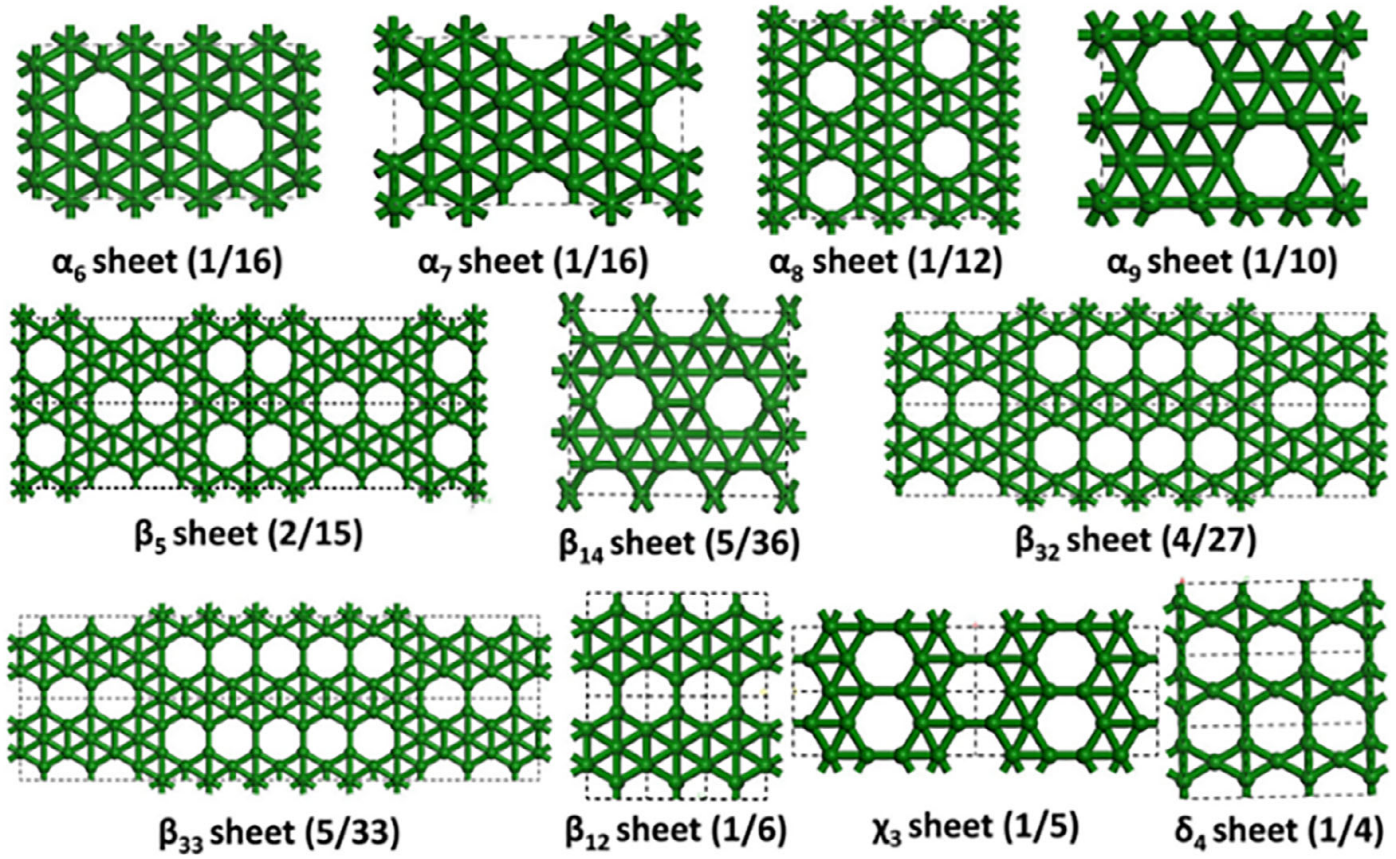
Crystallographic phases of borophene.Reproduced with permission [88]. Copyright 2021 Springer Nature Switzerland AG.

Further theoretical advancements came in 2007 when Lau et al. used density functional theory (DFT) to calculate the free energy of various boron layer structures [86]. Building on this, Piaza et al. predicted planar borophene structures, emphasizing that vacancies were essential for achieving stable borophene lattices [87]. Figure 3 provides a detailed depiction of various borophene phases, highlighting the intricate role of vacancies in stabilizing these structures [88]. These studies underscored the role of vacancies in stabilizing borophene's unique crystallographic phases, although the relationship between vacancy concentration and phase stability required further exploration.

The borophene with higher electron deficiency can have improved stability on the metal surface as it would help to transfer the electrons from the metal support to the borophene sheet. Unlike graphene and hexagonal boron nitride (BN), which exhibit a single stable 2D phase (sp^2^ hybridized hexagonal lattices), boron lacks the requisite electrons for such bonding and relies on unique 2e‐nc bonding mechanisms. This enables borophene to exist in multiple crystallographic phases, each with distinct properties. However, understanding the mechanisms of crystal growth of borophene is essential for unlocking its potential. Since then, several theoretical studies have been devoted to understanding the structural elucidation of various borophene phases, as illustrated in Figure 3 [88].

Borophene differs from other Xenes because it exhibits multiple stable polymorphic structures with intrinsic vacancy patterns, arising from boron's electron‐deficient, multi‐center bonding, whereas most Xenes have a single dominant lattice with simple two‐center covalent bonds. Its structure can be planar or highly buckled depending on the phase, shows strong in‐plane anisotropy, and is intrinsically metallic, while other Xenes typically have uniform lattices, limited buckling, fewer structural variations, and are semi‐metallic or semiconducting.

**FIGURE 4:**
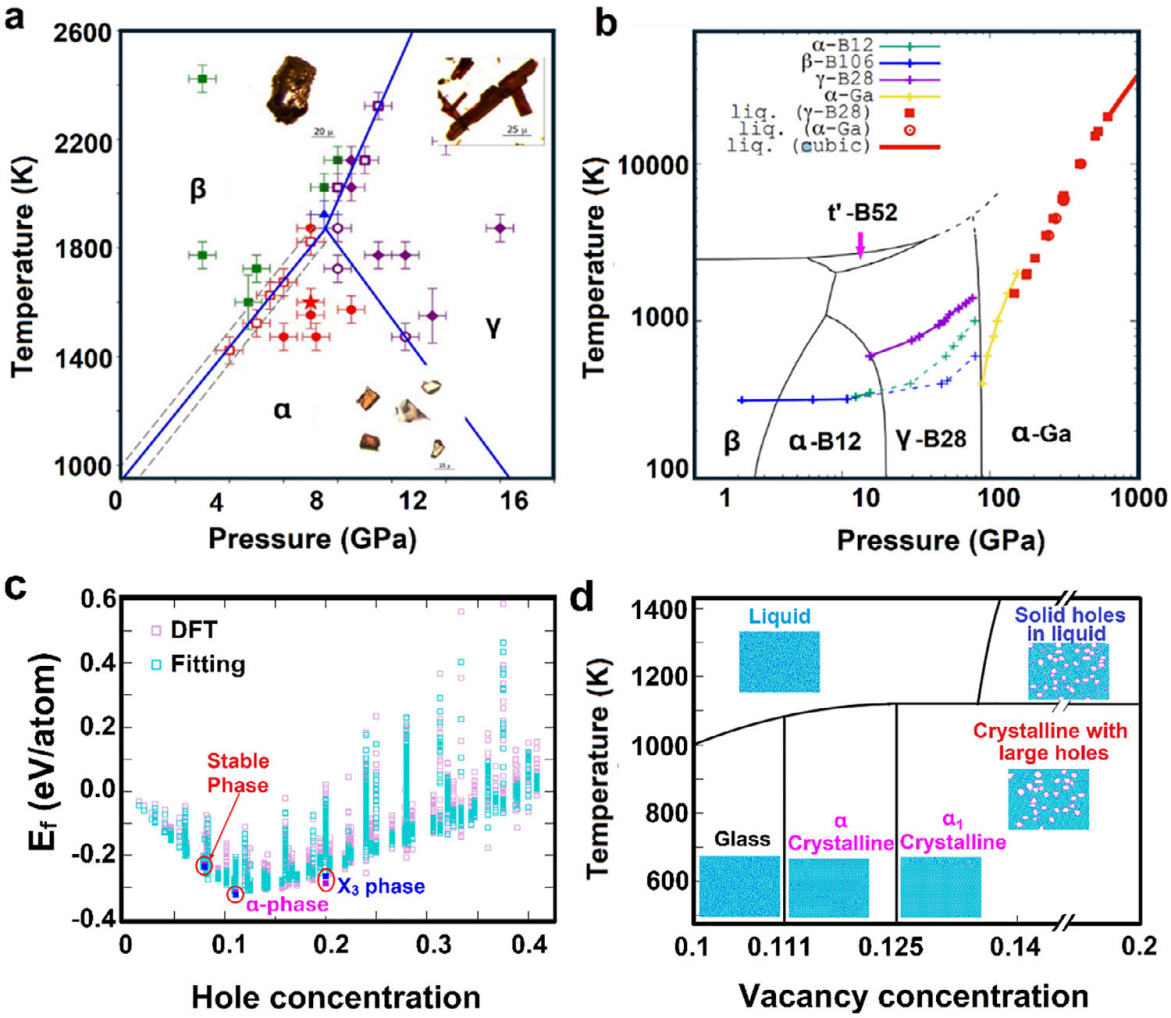
(a) Phase diagram of boron Reproduced from Ref. [89], under the terms of the Creative Commons Attribution‐NonCommercial‐NoDerivatives 3.0 License. (b) Hugoniot profiling of boron showcasing possible phase transitions [90]. Copyright 2020 Elsevier Masson SAS. All rights reserved. (c) Formation energy vs hole concentration in borophene, (d) phase evolution at various temperatures and vacancy concentrations. Reproduced by permission of The Royal Society of Chemistry [91].

Subsequent experimental work revealed the temperature‐pressure phase diagram of boron, demonstrating that specific thermodynamic conditions could selectively yield distinct crystallographic phases (Figure 4a) [89]. Hugoniot profiling of β_12_ and α‐B phases further illustrated possible phase transitions (Figure 4b) [90]. It was also shown that the formation energy and vacancy concentration are critical parameters that significantly influence the phase evolution of the borophene (Figure 4c,d) [91]. The experimental conditions, such as pressure or gate voltage applied to the substrate, can also significantly alter the crystallographic structure of the borophene. The experimental synthesis of borophene in 2015 marked a significant breakthrough in materials science. The research group of M Hersam at Northwestern University achieved the first successful growth of borophene with different structures via atomic layer deposition (ALD) on Ag(111) substrates using high‐purity boron powder (99.9999%) under ultrahigh vacuum conditions [27]. The phase purity of the borophene was controlled by the simple adjustment of the growth temperature, the rate of deposition of boron atoms, and most importantly, the template surface. Although different structures of borophene were proposed in this discovery, this landmark achievement confirmed theoretical predictions about the existence of borophene and introduced a new 2D material with distinct properties.

The structure of borophene is notably different from graphene. While graphene forms a hexagonal honeycomb lattice, the electron‐deficient nature of borophene allows it to form various crystallographic structures and hence borophene is polymorphic. Crystallographic phases of borophene include triangular, hexagonal, and chain‐like arrangements of boron atoms. These polymorphs arise from the unique bonding flexibility of boron atoms via two‐electron multi‐center (2e‐nc) bonds, with “n” ranging between 2 and 7, enabling partially filled atomic lattices. In particular, the planar triangular form of borophene has one excess electron in each unit cell of three boron atoms. The excess electron density can be balanced by removing one‐third of the boron atoms per unit cell, which will make the electron density of this form similar to graphene. Additional borophene polymorphs such as β_12_, χ_3_, and hexagonal phases were synthesized after this discovery. Each polymorph features unique atomic arrangements that influence its physical and chemical properties. For example, the ridgeline topology of the β_12_ phase enhances its electronic mobility along specific crystallographic directions i.e., along the ridgelines. In contrast, the χ_3_ phase offers a higher density of states near the Fermi level, making it suitable for electronic and catalytic applications.

The discovery of borophene, as part of the mono‐elemental “Xenes” family, represents a critical milestone in the evolution of 2D materials. Various polymorphs possess unique physical and chemical properties and offer the feasibility of material manipulation via vacancy engineering, substitutional doping, surface functionalization and 2D‐2D hybridization (out‐of‐plane orbital hybridization at the interface). These recent advances in the study of borophene provide opportunities for its applications in various domains such as in electronics, energy storage, and catalysis. The unique combination of physical properties of borophene such as high melting point, metallicity, structural anisotropy, and chemical reactivity, apart from its crystallinity, excellent electronic mobility, high thermal conductivity, strechability, optical transparency, and adaptability to material manipulation strategies such as surface functionalization or substitutional doping leading to changes in its band gap and carrier injection, sets it apart from other 2D materials. These set of extra‐ordinary behavior strengthen its role in addressing challenges where conventional nanomaterials fall short of.

## 3 Explored Approaches for the Synthesis of Borophene

**FIGURE 5:**
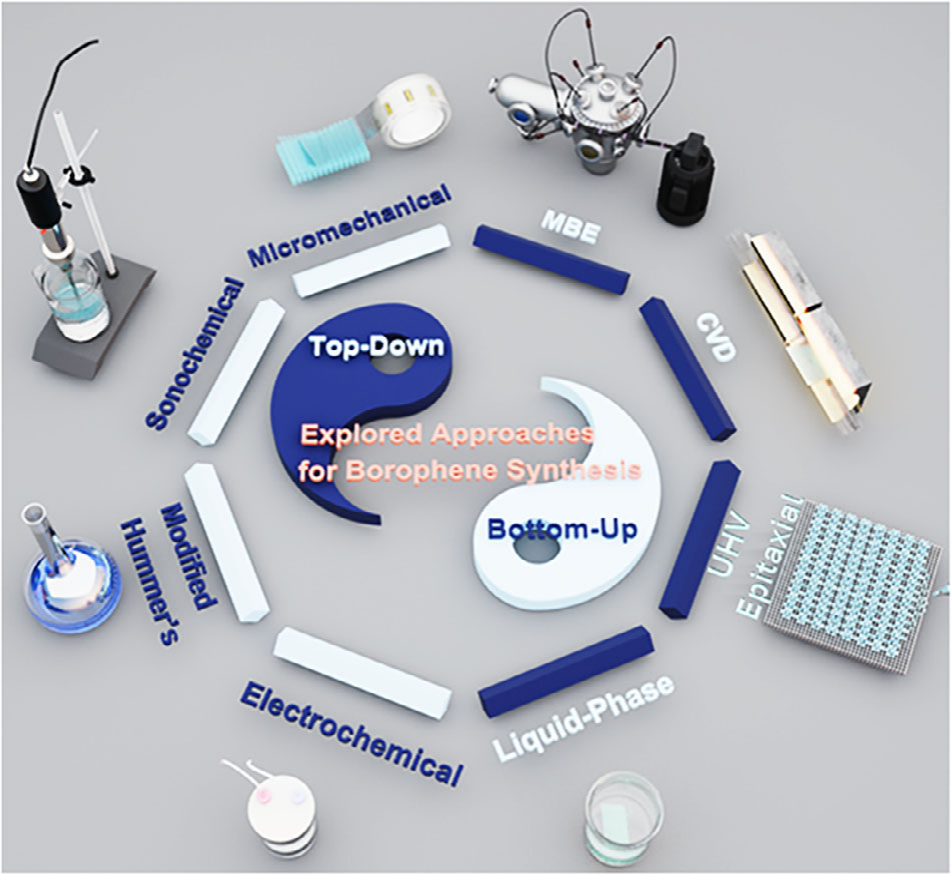
Schematic diagram depicting various explored routes for the synthesis of borophene.

Unlike graphene which has parent crystal graphite which exists in nature, borophene does not have a naturally occurring layered bulk phase from which monolayers can be easily exfoliated. Even BN or TMDCs, which are synthesized in laboratories; have their parent layered materials. Boron crystals were earlier not known to be naturally occurring in layered forms. Some theory suggests bilayers to be more stable than monolayers. Due to this background information, the synthesis of borophene has primarily focused on bottom‐up growth methods. The following section details various synthesis techniques, including substrate‐supported bottom‐up growth, top‐down exfoliation, and liquid‐phase crystal growth, each offering unique advantages and limitations. A schematic diagram depicting various methods of synthesis is shown in Figure 5. Bottom‐up molecular beam epitaxy (MBE), chemical vapor deposition (CVD), and ultra‐high vacuum (UHV) epitaxial crystal growth are substrate‐supported growth, which result in a low yield of materials and cannot be employed for applications where large amounts of materials are needed. Top‐down methods of exfoliation include micromechanical exfoliation, sonochemical exfoliation, modified Hummer's method, etc. High surface energy solvents (mixed solvents, viscous organic fluids) having high Hansen parameters are suitable to exfoliate the layers of borophene from its crystalline powder.

The synthesis of borophene remains a formidable challenge due to its substrate‐controlled growth, metastable phases, and post‐growth rapid environmental degradation. While numerous methods—ranging from ultrahigh‐vacuum epitaxy to solution‐phase exfoliation—have been explored, each technique carries inherent crystallinity, scalability, and defect density trade‐offs. Table 1 systematically compares borophene synthesis approaches, evaluating key parameters such as temperature, yield, defect density, and scalability. This analysis highlights the strengths and limitations of current methodologies, underscoring the need for innovative strategies to bridge the gap between laboratory‐scale synthesis and industrial deployment.

**TABLE 1:** Comparative Analysis of Borophene Synthesis Methods.

Method	Temperature	Defect Density	Yield	Uniformity	Scalability	Cost	Key Advantages	Key Limitations	Reference
MBE	500–800°C	Very Low	Low	High	Low	Very High	Atomic‐level control, high crystallinity	Ultra‐high vacuum, slow, substrate‐dependent	[92, 93]
CVD	800–1000°C	Moderate	Moderate	Moderate	Moderate	High	Large‐area growth, tunable phases	High precursor cost, limited defect control	[94, 95, 96, 97, 98]
UHV epitaxial	200–400°C	Low	Low	High	Low	High	Uniform thickness, conformal coatings	Slow deposition rate, low throughput	[27, 99, 100, 101, 102]
Liquid‐Phase Synthesis	25–150°C	High	High	Low	High	Low	Scalable, ambient conditions	Defect‐rich, requires post‐processing	[103]
Micromechanical Exfoliation	RT–200°C	Variable	Very Low	Poor	Low	Very Low	Simple, no specialized equipment	Uncontrolled thickness, small flake size	[30]
Sonochemical Exfoliation	RT–80°C	High	Moderate	Low	Moderate	Low	Rapid, solvent‐assisted	Oxidized edges, low crystallinity	[28, 104, 105]
Modified Hummer's Method	RT–50°C	Very High	High	Poor	High	Low	High‐yield, solution‐processable	Severe oxidation, amorphous structures	[28]

### 3.1 Bottom‐Up Growth

Bottom‐up growth methods involve atomization of molecular precursors (e.g. diborane gas) followed by borophene crystal growth on solid substrates. Techniques such as ALD, MBE, and CVD rely on precise control of deposition conditions, including substrate type, temperature, and atmosphere, to achieve specific crystal structures and polymorphs of borophene. Liquid‐phase reaction of boron containing compounds such as boric acid can also yield borophene, under a suitable set of reaction conditions. Emerging liquid‐phase growth methods further expand the scope of bottom‐up approaches, offering the potential for uniform and large‐area synthesis under controlled conditions.

#### 3.1.1 UHV Epitaxial Crystal Growth

**FIGURE 6:**
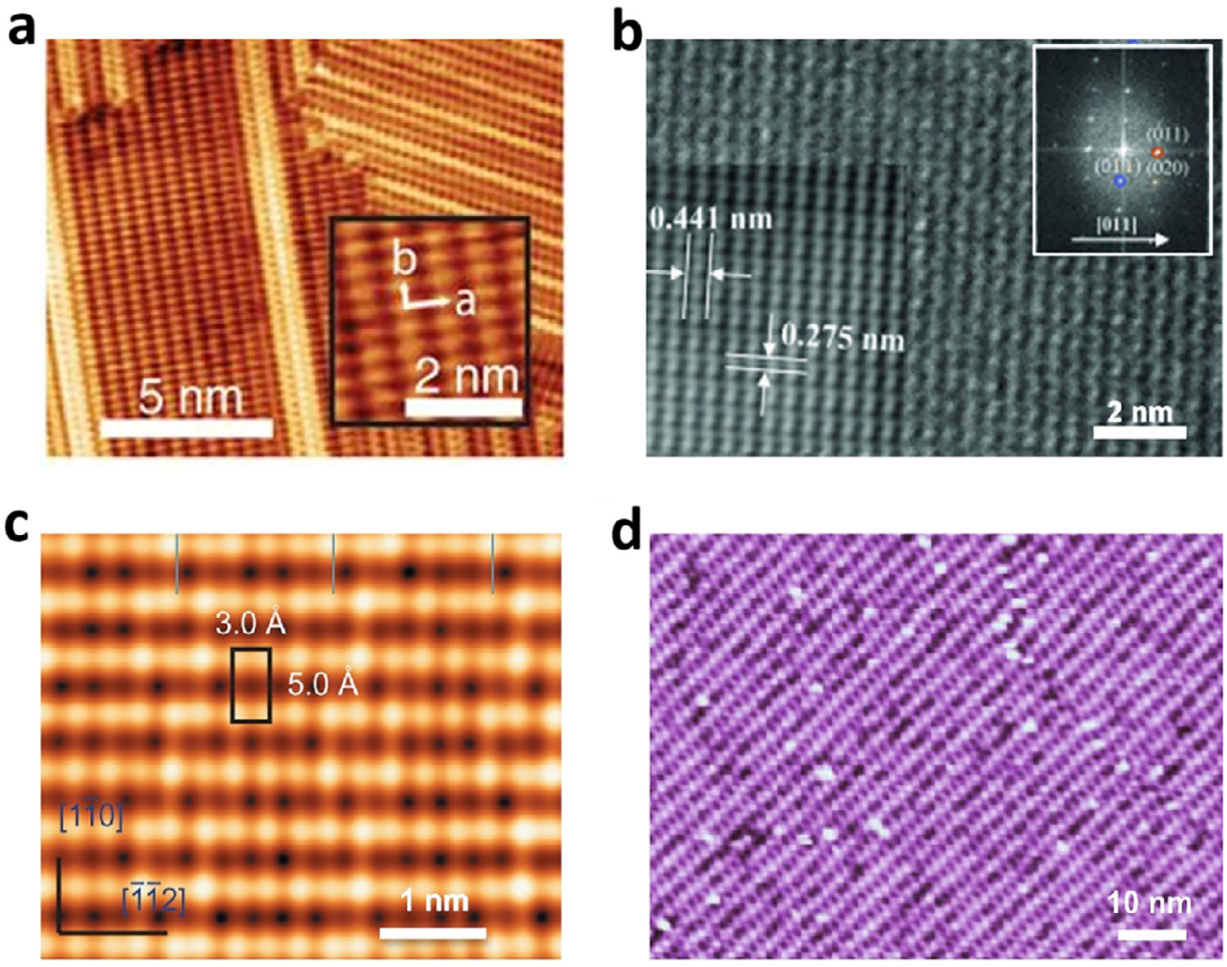
STM/TEM images of borophene synthesized via various methods: (a) *UHV Epitaxial Crystal Growth* on Ag(111) surfaces. Reproduced with permission [27] Copyright 2015, The American Association for the Advancement of Science. (b) CVD. Reproduced with permission [94]. Copyright 2015, WILEY‐VCH Verlag GmbH & Co. KGaA, Weinheim, (c) MBE on Ag(111) surfaces. Reproduced with permission [92].Copyright 2016, Macmillan Publishers Limited. All rights reserved. (d) MBE on Cu(111) surfaces. Reproduced with permission [93]. Copyright 2015, WILEY‐VCH Verlag GmbH & Co. KGaA, Weinheim.

The first experimental realization of borophene using *UHV Epitaxial Crystal Growth*, reported by Mannix et al. [27] marked a milestone in borophene research. Characterization methods such as scanning transmission electron microscopy (STEM), scanning tunneling microscopy (STM), and Auger electron spectroscopy (AES) confirmed the crystalline structure of borophene synthesized on silver (111) substrates (Figure 6a). While the role of Ag (111) substrates in determining the crystal structure of borophene has interestingly been said to be necessary, its quantification was done later by other research groups [99, 100, 101]. With the detailed experimental investigations, Hersam's group determined that the crystal phase belonged to the pmmn group. Moreover, electronic density of states (DOS) observed in the differential current (dI/dV) observed in scanning tunneling spectroscopy (STS) were found to be gapless (metallic) and corroborated sufficiently with boron sheets overlapping on the silver surface. Two distinct phases, namely homogenous and striped phases, were observed. While the homogeneous phase having atomic chains exhibited periodic buckling (0.30 nm period), the striped phase was observed with lattice parameters 0.51 and 0.29 nm, respectively, in two perpendicular directions. Even though silver has been employed as a favorable substrate, the use of gold (111) surface for borophene growth was demonstrated [102]. Unlike Ag (111), Au (111) allows the diffusion of boron atoms through its lattice and anchors the growth of the boron layer on its surface. In addition, the higher temperature (∼550°C) helps enhance the diffusion of boron atoms on the Au substrate, resulting in crystalline borophene layer formation. It should be noted that crystalline ordering of grown borophene is determined by underlying substrate crystalline order. However, atomic scale anchoring/templating breaks down and the formation of borophene islands become vivid at boron concentrations higher than a certain threshold.

#### 3.1.2 MBE Crystal Growth

MBE is the most widely used technique for synthesizing high‐quality borophene. In this approach, boron precursors are atomized, and those atoms are condensed under ultrahigh vacuum conditions onto a metal substrate (e.g., Ag, Au, or Cu), which acts as a template for boron atoms to form crystalline monolayers. Again, the choice of substrate is critical because the interaction between the boron atoms and the metal surface influences the crystallinity of resulting borophene structure. For example, borophene grown on Ag (111) surfaces tends to form β_12_ and χ_3_ polymorphs, while other substrates may promote the formation of different structures (Figure 6c) [92]. While the β_12_ phase grew at a lower temperature of ∼570 K, a higher temperature of ∼650 K led to the formation of the X_3_ phase. Thus, lower growth temperatures give rise to the anisotropy in grown crystal, however; there is a minimum threshold temperature above which crystallization takes place. STM and first principle calculations were employed to establish the crystallographic phases of grown borophene sheets. The stacked bilayer of borophene was first grown by Chen et al. via MBE (Figure 6d) [93]. Charge transfer at the interface with Cu (111) substrate played a crucial role in bilayer growth. MBE offers advantages such as atomic‐level control over growth conditions and the ability to produce large‐area monolayers with distinct polymorphs. However, the process requires sophisticated equipment and ultrahigh vacuum conditions, apart from precise temperature control, which can limit scalability. Like *UHV Epitaxial Crystal Growth* technique, crystalline order of MBE grown borophene is primarily dictated by the substrate crystal structure. Therefore, different crystal surface would lead to different crystallographic structures of grown borophene. Apart from the substrates, growth temperature and precursor dose would determine crystalline order of borophene.

#### 3.1.3 CVD Growth of Borophene on Various Substrates

CVD involves decomposing a boron‐containing precursor gas (e.g., diborane) onto a heated substrate under a controlled atmosphere. The choice of substrate, reaction temperature, and carrier gas flow rates can be suitably tailored to promote the growth of borophene crystalline sheets with specific crystal structures. Tai et al. first used CVD to grow γ‐B_28_ monolayer borophene on copper foil (Figure 6b) [94]. The reaction of B_2_O_3_ and B at 1000°C reduced B_2_O_3_ to boron atoms, which grew as layers on the molten copper surface in the presence of hydrogen gas. Grown borophene was semiconducting in nature and exhibited a band gap of 2.25 eV. Characteristic Raman peak was observed at 618 cm^−1^, whereas the B 1s XPS peak was recorded at 187.6 eV. These observations, along with theoretical calculations, established the growth of γ‐borophene. Further, the thermal decomposition of diborane has been reported to form borophene on the Al (111) surface [95]. GaAs, quartz, mica, and several other substrates have been employed to grow borophene [96, 97, 98]. Borophene with different properties can be fabricated depending on the substrate. For example, when grown on GaAs substrate, multilayered borophene with higher carrier mobility than monolayer borophene was observed. On the other hand, the growth of the borophene on the insulating surface, quartz, offered the borophene glass with a bandgap of 2.48 eV, which showed high photosensitivity (0.31mA∙W^−1^) and fast response (117 ms) for the photodetector [96]. In contrast to *UHV Epitaxial Crystal Growth* and MBE, CVD offers a cost‐effective and scalable method for borophene synthesis.

#### 3.1.4 Liquid‐Phase Bottom‐Up Crystal Growth

Liquid‐phase synthesis involves growing borophene from a boron‐containing precursor in a liquid medium, often with a metal catalyst that promotes nucleation and growth. This bottom‐up approach can be advantageous for producing borophene with a high degree of crystallinity over large areas, as the liquid medium can facilitate uniform distribution of boron atoms during the crystal growth process. Sharma et al. have reported using H_3_BO_3_ and CTAB surfactant to grow borophene which exhibits a high band gap of 2.32 eV, high piezoelectric charge coefficient (d_33_) of 86 pm V^−^^1^, high dielectric constant (125) at low frequency, and blue light emission under UV light illumination [103]. However, the bottom‐up methods for the synthesis of borophene in the liquid phase are at a nascent stage. The use of various transition metal catalysts, the effect of solvents, surfactants, thermodynamic (T+P) conditions, etc., need to be systematically explored in detail. Optimized sets of parameters are expected to yield highly crystalline borophene monolayers. However, much research and development are required before this new synthesis method is scaled up for targeted applications.

### 3.2 Top‐Down Exfoliation

Source of energy with sufficient energy to overcome inter‐layer interaction between boron layers in parent boron crystals, can lead to efficient exfoliation of layers. While physical exfoliation strategies include micromechanical, and sonochemical methods, modified Hummer's method of exfoliation involves suitable chemical reaction of solid mixture of boron powder with KMnO_4_ in mixed acid media containing sulfuric as well as phosphoric acids. These approaches provide alternative pathways for synthesizing borophene, opening up opportunity for large scale commercial production.

#### 3.2.1 Micromechanical Exfoliation

**FIGURE 7:**
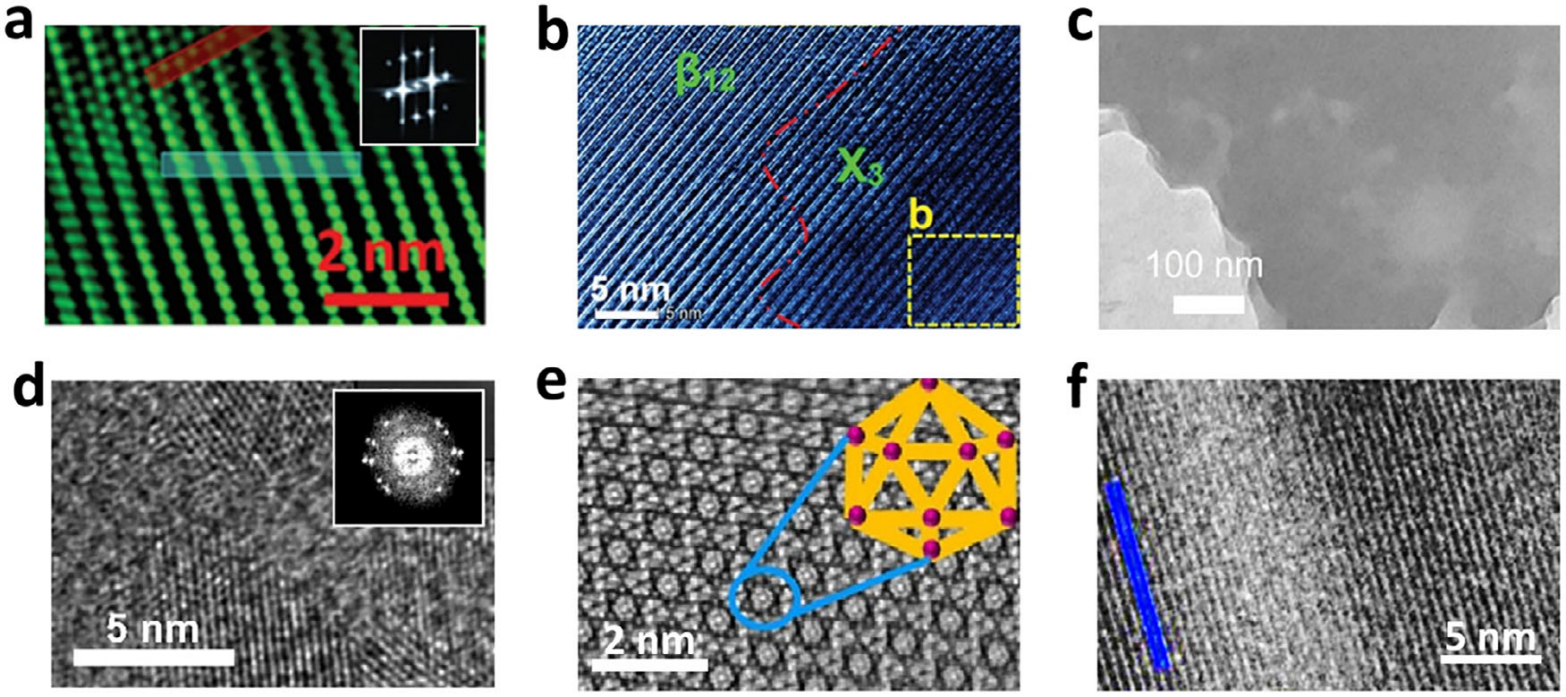
TEM images of (a) micromechanical exfoliation. Reproduced with permission [30] Copyright 2021 Wiley‐VCH GmbH. (b) sonochemical exfoliation. Reproduced with permission. [28] Copyright 2019 WILEY‐VCH Verlag GmbH & Co. KGaA, Weinheim.(c) ion‐exchange method resulting in hydrogenated borophene. Reproduced under the terms of the CC‐BY Creative Commons Attribution 4.0 International license (https://creativecommons.org/licenses/by/4.0). [108] (d) electrochemical exfoliation. Reproduced under the terms of the CC‐BY Creative Commons Attribution 4.0 International license (https://creativecommons.org/licenses/by/4.0) [109]. (e) ball‐milling and sonication‐assisted liquid exfoliation. Reproduced with permission. [110]Copyright 2023 Elsevier B.V. (f) molten‐salt method. Reproduced with permission. [111].

Micromechanical exfoliation, also known as the “Scotch tape” method, has been used for fabrication of borophene. The process involves applying strong adhesive forces to peel off thin layers of material, which can be challenging given that boron does not naturally exist in a layered form i.e. there is not a graphite analogue found in nature. Therefore, this process relies on crystalline order and inter‐layer coupling in boron crystals used for exfoliation. Growth conditions of such parent crystal often determine their exfoliability. Structural and morphological modulations are however, feasible. The first demonstration of micromechanical exfoliation to achieve mono, as well as a few layered borophene, was reported by Chahal et al. in 2021 [30]. Crystalline quality was very high, and oxidation was minimal compared to that from other earlier reports (Figure 7a). Detailed investigations were carried out on transferred borophene layers employing HRTEM, XPS, Raman, and other characterization tools. Molecular dynamics was used to understand the transfer process. The transferred layer follows the parent crystal nature, primarily the β_12_ phase. However, strain‐mediated corrugations and atomistic ripple formation were reported. Interestingly, when the number of layers was cut down to monolayers, the number of Raman peaks gradually reduced. The AFM line profile at the monolayer revealed ∼0.4 nm thickness at the edge. This thickness includes atomic diameter and boron atoms’ distance from the substrate surface. Borophene‐based hetero‐layered excitonic devices were also fabricated using MoS_2_ and phosphorene as companion layers. Excitonic light energy matching with the band gap of the companion layers was observed. In such heterolayered excitonic devices, borophene, a high electronic mobility material, assumes significance in ultrafast sensing of toxic gases, diabetic and early‐stage cancer diagnosis etc. Although this method can produce high‐quality, single‐layer borophene, it is unsuitable for large‐scale production due to its labor‐intensive nature and low yield.

#### 3.2.2 Sonochemical Exfoliation

The ultrasonic waves generate cavitation bubbles that produce localized high‐pressure and high‐temperature conditions, promoting the exfoliation of atom‐thin borophene sheets. This method offers the advantage of being relatively simple and scalable. Sonochemical exfoliation of borophene was demonstrated by Ranjan et al. [28] (Figure 7b) and Li et al. [104]. Various solvents, such as DI water, isopropyl alcohol, acetone, dimethylformamide (DMF), etc., were employed for exfoliating boron layers. Hansen parameters of solvents determining surface energy are crucial and held responsible for exfoliation efficiency [105]. For example, dimethylsulfoxide (DMSO), N‐methyl‐2‐pyrrolidone (NMP), etc., have higher Hansen parameters. While DMF, IPA, and acetone have medium Hansen parameter values, and water has the lowest one. On the other hand, low boiling point solvents generate more cavities. Another crucial consideration when selecting solvents is that they should be a reducing solvent so that formed borophene does not oxidize during synthesis. In that regard, DMF and NMP are better. Vacuum drying is prescribed for DMF solvents. While it takes several days to achieve monolayers in IPA solvent, it takes ∼24 h in NMP to obtain effective exfoliation in an ultrasonic bath. Sonic power and sonic frequency are also vital parameters apart from the choice of solvents. A high‐power sonicator can fragment borophene sheets rather than exfoliate them. Higher frequency used, on the other hand, can give rise to improved cavitation effects. Borophene‐based hetero‐layered devices with companion layer MoS_2_ and BN were fabricated, and digital electronic signatures were obtained [32]. However, controlling the size and thickness of the exfoliated borophene sheets remains challenging, and the quality of the borophene produced through sonochemical methods may be lower than that obtained through bottom‐up *UHV Epitaxial Crystal Growth* and MBE approaches.

#### 3.2.3 Modified Hummer's Approach

The modified Hummer's approach, commonly used for the oxygenation of graphite to produce graphene oxide, has also been adapted to exfoliate boron layers from crystalline boron powder [28]. This method involves intercalation of solvents and oxidizing agents and surface functionalization (=O, ‐O‐, ‐OH) of boron layers underneath to weaken the interlayer coupling between boron layers within the bulk crystal, followed by sonication to produce borophene sheets. Since boron oxidation is an exothermic chemical process, cooling down both acid mixtures (H_2_SO_4_ and H_3_PO_4_) and solid mixture (boron powder and KMnO_4_) in a fridge for a few hours helps in controlling the otherwise vigorous reaction. Interestingly, thick smoke comes from the solution containing borophene sheets. Collecting the thick smoke suffices, as the material is already dried and can instantly be used for further characterizations. This method is highly scalable compared to other top‐down methods. Introducing chemical functional groups during the oxidation process can also provide a means for further functionalizing the borophene, potentially enhancing its stability and reactivity. Borophene oxide can be employed to achieve doped borophene samples as well. However, controlling the oxidation process to avoid excessive damage to the borophene lattice structure is a significant challenge one needs to address [106, 107].

#### 3.2.4 Other Methods

The synthesis of borophene using alternative top‐down methods showcases the versatility of these approaches in achieving diverse structural outcomes. For example, the ion exchange method for hydrogenated borophene emphasizes using proton exchange with magnesium cations in MgB_2_, forming boron sheets bridged by hydrogen atoms. This method facilitates the production of ultrathin borophene, which exhibits a unique lamellar morphology and strong catalytic properties due to its high surface area and structural integrity (Figure 7c) [108]. (h) Electrochemical exfoliation involves embedding bulk boron powder into metal meshes (e.g., copper or nickel) and applying a specific current in different electrolytes, resulting in few‐layered borophene with well‐defined crystalline structures and varying thicknesses, as validated by TEM (Figure 7d) [109]. This scalable method allows for the production of high‐quality borophene with controlled properties. The combined ball‐milling and sonication technique highlights high‐energy mechanical grinding followed by ultrasonic treatment in a liquid medium to exfoliate bulk boron into few‐layer nanosheets. The resultant borophene demonstrates uniform atomic arrangements and distinct crystalline phases, as evidenced by the high‐resolution TEM images showcasing its well‐preserved atomic structure (Figure 7e) [110]. The molten‐salt method represents another innovative approach, where AlB_2_ and CuCl_2_ are mixed and calcined under nitrogen atmosphere in a two‐stage process (300°C for 2 h, then 700°C for 4 h). Subsequent washing with deionized water and ammonium persulfate removes impurities and copper particles, yielding high‐quality borophene with an average thickness of 3.5 nm. TEM analysis confirms the borophene's accordion‐like morphology and crystalline β_12_ phase with an interplanar distance of 0.504 nm (Figure 7f) [111]. These methods collectively offer promising pathways for the large‐scale synthesis of borophene tailored for specific applications. However, the structural analysis and applications of these materials are a huge issue because they get easily oxidized.

## 4 Properties of Borophene

**FIGURE 8:**
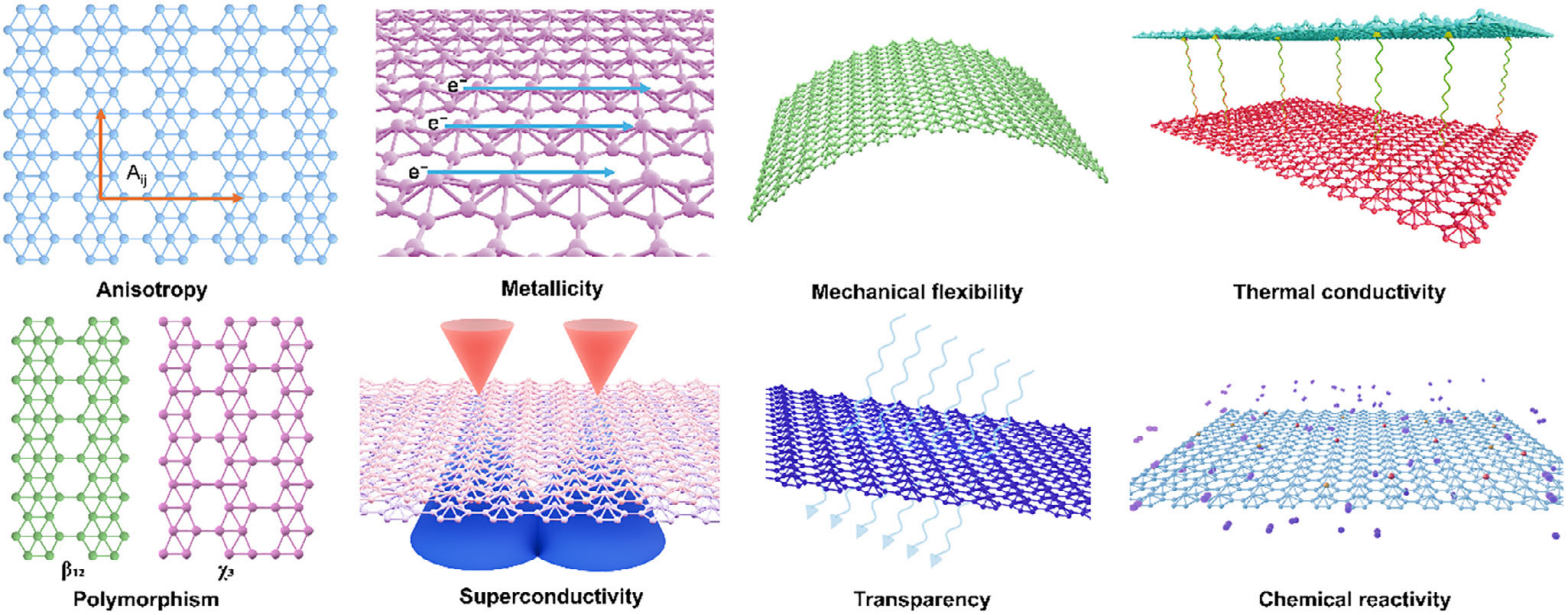
Illustration of the key properties of borophene.

Borophene stands out among 2D materials due to its extraordinary combination of electronic, optical, thermal, elastic, and chemical properties. The number of electron per boron atom being less than that of carbon, giving rise to 2e‐nc bonds with various ways to bond, both in‐plane and out‐of‐plane. Such freedom of bond formation results in a unique capability to form a set of versatile crystallographic structural phases, offering exceptional opportunities for diverse technological applications. Table 2 provides a comparative overview of borophene's properties alongside other 2D materials, illustrating its distinctive position among quantum materials. To illustrate the diverse properties of borophene mentioned in this section, Figure 8 provides a comprehensive overview of its key characteristics.

**TABLE 2:** Comparison of Properties of Borophene and Other 2D Materials.

Material	Electrical conductivity (S/m)	Thermal conductivity (W/mK)	Mechanical strength (GPa)	Chemical reactivity	Band gap (eV)	Reference
Graphene	∼10^7^–10^8^	∼5000	∼130	Low (chemically inert)	∼0 (semimetal)	[16, 112]
Phosphorene	∼10^4^	∼12–30 (anisotropic)	∼0.5–1	Moderate (reactive in air)	∼0.3–2 (tunable)	[113, 114]
Boron Nitride (BN), GaN	∼10^−12^ (BN), ∼10^3^ (GaN)	∼200–400 (BN)	∼33–45 (BN)	Low (BN is stable)	∼5.5 (BN), ∼3.4 (GaN)	[115, 116]
TMDCs (e.g., MoS_2_)	∼10–10^4^	∼30–110	∼20–30	Moderate (depends on composition)	∼1.2–2.5 (direct in monolayers)	[117, 118]
MXenes	∼10^5^–10^6^	∼5–50	∼25–30	High (surface terminations active)	∼0.1–2.0 (tunable)	[119, 120, 121]
2D Metal Oxides (2DTMOs)	∼10^−5^–10^2^	∼1–50	∼10–30	Moderate to High	∼1.0–3.5	[122, 123]
2D MBenes	∼10^4^–10^5^	∼5–30	∼20–25	High	∼0.5–1.5	[124, 125]
Perovskite Nanosheets	∼10^−1^–10^3^	∼0.5–1.5	∼0.01 ‐ 0.1	High (degrades in moisture)	∼1.5–2.3	[126, 127]
Silicene, Germanene, Stanene	∼10–10^3^	∼10–40 (anisotropic)	∼0.05–1 (Silicene), ∼0.1–0.5 (Germanene), ∼0.05–0.2 (Stanene)	Moderate	∼0.1–1.0 (tunable)	[128, 129, 130]
Antimonene, Tellurene	∼10–10^3^	∼2–50 (anisotropic, Tellurene)	∼0.1–4	High (depends on phase)	∼0.4–1.2 (tunable)	[131, 132, 133]
Borophene	∼10^6^–10^7^	∼200–400	∼150 (superior to graphene)	High (high surface reactivity)	∼0 (metallic phases), ∼1 (engineered)	[27, 73, 134]

### 4.1 Electronic Properties

The electronic properties of borophene have been a focal point of research due to its intrinsic metallic nature and exceptionally high electrionic mobility. Bilayer borophene has been theoretically predicted to have semiconducting behaviour [135]. Stacking sequences AA and AB are supposed to exhibit distinct characteristics. Unlike many other 2D materials, borophene exhibits anisotropic metallic behavior. The electronic characteristics, such as resistivity, are susceptible to carrier concentration in borophene [136]. Thus, various means of nanoarchitectonics, such as doping, 2D‐2D/2D‐3D/2D‐1D/2D‐0D hybridization, surface functionalization, defect generation, etc., can lead to desirable electronic behavior. Its electronic mobility can vary depending on the crystallographic direction, which is associated with the unique arrangement of boron atoms in different polymorphs [137]. This anisotropy results from the local electron density distribution, which can be modified by changing the polymorph or introducing lattice defects. The structural transition in borophene from one phase to the other is exciting. For example, the β_12_ and χ_3_ polymorphs demonstrate variations in the density of states at the Fermi level, which can influence electronic transport properties. The low electron effective mass in borophene suggests its potential for applications in ultra‐fast electronic devices where rapid electron mobility is crucial. Hydrogenation is one of the possible ways to stabilize the structure of the formed borophene, and this has been realized on various substrates with stabilized borophene structures, which offer tunable electronic properties [138, 139] Moreover, the electronic band structure of borophene can be tailored through doping, strain engineering, or functionalization [140], enabling the development of electronic components such as field‐effect transistors [141], sensors [142], and other nanoscale devices.

### 4.2 Optical Properties

The optical properties of borophene are equally remarkable, characterized by anisotropic absorption and significant optical activity in the electromagnetic spectrum's UV–vis region [143, 144, 145]. Its absorption spectra reveal directional dependence due to its anisotropic electronic structure, which uniquely allows borophene to interact with polarized light. This characteristic makes borophene highly suitable for applications in photonic and optoelectronic devices [146], such as photodetectors [147], modulators, and components for integrated optical circuits. Tuning the optical response of borophene by altering its polymorphic structure, introducing defects, or applying strain provides additional degrees of freedom in designing advanced optical materials [148].

### 4.3 Thermal Properties

Borophene also exhibits outstanding thermal properties, with a thermal conductivity comparable to or exceeding that of graphene [75, 149] This high thermal conductivity, which is anisotropic in nature, arises from the efficient phonon transport across the 2D lattice, makes borophene an excellent material for heat dissipation in electronic devices, especially in high‐performance computing and thermal management systems. Its ability to maintain structural integrity under elevated temperatures and high thermal diffusivity further supports its use in thermal interface materials and flexible heat spreaders. Furthermore, the thermal conductivity of borophene can be modulated by selecting different polymorphs, introducing specific defects, or their hybridization [150], offering a versatile approach to optimizing thermal management in nanodevices. Metal intercalation can also result in superior thermal conductance [151].

### 4.4 Elastic Properties

The mechanical properties of borophene are extraordinary, combining high flexibility and tensile strength that surpass those of graphene [152]. Its auxetic and ferroelastic nature and strain‐tunable electronic transitions enable diverse applications in flexible electronics and soft robotics [153, 154] This combination of mechanical strength and flexibility is a consequence of its unique structural design in its lattices, where different polymorphs exhibit variations in bonding that can accommodate significant deformation without structural failure [152]. For instance, the β_12_ polymorph demonstrates enhanced tensile strength due to the arrangement of hexagonal and triangular boron motifs, which can redistribute mechanical stress. The ability of borophene to sustain high strain levels makes it an ideal candidate for flexible and stretchable electronic applications, including wearable technology, foldable displays, and soft robotics. Moreover, its mechanical properties can be further tuned through defect generation [155], doping with other elements, surface functionalization, or the formation of heterostructures (hybridization), broadening the potential for borophene‐based mechanical devices [156].

### 4.5 Chemical Properties

The high chemical reactivity of borophene arises from its large surface area and the presence of numerous active sites, particularly at atomic vacancies or edges, which can participate in chemical reactions [73, 157] Metallic nature, extraordinary chemical binding through 2e‐nc bonds, and surface activity via π‐π interactions are the major advantages of borophene. The polymorphic nature of borophene allows for different configurations of reactive sites, making it a versatile catalyst. For example, borophene has demonstrated superior performance in some catalytic processes compared to conventional metal‐based catalysts due to its higher density of active sites and more efficient charge transfer capabilities. The high reactivity of borophene can also be advantageous for applications in chemical sensing, where the adsorption of target molecules on the surface alters its electrical properties, providing a means for detection. However, high reactivity challenges stability under ambient conditions, requiring protective coating or encapsulation strategies to prevent oxidation and degradation. Borophene exhibits polyphasic features, with most phases having different vacancy concentrations. Incidentally, the vacancy superlattice and their distribution decide individual borophene layers' physical and chemical properties. Structure‐property relations are, therefore, very crucial in determining the way borophene behaves, as the number of edge atoms (acting as electrochemically active sites) depends on the number and arrangement of vacancies in the borophene lattice. Interestingly, anisotropic phases exhibit superior electronic/thermal transport and mechanical behavior along the ridgelines compared to those in the transverse directions.

In summary, borophene differs from most other 2D materials in its properties because of its unique electron‐deficient bonding and polymorphic structure. It is intrinsically metallic, whereas many 2D materials are semiconducting or semi‐metallic. Borophene shows strong in‐plane anisotropy, so its electrical, mechanical, and thermal properties depend strongly on direction, unlike isotropic materials such as graphene. It also exhibits exceptionally high mechanical strength and flexibility along certain directions, high electrical conductivity, and enhanced chemical reactivity due to its vacancy‐rich structure. In contrast, most other 2D materials have more uniform lattices, weaker anisotropy, and less tunable metallic behavior.

## 5 Applications of borophene

Borophene, with its distinctive electronic, mechanical, and chemical properties, has shown great potential for various applications beyond the capabilities of traditional materials. The unique combination of high electronic mobility, mechanical flexibility, and surface reactivity enables borophene to perform exceptionally well in fields such as electronics, energy storage, and catalysis, where existing materials face limitations. The following sections discuss the most promising applications of borophene that have already been explored in experimental and theoretical studies.

### 5.1 Electronics

Borophene has exceptional properties, including high electronic mobility, mechanical flexibility, and anisotropic behavior, which makes it a promising candidate for various electronic applications.

This table evaluates 2D materials for electronic applications, focusing on electron mobility, band gap, and thermal conductivity (Table 3).

**TABLE 3:** 2D Materials for Electronics.

Material	Electron mobility (cm^2^/V·s)	Young's modulus (GPa)	Band gap (eV)	Flexibility	Thermal conductivity (W/mK)	Reference
Graphene	∼15 000–200 000	∼130	∼0	High	∼5000	[158, 159]
Silicene	∼1000–10 000	∼0.05–1	∼0.1–1.0	Moderate	∼10–40	[128]
Phosphorene	∼1000	∼0.5–1	∼0.3–2.0	Low	∼12–30	[160]
Stanene	∼2000–5000	∼0.1–0.5	∼0.1–0.3	Moderate	∼10–20	[161, 162]
Germanene	∼10 000–20 000	∼0.1–0.5	∼0.1–0.5	Moderate	∼10–30	[163]
InSe	∼1000–2000	∼10–20	∼1.2–1.4	Moderate	∼10–20	[164, 165]
ReS_2_	∼10–100	∼20–30	∼1.5–1.6	Low	∼5–10	[166]
Borophene	∼10 000–20 000	∼150	∼0 (metallic), ∼1 (engineered)	High	∼200–400	[167]

#### 5.1.1 Borophene‐Based Nanogenerators

The high charge density, mechanical flexibility, and anisotropic properties of borophene promise its huge potential in energy harvesting applications. Researchers have achieved significant advancements in energy conversion efficiency and application versatility by integrating borophene into triboelectric and piezoelectric nanogenerators (TENGs and PENGs) [103, 168, 169].

Borophene‐based composites have demonstrated remarkable performance enhancements in triboelectric nanogenerators. For instance, borophene/polyvinylidene fluoride (PVDF) hybrid nanofibers fabricated using electrospinning achieved an open‐circuit voltage of 102.5 V and a short‐circuit current of 0.8 µA, with a power density of 0.08 W/m^2^. This configuration effectively powers small electronics, such as LEDs and calculators. Furthermore, a single‐electrode TENG utilizing these nanofibers could harvest energy from raindrops, generating 13 V, showcasing its versatility for environmental energy harvesting [169]. Similarly, borophene/ecoflex composites were used to create durable fabric‐based TENGs (B‐TENGs) for healthcare applications. These devices demonstrated resilience under deformation and washing, powering systems like medical assistive interfaces and wound healing therapies [168].

The non‐centrosymmetric lattice structure of borophene has unlocked its potential for flexible piezoelectric nanogenerators in piezoelectric applications. Few‐layered borophene nanosheets embedded in polydimethylsiloxane (PDMS) exhibited a high piezoelectric charge coefficient (d33) of 86 pm/V, producing an output voltage of 8 V under mechanical stress. The device remained functional after over 1250 cycles, emphasizing its reliability for long‐term applications. Especially, the blue light emission of borophene under UV illumination enhanced the performance of device, opening avenues for piezophototronic applications [103].

Despite these advancements, challenges remain in scaling borophene synthesis and addressing its environmental sensitivity. Therefore, continued efforts in functionalization and large‐scale manufacturing are essential to overcome these hurdles. Further, integrating borophene into nanogenerators represents a significant step forward, offering sustainable solutions for powering wearable electronics, healthcare technologies, and environmental sensors, highlighting its transformative potential in energy harvesting systems.

#### 5.1.2 Sensors and Detectors

As borophene has extraordinary surface area, high conductivity, and tunable electronic properties, it emerged as a promising material for advanced sensor and detector applications. In gas sensing, the high sensitivity and selectivity of borophene have enabled the detection of toxic gases such as NO_X_ and SO_X_ at trace levels. Recent studies have developed dual‐mode gas sensors using borophene, which combine chemoresistance and electrochemical capabilities to enhance sensitivity and response times. These advancements highlight the potential of borophene for environmental monitoring and industrial applications [170, 171].

**FIGURE 9:**
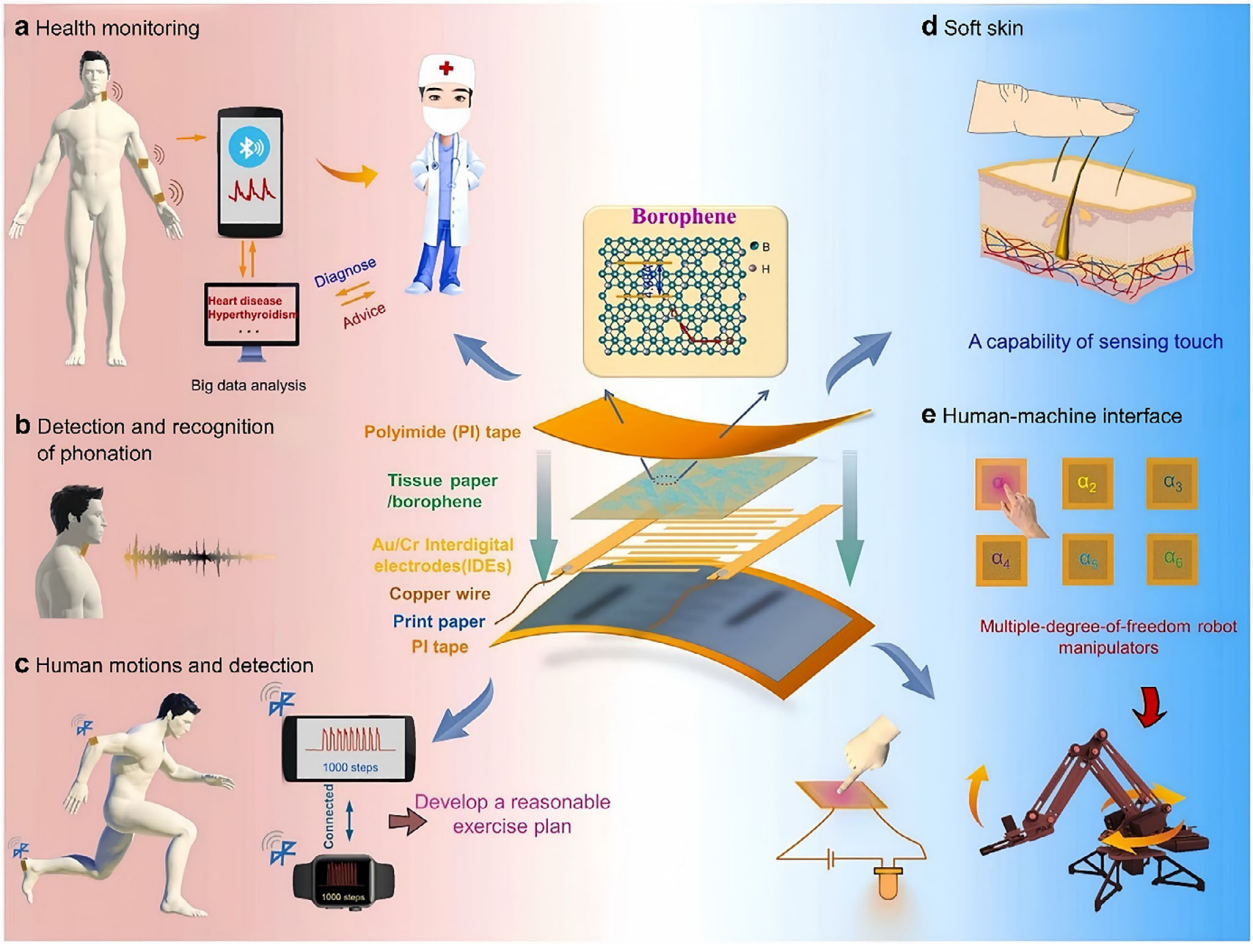
Applications of borophene‐based pressure sensors: (a) health monitoring for disease diagnosis, (b) phonation detection for speech recognition, (c) human motion tracking, (d) soft electronic skin for tactile sensing, and (e) human‐machine interfaces enabling robotic control. Reproduced with permission. [172].Copyright 2022 Elsevier Ltd.

In wearable pressure sensing, hydrogenated borophene has been utilized to fabricate flexible sensors with broad detection ranges (0–120 kPa), high sensitivity (2.16 kPa^−^^1^), low power consumption (∼0.6 µW), and excellent reproducibility over 1000 cycles. These sensors have demonstrated applications in health monitoring, electronic skin, human‐machine interfaces, and robotics, paving the way for the integration of borophene into advanced multifunctional wearable systems (Figure 9) [172].

In electrochemical sensors, borophene has been successfully integrated with materials like nickel phthalocyanine (NiPc) to enhance performance in electrochemical glucose sensing. The resulting NiPc‐borophene nanocomposites achieved sensitivity levels as high as 10.31 µA mM^−^^1^ cm^−^^2^ with a meager detection limit of 0.15 µM, demonstrating the role of borophene in overcoming the limitations of traditional materials like NiPc alone [173]. Similarly, the incorporation of borophene with copper phthalocyanine (CuPc) has enhanced the sensitivity of urea biosensors, reaching detection limits of 0.05 µM in complex biological matrices [174, 175, 176].

In dopamine detection, PANI‐borophene nanocomposites outperformed traditional PANI‐based sensors by achieving a sensitivity of 385.05 µA µM^−^^1^ cm^−^^2^ and an impressive detection limit of 0.017 µM. This improvement is attributed to the ability of borophene to enhance the redox interactions at the electrode surface, making it an excellent candidate for biomedical applications [177].

The unique hydrophilic and electronic properties of borophene have also enabled high‐performance humidity sensors. These devices demonstrated rapid response (28.8 s) and recovery times (2.6 s) across a wide detection range (11%–97% RH). Applications include advanced human‐centric systems like respiratory monitoring and speech recognition, paving the way for integration into wearable electronics and healthcare devices [178]. Furthermore, the integration of borophene into paper‐based electrodes for protein biomarker detection has yielded highly flexible, durable sensors and eco‐friendly disposal methods, making them ideal for point‐of‐care diagnostics [179].

In optoelectronics, borophene grown on mica substrates via van der Waals epitaxy has enabled the development of high‐performance photodetectors. These devices achieved superior photoresponsivity (1.04 AW^−^^1^) and detectivity (1.27 × 10^1^ ^1^ Jones), outperforming conventional 2D materials in photodetection applications [98]. Despite its promising properties, the practical application of borophene in electronic chips faces challenges such as environmental instability, lack of protocols for large‐scale synthesis, and integration with existing semiconductor processing technologies. These limitations can be addressed through functionalization and innovative manufacturing techniques, which will unlock the potential of borophene in sensor technologies. Recently, much research is focused on developing protective coatings, optimizing synthesis methods, and exploring borophene‐based heterostructures. Advancements in these areas, with the continued experimental research and technological development, are expected to facilitate the incorporation of borophene into next‐generation electronic devices.

### 5.2 Information Storage

The remarkable physical and electronic properties, including unique anisotropic electronic structure, high electron mobility, and exceptional stability under optimized conditions of borophene, have also opened new avenues for its application in advanced information storage technologies and next‐generation memory devices.

This table compares borophene with other 2D materials based on properties relevant to information storage applications (Table 4).

**TABLE 4:** 2D Materials for Information Storage Applications.

Material	Electrical conductivity	Mechanical flexibility	Structural uniqueness	Anisotropic properties	Potential memory mechanism	Reference
Borophene	High	High	Vacancy‐mediated configurations	Yes	Resistive switching (speculative)	[167]
Graphene	Very High	High	Hexagonal lattice	No	Various (e.g., flash, RRAM)	[180]
MoS_2_	Moderate (semiconductor)	Low	Layered structure	Yes	Charge trapping, floating gate	[181]
hBN	Insulator	Moderate	Hexagonal lattice	No	Dielectric, resistive switching	[182]
Black Phosphorus	Moderate (semiconductor)	Low	Puckered structure	Yes	Charge trapping, floating gate	[183]
MXenes	High	High	Layered carbides/nitrides	Yes	Resistive switching, electrodes	[40]
α‐In_2_Se_3_	Moderate (semiconductor)	Moderate	Layered ferroelectric	Yes	Ferroelectric polarization	[184]
WSe_2_	Moderate (semiconductor)	Low	Layered structure	Yes	Charge trapping	[185]
Stanene	High (topological)	High	Buckled honeycomb	Yes	Resistive switching	[186]
Silicene	Moderate (semiconductor)	High	Buckled honeycomb	Yes	Resistive switching	[187]

#### 5.2.1 Memory Device Architectures and Performance

**FIGURE 10:**
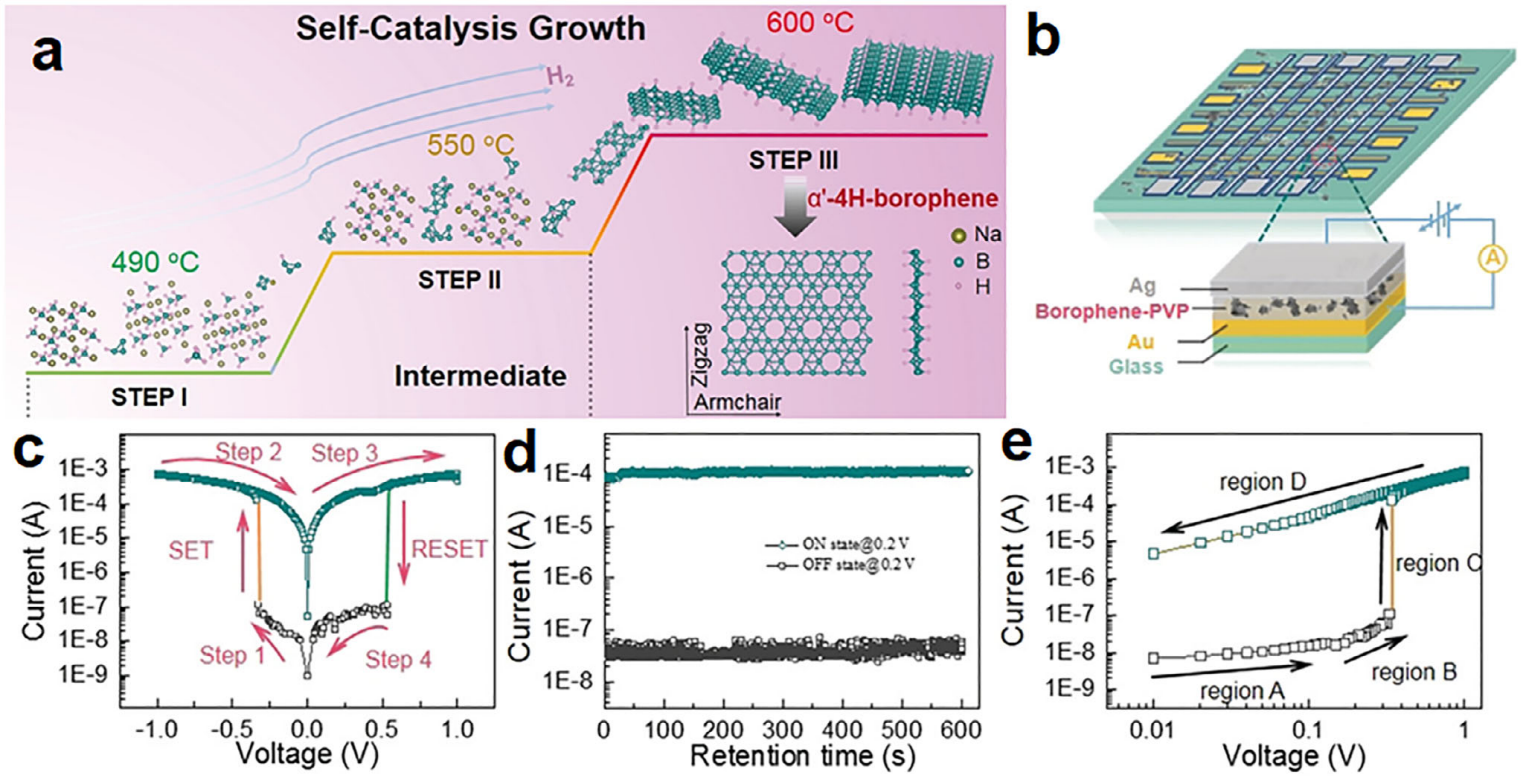
(a) Schematic representation of the in‐situ stepwise thermal decomposition process of NaBH_4_ to synthesize α'‐4H‐borophene through self‐catalysis. (b) Illustration of the Au/borophene–PVP/Ag/glass resistive switching memory device structure. (c) Current‐voltage (I–V) characteristics demonstrate the device's SET and RESET processes. (d) Retention performance of the device in ON and OFF states at a reading voltage of 0.2 V, showcasing stable operation. (e) Negative sweep I–V characteristics highlight distinct conduction regions. Reproduced with permission [188]. Copyright 2020 Wiley‐VCH Verlag GmbH & Co. KGaA, Weinheim.

Borophene‐based memory devices have demonstrated promising results in volatile and non‐volatile data storage applications. For instance, the synthesis of hydrogenated borophene was shown to enhance its stability and electronic properties significantly, enabling its use in memory devices with a high ON/OFF current ratio of 3×10^3^ and low operating voltage of less than 0.35 V (Figure 10) [188]. Such devices exhibit excellent cycling stability and long retention times, which are crucial for practical data storage applications. In addition, the anisotropic memristive behavior of multilayer borophene nanosheets has been explored, revealing distinct volatile and non‐volatile memory functionalities depending on the orientation. This dual behavior simplifies circuit design by allowing dynamic resistive switching under different conditions [82].

#### 5.2.2 Integration with Hybrid Systems

Borophene‐ZnO heterostructures represent another innovative approach to enhancing memory performance [189]. These hybrid devices exhibited a broad photonic response from ultraviolet to near‐infrared wavelengths, with a switching ratio of 5×10^3^ and long‐term stability over 3600 s. This integration leverages the synergistic effects of borophene and ZnO quantum dots, enabling low‐power, high‐density data storage with additional photosensitive functionalities.

#### 5.2.3 Quantum Confinement and Charge Storage

The quantum confinement effects of borophene further enhance its suitability for information storage. Nonvolatile memory devices fabricated using borophene nanosheets mixed with polyvinylpyrrolidone exhibited stable, rewritable memory characteristics. Due to strong quantum confinement effects, these devices showed an increased bandgap to 2.52 eV, ensuring reliable charge trapping and de‐trapping behavior [190].

#### 5.2.4 Comparison with Other 2D Materials

Compared to other 2D materials like graphene and MoS_2_, borophene offers superior performance in specific aspects. For example, while MoS_2_‐based devices demonstrated excellent bistable switching behavior due to charge trapping in polymer matrices [191], the higher electron mobility and structural anisotropy of borophene enhanceed flexibility for multi‐level storage and neuromorphic computing [82]. Moreover, borophene‐based devices can operate at lower voltages, making them more energy‐efficient than traditional resistive memory systems [192, 193].

#### 5.2.5 Challenges and Future Prospects in Information Storage

Despite its potential, the practical deployment of borophene in information storage faces several challenges. These include its sensitivity to oxidation, scalability of high‐quality synthesis, and integration with existing electronic platforms. To overcome these limitations, future efforts should focus on surface functionalization and hybridization strategies. Advancements in synthesis techniques, such as chemical vapor deposition and liquid‐phase exfoliation, and other emerging techniques are expected to play a pivotal role in achieving large‐scale production. In summary, borophene stands out as a revolutionary material for information storage, offering unique capabilities that outperform many existing 2D materials. Its successful integration into memory architectures could redefine the landscape of data storage technologies, bridging the gap between high performance and energy efficiency.

### 5.3 Energy Storage

Two‐dimensional borophene has emerged as a groundbreaking material in energy storage due to its unique properties, such as high electronic conductivity, large surface area, and structural versatility. Its ability to form stable composites and heterostructures has enabled significant advancements in applications like supercapacitors and batteries, addressing the growing global demand for efficient and scalable energy storage solutions.

This table compares 2D materials based on properties critical for energy storage applications, such as theoretical capacity, charge/discharge rates, and durability (Table 5).

**TABLE 5:** 2D Materials for Energy Storage.

Material	Theoretical capacity (mAh/g)	Charge/discharge rate	Electron mobility (cm^2^/V·s)	Mechanical Ss(GPa)	Durability (cycles)	Reference
Graphene	∼372	High	∼15 000	∼130	>1000	[194, 195]
MXenes (Ti_3_C_2_)	∼447	High	∼10 000	∼25–30	>500	[196]
MoS_2_ (TMDC)	∼900	Moderate	∼100–200	∼20–30	∼500	[197]
Black Phosphorus	∼2596	High	∼1000	∼0.5–1	<100	[198, 199]
Borophene	∼1000–2000	Very High	∼10 000–20 000	∼150	>1000 (predicted)	[200, 201]

**FIGURE 11:**
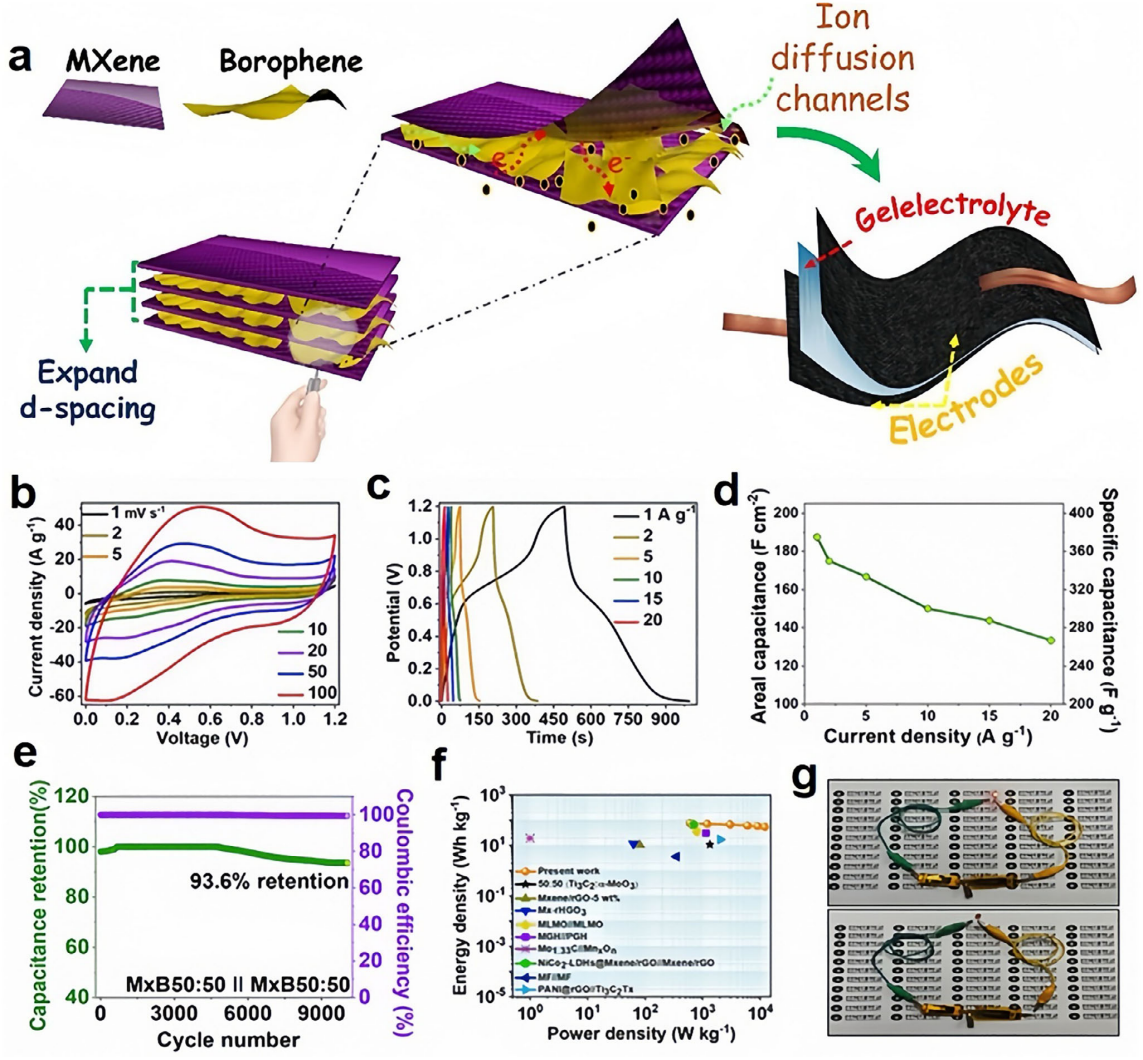
(a) The schematic illustration of the MXene/borophene hybrid structure with expanded interlayer spacing facilitates ion diffusion for supercapacitor applications. (b) CV profiles of the symmetric cell over a potential range of 0–1.2 V at various scan rates. (c) GCD curves at different current densities. (d) Specific and areal capacitance as functions of current density. (e) Cycling stability of the cell at 10 A·g^−^^1^, demonstrating 93.6% capacitance retention after 9000 cycles. (f) Ragone plot comparing energy and power densities of the symmetric/asymmetric cell with other reported configurations. (g) Two MxB 50:50//MxB 50:50 cells in series powering a red LED. Reproduced with permission [202]. Copyright 2023 Published by Elsevier B.V.

Borophene‐based materials exhibit exceptional performance across various energy storage platforms. Flexible MXene/borophene heterostructures achieved a gravimetric capacitance of 626.7 F/g at 1 A/g and an energy density of 75.6 Wh/kg in aqueous electrolytes, demonstrating superior electrochemical behavior (Figure 11) [202]. Integration with nitrogen, phosphorus, sulfur, and fluorine‐doped carbon nanotubes resulted in a specific capacitance of 837 F/g and an energy density of 78.28 Wh/kg, with remarkable cycling stability of 92.72% retention over 10 000 cycles [203]. Sulfur and iron doping of β_12_‐borophene via a microwave‐assisted method enhanced specific capacitances to 202 F/g and 120 F/g, respectively, at 0.25 A/g, showcasing the potential of elemental doping [61]. Stacked borophene‐based electric double‐layer supercapacitors demonstrated an areal capacitance of 417.3 mF/cm^2^ and retained 89.3% of their performance after 5000 cycles [204]. Few‐layer boron sheets produced via scalable liquid‐phase exfoliation exhibited an energy density of 46.1 Wh/kg and retained 88.7% of their initial capacitance after 6000 cycles [104]. Borophene‐graphene composite hydrogels, synthesized through microwave‐assisted methods, achieved a specific capacitance of 455.1 F/g and energy density of 36.77 Wh/kg, with excellent flexibility and 80.8% capacitance retention over 20 000 cycles [205].

Borophene has addressed critical challenges in lithium‐sulfur batteries, such as the polysulfide shuttle effect and poor redox kinetics. Few‐layer β_12_‐borophene synthesized via liquid‐phase exfoliation showed remarkable promise, achieving an areal capacity of 5.2 mAh cm^−^^2^ at a high sulfur loading of 7.8 mg cm^−^^2^. With an ultralow capacity fading rate of 0.039% over 1000 cycles, borophene outperformed many existing materials thanks to its high lithium‐ion mobility and strong polysulfide binding energy [206]. Hydrogenated borophene nanosheets integrated into quasi‐solid‐state electrolytes for lithium‐metal batteries reduced interface impedance, and enhanced ionic conductivity, improved cyclic performance and power density was witnessed [207]. Moreover, borophene nanosheets obtained through the CVD approach achieved a specific capacitance of 350 F/g, surpassing other 2D materials like graphene [77].

In conclusion, borophene‐based materials represent a new frontier in energy storage technologies. Their unique structural, electronic, and mechanical properties and scalable production techniques offer tremendous potential for next‐generation supercapacitors, batteries, and other energy storage devices. Continued innovation in material synthesis and composite design will further bolster its role in advancing global energy solutions.

### 5.4 Catalysis

Borophene with exceptional electronic conductivity and a high density of active sites, has emerged as a revolutionary platform for catalytic applications. Its unique properties, including strong metal‐support interactions and a large surface area, enable significant advancements in hydrogen evolution reactions (HER), oxygen reduction reaction (ORR), oxygen evolution reactions (OER), ethanol oxidation, and noble metal catalyst stabilization.

This table focuses on electrocatalytic properties like hydrogen evolution reaction (HER) and oxygen evolution reaction (OER) overpotentials, key for catalysis applications (Table 6).

**TABLE 6:** 2D Materials for Catalysis.

Material	HER overpotential (mV)	OER overpotential (mV)	Active sites per cm^2^	Surface area‐to‐mass ratio (m^2^/g)	Tunability	Reference
Graphene	∼200–300	∼350–450	Moderate	∼2600	Low	[208]
TMDCs (MoS_2_)	∼100–200	∼300–400	High	∼100–500	Moderate	[209]
h‐BN	∼500–600	∼400–500	Low	∼200–300	Low	[210, 211]
PtSe_2_	∼50–100	∼250–300	High	∼100–200	High	[212]
PdTe_2_	∼70–120	∼270–320	High	∼100–200	High	[213]
Borophene	∼50–100 (predicted)	∼200–300 (predicted)	Very High	∼2000–3000	High	[214, 215]

In HER, borophene‐supported catalysts have demonstrated outstanding performance. When paired with Rh nanoparticles, borophene nanosheets achieved an overpotential as low as 66 mV in acidic media and 101 mV in alkaline media at a current density of 10 mA/cm^2^. These results are comparable to platinum‐based catalysts, which are the industry benchmark but have enhanced durability across different pH conditions. In addition, borophene nanosheets grown via CVD showed a Tafel slope of 69 mV/dec, highlighting their efficient charge transfer capabilities during HER [216, 217].

**FIGURE 12:**
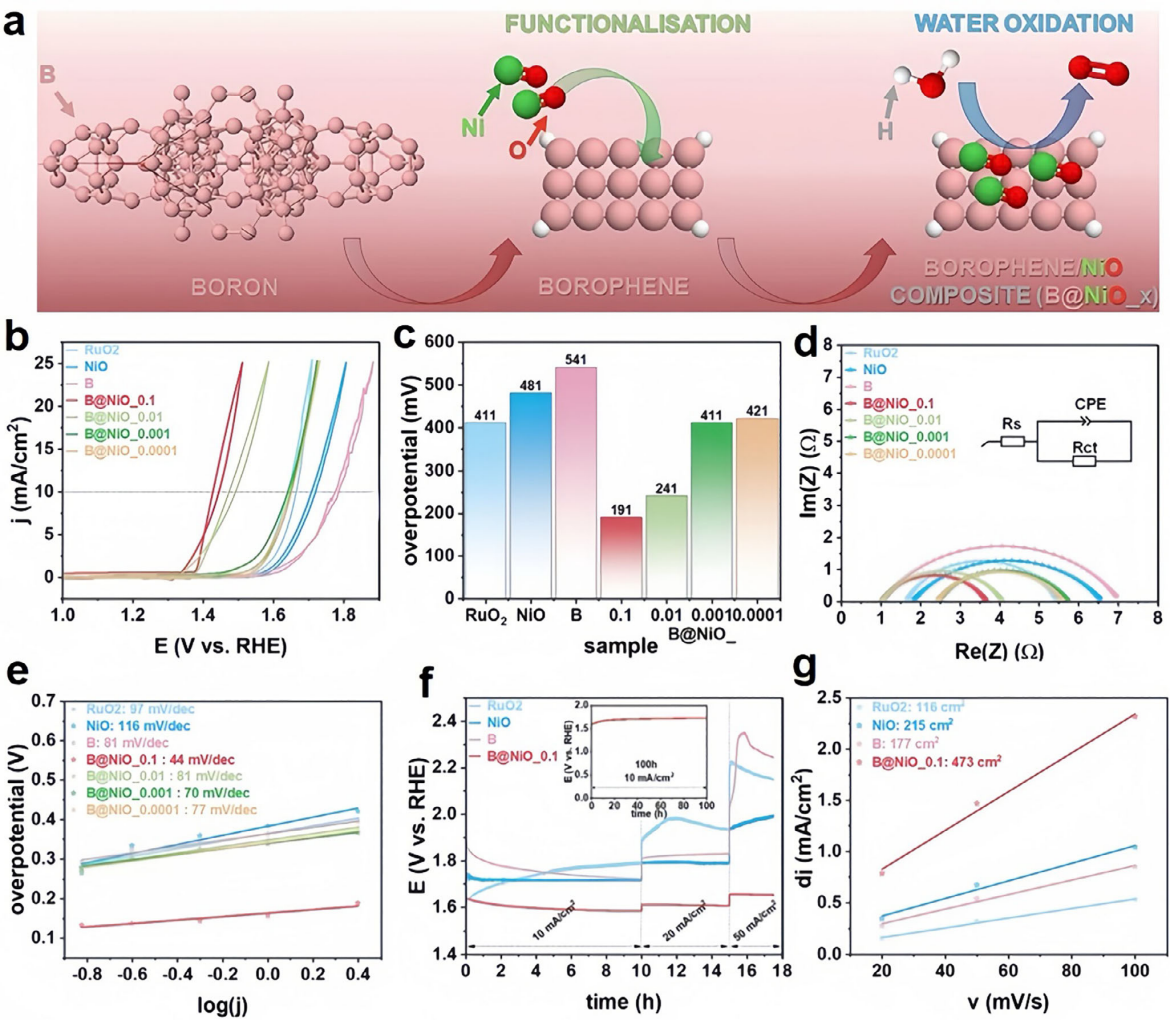
(a) Schematic illustration of borophene functionalization with NiO for enhanced water oxidation. (b) Polarization curves, (c) overpotential comparison, (d) electrochemical impedance spectra, (e) Tafel plots, (f) stability tests with inset showing 100‐h durability, and (g) capacitive current measurements demonstrating electrochemical active surface areas for RuO_2_, NiO, borophene, and B@NiO_x composites. Reproduced under the terms of the Creative Commons CC‐BY license [218]. Copyright 2023 The Author(s). Published by Elsevier B.V.

For OER, the synergy of borophene with transition metal oxides has delivered remarkable results. A borophene/nickel oxide (NiO) composite achieved an exceptionally low overpotential of 191 mV at 10 mA/cm^2^ and a Tafel slope of 44 mV/dec, outperforming many traditional catalysts like IrO_2_ and RuO_2_ (Figure 12) [218]. Similarly, borophene integrated with cobalt oxide (Co_3_O_4_) reduced the overpotential to 270 mV with a Tafel slope of 62 mV/dec, showcasing its potential for efficient water splitting and clean energy applications [219].

In ethanol oxidation reactions (EOR), borophene has demonstrated significant potential as a catalyst support. A PdNiO nanoflower (PdNiONF)‐borophene composite showed superior catalytic activity, achieving a current density of 0.12 mA in alkaline conditions. This catalyst also displayed remarkable resistance to poisoning by intermediate oxidation species, making it ideal for fuel cell applications. The role of borophene in enhancing the interaction between Pd and ethanol molecules contributes to its outstanding performance compared to conventional Pd/C catalysts [79, 220].

Furthermore, hydrogenated borophene has enabled the synthesis of advanced noble metal catalysts with ultrafine particle sizes and high dispersion. For example, Pt nanoparticles (∼2.5 nm) supported on hydrogenated borophene demonstrated superior stability and activity in ORR, with a high loading of 80 wt.% Pt. This strong metal‐support interaction significantly enhances the durability of these catalysts under harsh operating conditions, making them more viable for industrial applications [108, 221]. The versatility and transformative properties of borophene make it a leading material for next‐generation catalysts. By enabling efficient, stable, and cost‐effective energy conversion and storage solutions, borophene catalysis is set to drive advancements in hydrogen production, fuel cells, and beyond. Therefore, extensive research on introducing more functionalization on borophene and its composites with other 2D or porous nanostructures is highly necessary as it will further unlock its potential across various catalytic domains.

In summary, borophene is intrinsically metallic, exhibiting no bandgap, very high electrical conductivity, and strong directional (anisotropic) charge transport. Its properties are highly dependent on atomic structure due to the existence of multiple polymorphs. Consequently, borophene is well suited for applications such as nanoscale interconnects, transparent conductive electrodes, metallic contacts in 2D heterostructures, and plasmonic devices. In contrast, some other Xenes are metallic but largely isotropic, making their electronic properties harder to tune. Transition metal dichalcogenides (MoS_2_, WS_2_) are semiconducting and therefore preferred for transistor applications, while hBN is insulating and commonly used as a dielectric layer. Borophene exhibits a strong, tunable plasmonic response in the infrared range with direction‐dependent optical behavior, supporting broadband anisotropic plasmons. This makes it attractive for plasmonic waveguides, sensors, and nano‐antennas, with better plasmon tunability than graphene. Unlike borophene, TMDCs primarily show excitonic optics rather than plasmonics. Mechanically, borophene is stronger than graphene along certain directions and displays extreme flexibility, stretchability, and even auxetic behavior in some phases. These anisotropic mechanical properties enable applications in flexible electronics, lightweight composites, and NEMS. Furthermore, borophene has a highly reactive, buckled surface with abundant active sites, strong metal‐ion binding, and high theoretical battery capacity. It is promising for energy storage, hydrogen storage, electrocatalysis, and gas sensing. Overall, borophene uniquely combines metallicity with high chemical activity, a rare characteristic among 2D materials.

## 6 Challenges of Borophene and the Way Forward

### 6.1 Limitations of Borophene

Despite its promising properties, borophene faces several challenges that must be addressed before it can be widely adopted for commercial applications. These limitations pertain primarily to the non‐scalability of production, environmental instability, unsuitable electronic properties, and device integration issues, specially work function mismatch and lack of interaction between boron and metals. It is highly crucial to address these certain issues in order to unlocking the potential of borophene in various fields including electronics, energy storage, and catalysis. This section discusses the key challenges and barriers to the commercialization of borophene.

#### 6.1.1 Scalability of Production

One of the primary obstacles to the commercialization of borophene is the difficulty in achieving large‐scale and uniform production with phase purity. The current synthesis methods, including MBE, CVD, and ALD, often require high‐vacuum conditions, specialized substrates, and sophisticated equipment, which limit the scalability of borophene production [73, 222]. In addition, the growth of borophene is typically substrate‐dependent, with substrates such as silver and gold necessary to stabilize borophene lattice on the substrate, as their FCC lattice can support metal‐induced crystallization at relatively low crystallization temperature [223]. Moreover, post‐deposition crystallization by laser or an electric field can be employed [224]. This requirement significantly increases the cost of production for crystalline borophene and complicates its transfer onto other substrates for device fabrication. Therefore, developing scalable and cost‐effective synthesis methods, such as bottom‐up solution‐based growth techniques using metal for growth catalysts and surfactants (such as CTAB) [103] or modified CVD processes such as microwave plasma CVD [225] will be crucial for enabling the widespread use of borophene. Protocols must be developed for extracting boron, its purification, and crystallization to achieve crystalline powder, which can then be used for the top‐down synthesis of borophene. Further, novel bottom‐up synthetic approaches must be developed for mass‐scale production from lab reagents such as boric acid, sodium borohydride, etc.

#### 6.1.2 Oxidation Woes

Borophene is highly reactive and prone to oxidation when exposed to ambient conditions, significantly affecting its electronic, optical, and mechanical properties. Atomistically controlled studies have been carried out to investigate experimental surface oxidation of borophene [226]. First principles studies have been carried out to understand the oxidation of borophene [227]. Oxidation rate as per TERS line profile was found to be 0.15–0.2 nm/min. It should be noted that borophene oxide has band gap of >3.3 eV and marginal electron concentration, showing significantly large electronic scattering which results in lowered electronic mobility for thicker oxide layer. Also, in ambient air (humid), hydroxide formation is imminent. The hydroxide layer can transform to oxide upon heating. Diffusion of oxygen atoms in the interiors degrades electronic character of borophene. Moreover, hydroxyl group attachment can weaken the crystal mechanically. Thus, structural fidelity and chemical character would drastically change upon oxidation/hydroxidation. Therefore, it is crucial to prevent the borophene layers in device from chemical degrading. This instability presents a significant challenge for practical applications, as maintaining the properties of borophene in real‐world environments is challenging. Various strategies have been proposed to address this issue, including encapsulation with protective layers (e.g., alumina, [228, 229] hexagonal boron nitride or graphene), surface functionalization [230], non‐covalent functionalization [231] or passivation of the reactive sites through functionalization. However, these methods can complicate fabrication and alter the intrinsic properties of borophene. Therefore, it is highly crucial to develop effective oxidation‐resistant strategies while preserving the unique characteristics of borophene because it is essential for its commercial viability, particularly for applications in electronics and catalysis, where stability is critical.

#### 6.1.3 Lack of Band Gap

Unlike semiconducting 2D materials such as transition metal dichalcogenides (TMDCs), borophene exhibits metallic behavior, which limits applications in field‐effect transistors (FETs) and other electronic devices that require a band gap. The absence of an intrinsic band gap restricts the application of borophene in digital electronics, where on‐off switching is necessary for device operation. It should be noted that the ON/OFF ratio is proportional to the band gap. Various efforts have been made to introduce a band gap in borophene through doping [61], surface functionalization [230], 2D‐2D hybridization [28], heterostructure formation [32], or strain engineering methods. While some success has been achieved in tuning the electronic properties, these approaches often result in trade‐offs with other properties, such as carrier mobility or mechanical flexibility. For example, edge doping and vacancies/defects generated during doping reactions enhance electronic scattering. Moreover, hydrogenation of borophene can alter electronic, thermal, optical, and mechanical properties. Borophene, borophene, borides and other boron containing materials exhibit remarkable structure‐stoichiometry‐properties relationship as shown in the following table (Table 7).

**TABLE 7:** Comparison of structures, stoichiometry and key properties of borophene with borophane and borides.

Material	Structure	Stoichiometry	Key Properties
Borophene	• 2D atom‐thin sheet of boron • Polymorphic: various lattice geometries (β12, χ3, etc.) • Anisotropic, corrugated or planar depending on phase	• Elemental boron • Stoichiometry varies with vacancy pattern rather than chemical composition	• High electrical conductivity (metallic) • High mechanical strength and flexibility • Anisotropic thermal and electronic behavior • High surface reactivity; useful for sensors, batteries, catalysis
Borophane	• Fully or partially hydrogenated borophene • Hydrogen atoms bonded to boron sheet, stabilizing it • More planar and chemically stable than borophene	• B–H composition • Common representation: B:H ≈ 1:1 for fully hydrogenated forms	• Increased stability vs. borophene • Can be semiconducting or metallic depending on hydrogenation pattern • Lower surface reactivity • Potential uses in flexible electronics, hydrogen storage
Borides	• Crystalline compounds of boron + metals (e.g., TiB_2_, MgB_2_, ZrB_2_) • Structures include hexagonal, tetragonal, orthorhombic depending on metal and phase	• Metal–boron compounds • Stoichiometries include MB, MB_2_, MB_4_, M_2_B, etc. • Highly variable depending on metal	• Extremely high hardness (often superhard) • High melting points and chemical resistance • Some are excellent conductors (e.g., MgB_2_ is superconducting) • Used in armor, cutting tools, coatings, and high‐temperature materials

Therefore, extensive research and development on modulating the electronic properties of the borophene without compromising its other advantageous characteristics is highly needed.

#### 6.1.4 Insufficient Carrier Concentration

To ensure efficient charge transport, materials must possess a high carrier concentration for many electronic and optoelectronic applications. Although borophene exhibits high electronic mobility, its carrier concentration may not be sufficient for specific applications, such as high‐frequency transistors or infrared photodetectors. This limitation arises from its inherent electronic structure, which can be challenging to modify without compromising other properties. Techniques such as defect engineering [232], doping with transition metals [61], and hybridization [28] have been explored to increase carrier concentration. However, balancing high carrier density and maintaining other desirable properties remains a significant challenge. Moreover, doping above a specific limit gives rise to lattice distortion or amorphization. Strong reagents/solvents can rupture or fragment borophene sheets. Enhancing carrier concentration in the borophene without adversely affecting stability, conductivity, or mechanical strength will be crucial for its use in advanced electronic devices.

#### 6.1.5 Phase Selection Issues

Borophene exists in polymorphs, such as β_12_, χ_3_, α and hexagonal structures. For example, β_12_ and χ_3_ phases are metallic whereas α phase is semiconducting. Their physical (electronic, optical, thermal, thermoelectric, magnetic, etc.) and chemical properties (chemical reactivity with oxygen, acids, bases, and catalytic character) are distinct [73, 233, 234] While this polymorphism provides opportunities for tuning the properties of borophene for specific applications, it also introduces a significant challenge in controlling the phase selection during synthesis. Achieving uniform phase distribution across large‐scale samples is complex, and mixed‐phase borophene can exhibit unpredictable or suboptimal properties. Addressing this issue requires improved understanding and control of the growth conditions that govern phase formation, such as substrate choice, temperature, and deposition rate. Liquid phase top‐down synthesized borophene phase can be filtered using different centrifugation speeds. Further, appropriate catalysts can be used during bottom‐up synthesis approaches to grow the specific borophene phase preferentially. Advances in phase engineering techniques could enable the targeted synthesis of specific borophene polymorphs, optimizing their properties for applications ranging from energy storage to quantum devices.

#### 6.1.6 Device Integration Issues

Integrating borophene into existing device architectures poses challenges due to the requirements for stability. The reactivity and environmental instability of borophene necessitate encapsulation or passivation to maintain performance, which adds complexity to the fabrication process. Moreover, the lack of well‐established protocols for borophene transfer, patterning, and deposition makes incorporating the material into conventional semiconductor processing workflows challenging. Unlike graphene or TMDCs, which have undergone significant development in device fabrication techniques, borophene‐based device integration is still in its early stages.

**FIGURE 13:**
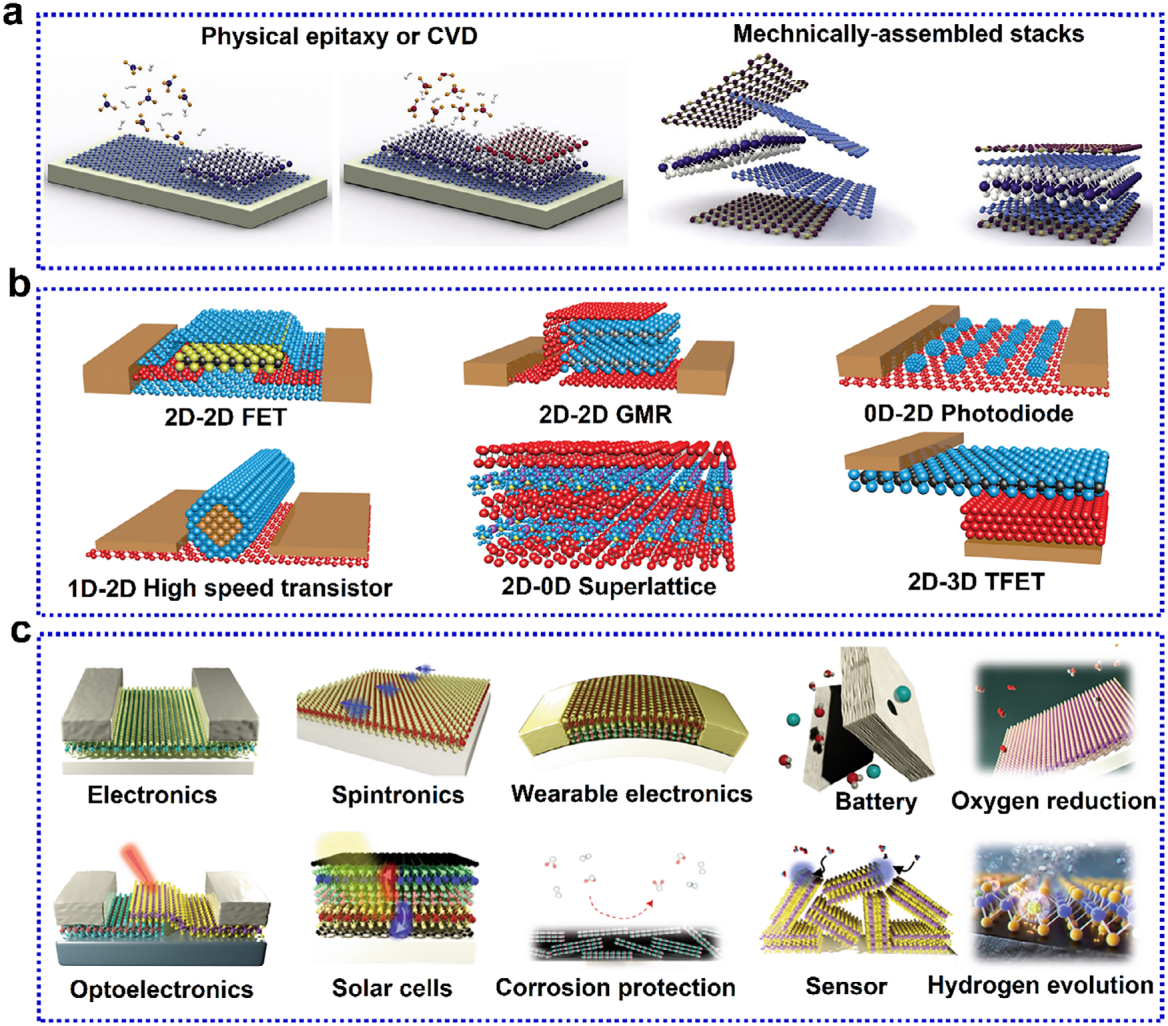
(a) Fabrication of van der Waal heterolayer. Reproduced with permission [235]. Copyright 2016, American Association for the Advancement of Science. (b) various hybrid structures. Reproduced with permission [238].Reproduced under the terms of the CC‐BY Creative Commons Attribution 4.0 International license (https://creativecommons.org/licenses/by/4.0).[217] Copyright 2019, Springer Nature Limited. and (c) potential device applications. [239]. Copyright 2022, The Author(s).

As borophene has high electronic mobility and thermal conductivity, it can be employed as a component layer in hetero‐layered stacks for functional devices in electronics, optoelectronics, thermoelectric, and several others. However, complications in stacking several layers remain a challenge. For example, achieving a particular sequence of layers and retaining the flatness of sheets (without crumpling/tearing, etc.) is difficult. There are presently two potential ways to achieve stacking. One via physical epitaxy or CVD and another via mechanically assembled stacks (Figure 13a) [235]. The physical epitaxy/CVD method can grow desirable 2D materials on a selected substrate area sequentially and then go for lithography steps to constitute devices, followed by wire bonding to achieve devices [98, 236, 237] Chemically exfoliated borophene can be stacked using mechanical assembly under dilute concentration conditions. Such methods are widely used for 2D materials [63]. Layer‐by‐layer transfer of borophene with companion layers can be achieved similar to those reported for graphene and boron nitride [62]. Integration of borophene with 0D, 1D, 2D, and 3D quantum materials can result in various devices and sensors, including highly functional electronic chips (FETs), spintronic chips with giant magnetoresistance (GMR), excitonic devices such as photodiodes, etc. (Figure 13b) [238]. Efficient transfer/stacking, atomistic vertical integration, and electrode fabrication can be helpful in various borophene‐based devices (Figure 13c) [239]. Strategies developed and implemented for graphene can be beneficial for borophene as well. For example, layer‐by‐layer transfer employing a dry transfer technique using PDMS or sequential wet chemical transfer using aqueous FeCl_3_ solutions for dissolving copper substrate, followed by laser shock integration, can be used [62]. Moreover, work function mismatch and non‐interacting component layers in the device are the problems that need immediate attention. Epitaxial growth of borophene can help to address such issues. In addition, the fabrication of sandwich layers to enhance integration can assist. In particular, the lack of low‐temperature direct borophene growth protocols is a barrier to the direct imprinting of borophene circuits on flexible substrates. Laser‐based 3D printing using computer‐controlled methods can be helpful in this regard. Further, laser shock printing can be developed for borophene.

#### 6.1.7 Reactivity Problem in Catalysis

While the high surface reactivity of borophene is advantageous for catalytic applications, it also poses challenges in maintaining stability during catalytic reactions. The active sites of borophene can become passivated or degraded over time, reducing catalytic efficiency. The high reactivity can also lead to side reactions that negatively impact selectivity in specific catalytic processes. Strategies such as defect generation [157], doping [61], surface functionalization [230], hybridization with other materials [28], and functionalization with stabilizing agents can enhance the catalytic performance of borophene while mitigating reactivity issues. However, since it is reactive under acidic and alkaline environments, these modifications must be carefully optimized to avoid compromising the intrinsic catalytic properties or increasing fabrication complexity. Since doping can alter reactivity towards chemical reagents, choosing a dopant metal to equip borophene for a particular catalysis reaction will be crucial. Similarly, compatible companion layers can be selected for hybridizing borophene for specific catalytic pursuits.

### 6.2 Future Synthetic Approaches

Innovative synthesis techniques are being explored to overcome the challenges of borophene production and enable its commercialization. These future synthetic approaches aim to improve scalability, control over phase selection, and the stability of borophene. The following strategies, including novel exfoliation methods and bottom‐up growth techniques, could offer solutions to existing limitations by providing greater control over the structure and properties of borophene.

#### 6.2.1 Intercalation Exfoliation

Intercalation exfoliation involves inserting foreign atoms or molecules between the layers of boron in a boron crystal to weaken interlayer interactions and facilitate the exfoliation of monolayer borophene. This method has been widely used to produce other 2D materials, such as graphene and TMDCs [240, 241, 242, 243]. In the case of borophene, intercalation agents such as alkali metals or organic molecules can disrupt the interatomic forces within layered boron compounds, leading to the delamination of borophene sheets. This technique offers the potential for scalable production of borophene with controlled thickness. However, selecting appropriate intercalation agents is crucial to avoid excessive reactivity or degradation of borophene during exfoliation. Advances in intercalation chemistry and a better understanding of the interaction between boron layers and intercalants are essential to optimize this approach for large‐scale borophene production.

#### 6.2.2 Cryo‐Exfoliation

Cryo‐exfoliation is a novel method that involves freezing layered boron compounds and then mechanically or chemically breaking them apart at cryogenic temperatures [244, 245, 246, 247]. Low temperatures can help preserve the structural integrity of borophene by minimizing thermal degradation or oxidation during exfoliation. In addition, cryo‐exfoliation can produce defect‐free borophene sheets with minimal contamination, making it suitable for high‐quality applications, such as electronic devices and quantum materials. Recent cryogenic techniques and equipment advancements have made cryo‐exfoliation more accessible, providing a promising route for the scalable synthesis of high‐purity borophene. Further research is needed to optimize cryogenic conditions, such as temperature and freezing time, to enhance the exfoliation efficiency and control the morphology of the resulting borophene sheets.

#### 6.2.3 Photo Exfoliation

Photo exfoliation has recently been utilized to give rise to light‐induced chemical reactions to peel off layers of van der Waal materials, allowing for the formation of atomic sheets. In this approach, laser‐induced heating expands bulk crystals out‐of‐plane, giving rise to the intercalation of solvents. The interaction of intercalants with atom sheets in the interior of bulk crystals weaken inter‐layer coupling, giving rise to exfoliation [19]. The advantage of photo exfoliation is that it can be a controllable and non‐destructive method for producing borophene, as the light exposure can be precisely regulated to achieve uniform thickness and phase purity. One of the major advantages of this technique is that different wavelengths of light can be employed to selectively target specific atomic bonds, providing extra control over the exfoliation process. However, finding the optimal light sources, intensities, and reaction environments is a critical challenge that must be addressed to maximize efficiency and prevent damage to the material. Ongoing research into photochemical reactions and photon‐material interactions will likely advance the development of photo exfoliation techniques for borophene.

#### 6.2.4 Bottom‐Up Liquid‐Phase Metal‐Catalyzed Crystal Growth

Bottom‐up liquid‐phase synthesis is an emerging method for producing borophene. Boron precursors are used in liquid phase processing and give rise to 2D layer growth surfactants (such as CTAB) have been utilized [103]. Metal atoms such as copper or silver can act as nucleation sites, promoting the formation of borophene on their surfaces. This approach can yield high‐quality borophene with well‐defined structures and minimal defects, as the growth would occur in a controlled liquid environment. Also, liquid‐phase synthesis can be scaled up by optimizing the reaction parameters, such as temperature, concentration, and stirring rate. Metal‐catalyzed crystal growth will also allow the possibility of producing borophene with specific polymorphs by carefully choosing the metal catalysts and liquid solvents. Challenges associated with this method include the removal of residual metal contaminants and the stabilization of borophene during transfer to other substrates. Further work refining the synthesis conditions and understanding the mechanisms of liquid‐phase growth will be essential to advance this approach.

#### 6.2.5 Topotactic Transformation

Topotactic transformation involves converting a precursor material into borophene through a solid‐state reaction, wherein the original crystal structure is partially retained and transformed into the desired borophene structure. This method relies on the selective removal or insertion of atoms within a parent compound to form borophene without entirely disrupting the crystalline framework. For instance, boron‐rich ceramics or layered borides could be subjected to chemical treatments that extract certain atoms, allowing borophene layers to form in situ [248, 249]. Topotactic transformation offers a promising pathway for synthesizing borophene with tunable properties, as different precursor materials and reaction conditions can be employed to control the final structure. Additionally, this approach may enable the production of borophene directly on device‐compatible substrates, simplifying the integration process for practical applications. The main challenge in topotactic transformation is the precise control over the reaction to ensure uniform and high‐quality borophene formation, which requires further research into reaction kinetics and precursor material selection.

**FIGURE 14:**
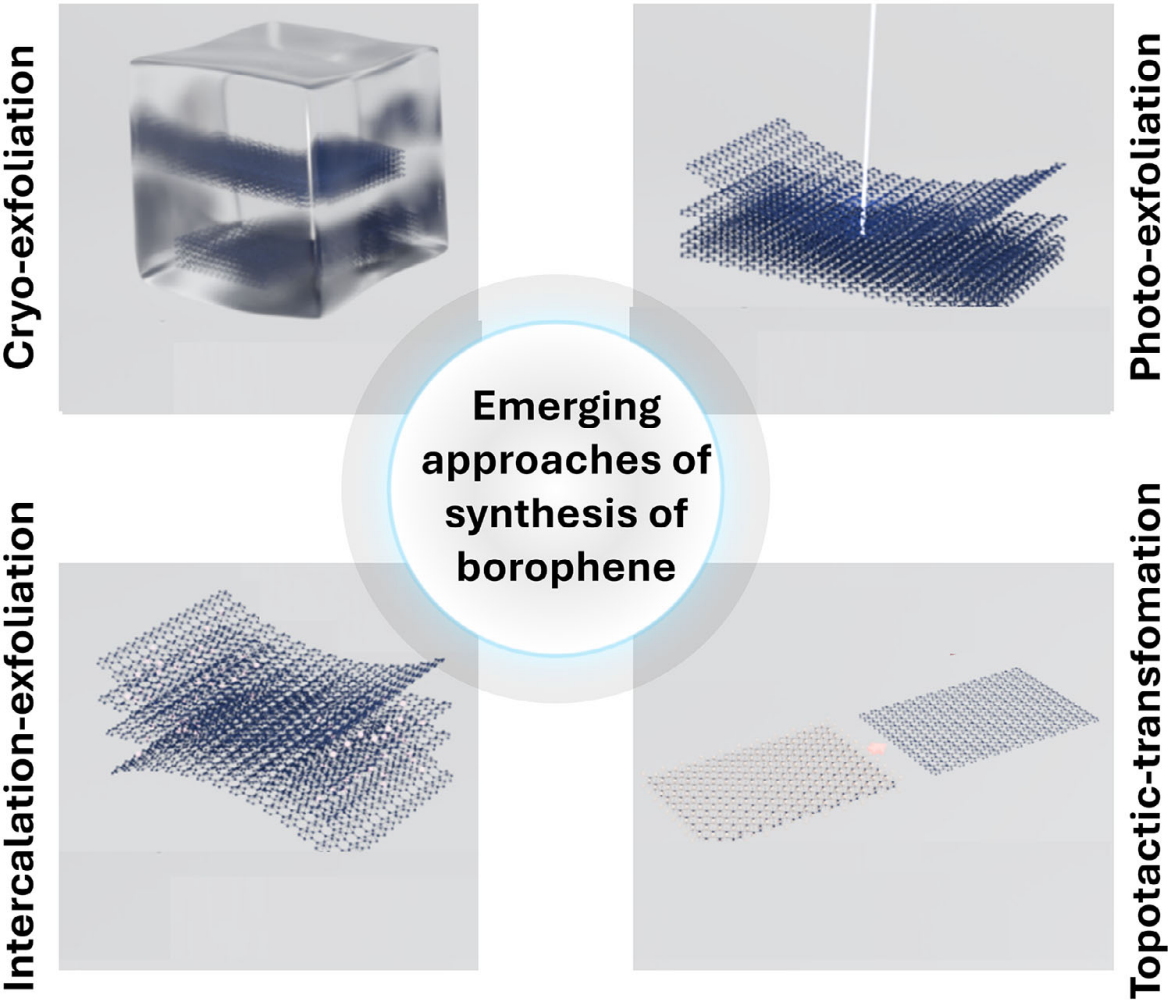
Emerging approaches of synthesis of borophene.

These advanced futuristic approaches (Figure 14) for borophene synthesis can be employed to achieve a scalable synthesis of borophene with chemical phase purity, which will catapult it toward commercialization and frontline applications.

**FIGURE 15:**
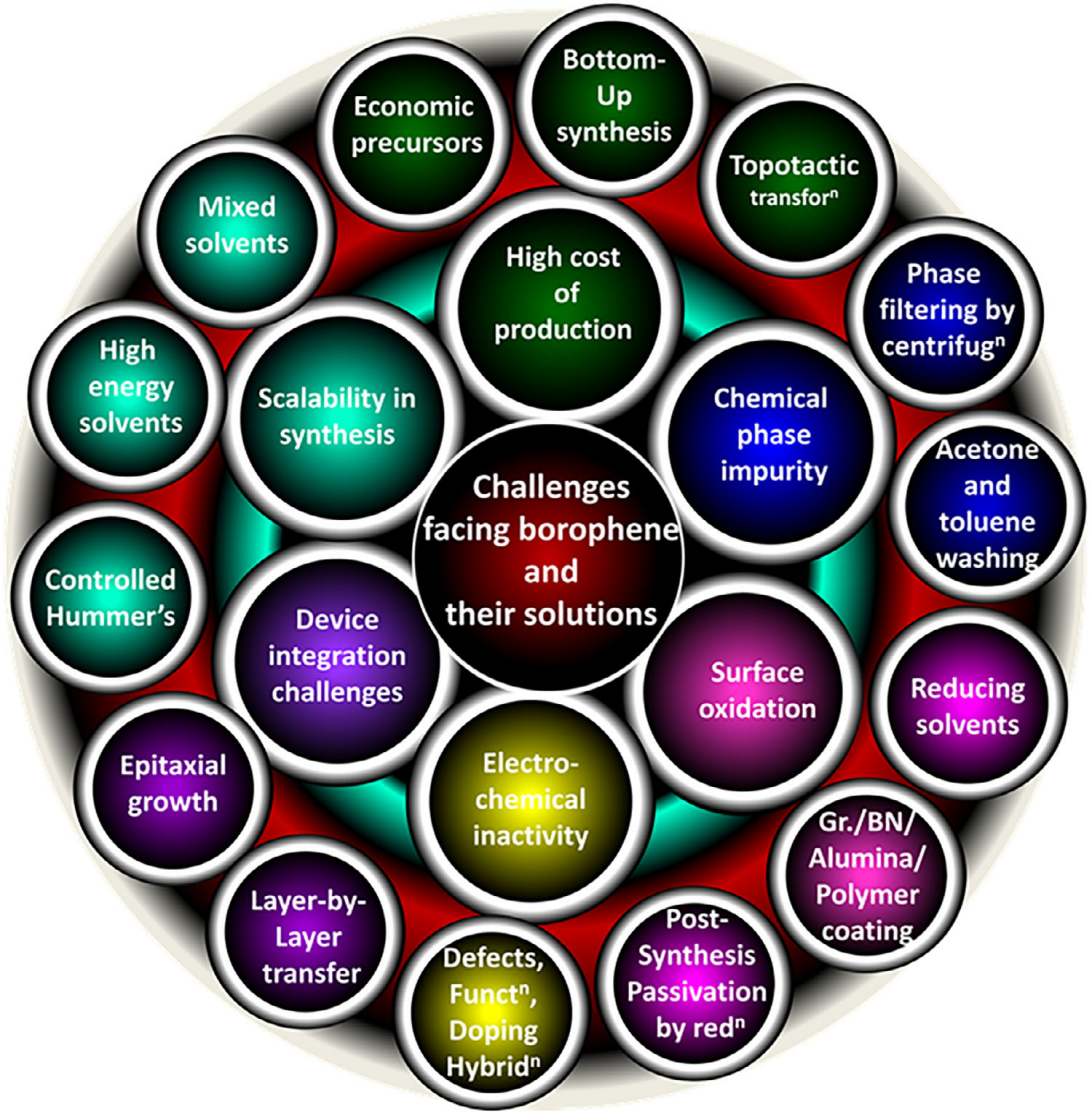
Key challenges borophene is facing and plausible solutions.

In summary, while addressing synthetic challenges of scalability in synthesis via adopting synthetic strategies (such as cryoexfoliation, intercalation exfoliation and topotactic transformation etc) can yield large scale production of borophene, novel bottom up liquid‐phase synthesis will take it to next level, surface oxidation issues can be addressed by lamination of borophene with graphene, BN and alumina, or sandwitching it in heterolayered ddevices (Figure 15). Further, nanoarchitectonics by doping, surface functionalization, hybridization and defect engineering can not only enhance local electron density, generated catalytic sites can help enhance catalytic or electrochemical performances (Figure 15). Similarly, device integration challenges can be resolved by layer‐by‐layer self‐assembly of borophene, and by annealing the device or by laser shock integration (Figure 15).

### 6.3 Future Applications of Borophene

Borophene, with its exceptional properties and versatile characteristics, holds great promise for advancing a variety of next‐generation technologies. The unique combination of high electrical conductivity, flexibility, reactivity, and tunable electronic properties suggests that borophene could play a key role in several futuristic applications [134, 250, 251]. This section explores potential directions for using borophene across diverse fields, including spintronics, twistronics, photonics, energy storage, thermal management, and biomedical applications.

#### 6.3.1 Electronics (FETs)

Depending on the structure, borophene exists in both metallic and semiconducting form. However, the suitable band gap engineering and carrier injections of borophene can render fit for field effect transistor (FET) devices. Experimental advances in this direction have not yet been at the level of FET devices; however, advances in advanced surface and interface engineering will help it embark soon. Theoretical advances have, however, already been in place in this context [141, 252, 253, 254].

#### 6.3.2 Spintronics (Quantum Memory Devices)

Spintronics is an emerging field that exploits the spin of electrons and their charge for information processing and storage. It could potentially lead to highly efficient quantum memory devices. It was predicted that borophene exhibits high spin‐polarization and tunable magnetic properties which make it an ideal candidate for spintronic applications [255, 256, 257]. Ultrafast switching of spin‐FET of borophene‐based devices has been explored [258]. Defects and dopants could be introduced to manipulate the magnetic ordering of borophene, enabling its integration into spintronic devices [259]. Moreover, the low‐dimensional nature of borophene may allow for the creation of ultra‐thin spin filters or spin valves [260], critical components in memory [82], and sensing [261, 262] Although borophene has a lot of possibilities, much needs to be accomplished to optimize parameters and experimental conditions for its synthesis, purification, phase filtering, transfer, and device integration towards borophene‐based spintronic devices.

#### 6.3.3 Twistronics

Twistronics involves manipulating electronic properties in 2D materials by rotating two layers relative to each other, creating moiré patterns that lead to unique superconducting or insulating phases. The flexibility and polymorphic nature provide opportunities to explore Moiré superconductivity when stacked with other 2D materials such as graphene or TMDCs. Borophene bilayers have earlier been foretold to have superconductivity [263, 264]. This potential positions borophene as a critical player in developing future quantum computing devices that rely on robust superconducting states. Even thermoelectric devices are being shaped based on borophene twistronics [265]. Moiré Superconductivity in borophene‐based heterolayers is yet to be experimentally explored.

#### 6.3.4 Photonics

The anisotropic optical properties of borophene, which include strong absorption and high refractive indices, make it highly suitable for photonic applications such as waveguides, optical modulators, and photodetectors. Broadband non‐linear photonics [266] and Giant photonic spin Hall effect [267] have been predicted. Borophene‐based photonic devices could benefit from their tunable electronic structure, allowing for wavelength‐specific optical responses. Integrating borophene with other optoelectronic materials such as MoS_2_ and phosphorenes may make it possible to design multi‐functional photonic circuits for use in communications, imaging, and quantum optics.

#### 6.3.5 Electrodes in LEDs and Solar Cells

The high electrical conductivity and transparency in the thin layers position of borophene make it as a promising candidate for electrodes in light‐emitting diodes (LEDs) and solar cells. Its mechanical flexibility could enable the fabrication of flexible, foldable, and wearable photovoltaic devices, expanding the applicability of solar energy harvesting technologies. The potential of borophene for carrier mobility enhancement may also lead to improved power conversion efficiencies in solar cells. Varieties of phases are photoluminescent themselves in their pristine form [103]. However, heteroatom/transition metal doping, surface functionalization, and hybridization can adequately tailor other metallic and semiconducting phases to render it suitable for p‐/n‐type semiconducting nature and hence useful for excitonic devices (LEDs and solar cells).

#### 6.3.6 Next‐Generation Light, Flexible, and Foldable Batteries

The combination of borophene's large surface area and exceptional conductivity is highly beneficial for energy storage devices, including next‐generation batteries that are light, flexible, and foldable. Borophene could be an advanced electrode material in lithium‐ion, sodium‐ion, or other metal‐ion batteries, providing high capacity, fast charging capabilities, and long cycle life [78, 200, 207, 268, 269, 270]. Furthermore, integrating borophene with polymer‐based electrolytes or current collectors could result in mechanically flexible energy storage devices suitable for wearable electronics. Although several theoretical advances have been published on borophene‐based batteries and supercapacitors, and lab‐scale demonstrations have also been realized, a lot more is needed. Optimization of borophene synthesis, electrode fabrication protocols, moderation (especially adaptability), and implementation are the next steps toward commercialization.

#### 6.3.7 Thermoelectric Applications

Thermoelectric materials can convert temperature gradients into electrical energy, making them useful for power generation and thermal management. Borophene's high electrical and anisotropic thermal conductivity may enable efficient thermoelectric devices [271, 272, 273]. By optimizing its structural and electronic properties through doping or creating hybrid materials, borophene‐based thermoelectric could achieve higher power factors and figure‐of‐merit (ZT) values, contributing to efficient waste heat recovery systems.

#### 6.3.8 Thermoplastics (Thermal Packaging)

The high thermal conductivity and mechanical strength of borophene make it suitable for use as a filler material in thermoplastic composites for thermal packaging applications [75]. Metal intercalation can enhance thermal transport in borophene [151]. Further, doping, functionalization, and hybridization can be used as tools to moderate its prompt local heat removal. Such composites could be used in electronic devices to dissipate heat more effectively, thereby enhancing the performance and lifespan of integrated circuits. Borophene could also be incorporated into thermoplastic films used in flexible electronics, providing mechanical reinforcement and heat management.

#### 6.3.9 Thermal Interfacing (Electronic Cooling)

Borophene has garnered attention for its exceptional thermal conductivity, which is crucial for thermal interfacing in electronic cooling applications. Studies have reported that borophene hydride exhibits high thermal conductivities of approximately 335 W/mK and 293 W/mK along the zigzag and armchair directions [274]. These values are comparable to those of graphene, making borophene a promising candidate for thermal interface materials (TIMs) in microelectronics, LEDs, and high‐performance computing systems. The efficient heat dissipation properties of borophene can enhance the performance and reliability of electronic devices by mitigating heat buildup. Its potential application as a TIM involves bridging heat‐conducting components, thereby facilitating effective cooling. This capability is particularly valuable in microelectronics, where managing thermal loads is critical to device longevity and efficiency. Further research into integrating borophene‐based TIMs in electronic systems is necessary to understand and optimize their performance fully.

#### 6.3.10 As a Substrate for Growth of Heterolayers

The unique surface properties of borophene could make it an effective substrate for the growth of heterolayers of other 2D materials. Its ability to induce strain or alter the electronic band structure of overlying materials could be leveraged to engineer new properties in heterostructures. This approach could lead to the development of novel electronic, optical, or magnetic devices by tuning the interactions at the interface between borophene and other layered materials.

#### 6.3.11 Quantum Computers

The extensive use of 2D materials for electronics provides immense hope for borophene‐based quantum computers [11, 64, 275, 276] The potential superconducting properties of borophene and its compatibility with existing 2D quantum materials make it a promising candidate for quantum computing applications. Its tunable electronic structure and ability to host topological phases could be exploited in designing qubits, the basic units of quantum information processing. Moreover, borophene's long spin coherence length makes it apt to be employed for informatics without much loss. It is also expected that hybridizing borophene with other 2D superconductors may enhance coherence times and improve the stability of quantum states in quantum computing devices.

#### 6.3.12 Disease Diagnosis and Biomedical Applications

**FIGURE 16:**
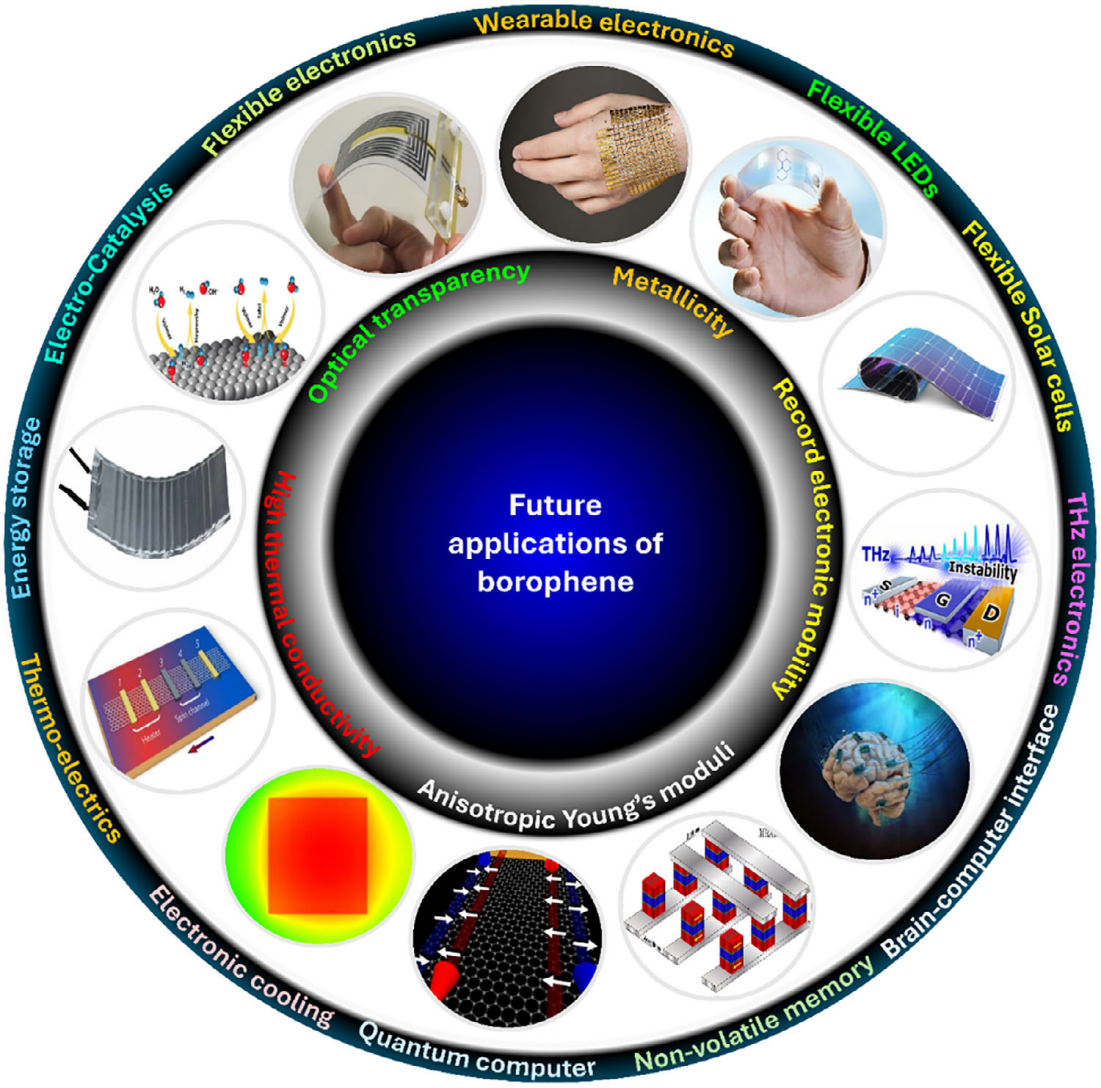
Future applications of borophene.

Borophene's large surface area and potential for biofunctionalization open possibilities in biomedical applications, such as disease diagnosis and drug delivery. Its ability to adsorb biomolecules or ions could be used to develop sensitive biosensors for detecting specific disease markers [277, 278, 279, 280, 281]. Furthermore, functionalizing borophene with biocompatible molecules could enable its use as a drug delivery platform, where the high surface area allows for a high loading capacity of therapeutic agents. The unique combination of record electronic mobility, high thermal conductivity, and high Young's modulus flexibility makes borophene the best choice for ultrafast biosensing material candidates. As optimization of synthesis parameters, purification protocols, and device fabrication adaptable for borophene are still underway, it is expected that borophene‐based futuristic devices and ultrafast sensors may be developed in the coming years (Figure 16).

In conclusion, while borophene—a newly synthesized material—has been successfully produced through various methods, including physical and chemical exfoliation, substrate‐supported growth (such as MBE, ALD, and CVD), and a recent bottom‐up approach using boric acid and CTAB surfactant, several challenges persist. Borophene has shown potential in applications like light sensing, molecular detection, strain and gas sensing, energy storage, and electrocatalysis. However, significant advancements are still needed to address the synthetic challenges and integration issues for device fabrication.

Key challenges in synthesis include achieving high chemical phase purity without oxidation or surface functionalization, as well as ensuring scalable and reproducible production. To achieve effective exfoliation while avoiding surface oxidation, it is recommended to use high‐surface‐energy solvents or solvents with high Hansen parameters during sonication or solvothermal treatments. Additionally, intercalating agents such as ionic solvents or Li+/Na+/K+ ions can aid exfoliation. The use of high‐energy plasma (e.g., laser‐based photo‐exfoliation) can create favorable thermodynamic conditions for volume expansion in parent crystals. Cryo exfoliation, utilizing liquid nitrogen to induce compressive thermal stress in boron crystals, can also facilitate exfoliation. These novel techniques show promise for producing high‐quality borophene. Recent advances in topotactic transformations, previously applied to metallenes, may also be leveraged for borophene, such as transforming MgB_2_ into borophene. However, such transformations will likely lead to structural changes, which can be stabilized through intermediate structures. The use of transition metal substrates, such as silver, gold, or copper, can help stabilize the borophene lattice.

Surface oxidation remains a significant concern, as it significantly impacts device performance, particularly in electronics, spintronics, and straintronics. Effective passivation with materials such as graphene, BN, alumina, or MgO could mitigate this issue. While borophene is more chemically and electrochemically active than pure graphene, enhancing its electrochemical and catalytic properties will require strategies such as controlled surface defect generation, substitutional doping, 2D‐2D hybridization, and surface functionalization. In heterolayer devices, where prompt charge or energy transfer is critical, poor‐quality interfaces are a major obstacle. For applications like electronic cooling or electrocatalytic hydrogen/oxygen production, fast electron transfer is necessary, and layer‐by‐layer self‐assembly could improve interface quality. Additionally, transferring CVD‐coated sheets in a layer‐by‐layer manner, followed by heating and laser shock treatments, could further enhance interfacial quality. The careful selection of substrates and electrode materials optimized for borophene electronics is also essential. To reduce the boron‐electrode metal interfacial contact resistance and improve work function engineering, a thin, soft metal coating could be beneficial.

Borophene has attracted extensive review attention due to its unique structural, electronic, mechanical, thermal, and chemical properties and broad potential applications. It exhibits anisotropic metallic behavior, high electrical and thermal conductivities, and structural polymorphism that enables tunability of its properties, along with exceptional mechanical strength, flexibility, and directional stiffness. Reviews consistently highlight its promising roles in energy storage and conversion (e.g., battery electrodes, hydrogen storage), sensing and gas capture, catalysis, and flexible nano‐ and optoelectronic devices, as well as emerging biomedical and environmental applications. Synthesis advancements on metal substrates and via various deposition techniques have been explored, yet challenges remain in achieving large‐area, stable, and scalable borophene. Reviews also emphasize the influence of defects, doping, heterostructures, and strain on tailoring performance, while noting that instability in ambient conditions and difficulties in mass production are key obstacles before practical deployment.

The present review showcases the challenges and way forward to tackle those challenges and it outlines borophene‐specific applications, especially futuristic devices and sensors.

## 7 Summary and Outlook

Borophene synthesis and borophene‐based devices face several interconnected challenges, primarily related to stability, scalability, phase control, and integration. Borophene is intrinsically chemically reactive and readily oxidizes in air, making both synthesis and device operation difficult under ambient conditions; this can be addressed through encapsulation, passivation, or chemical functionalization. Current synthesis methods, such as molecular beam epitaxy on metal substrates, are costly, substrate‐limited, and difficult to scale, while also producing multiple polymorphs that complicate phase and property control; advances in scalable growth techniques (e.g., CVD‐based routes), substrate engineering, and precise tuning of growth parameters are needed to achieve uniform, large‐area films with controlled structures. For device applications, challenges include poor environmental stability, high contact resistance, and damage or contamination during transfer from growth substrates to insulating device platforms; these issues can be mitigated by developing clean transfer methods, interface and contact engineering, and integrating borophene into protected heterostructures with other 2D materials. Overall, progress requires simultaneous improvements in synthesis scalability, structural control, stability enhancement, and device‐level integration strategies.

Recent advances in borophene research indicate that future work will focus on developing scalable, stable synthesis methods (including large‐area growth, substrate engineering, doping, and functionalization) to overcome current challenges in environmental stability and mass production, enabling integration into real devices; alongside this, tunable properties and heterostructures are being explored to tailor borophene for next‐generation electronics, flexible and high‐performance sensors, energy storage and conversion systems, and biomedical interfaces, with predictive design (e.g., AI‐assisted modeling) and novel fabrication strategies accelerating its transition from lab‐scale synthesis to practical applications in nanoelectronics, optoelectronics, catalysts and biosensing platforms.

Borophene has rapidly emerged as a versatile platform for next‐generation devices due to its unique combination of mechanical flexibility, metallic conductivity, and tunable electronic structure. Salient borophene‐based devices include ultrafast transistors, flexible sensors, high‐capacity batteries, supercapacitors, and hydrogen evolution or CO_2_ reduction catalysts, exploiting its high surface area and active sites. Beyond devices, borophene enables the exploration of novel physical phenomena such as anisotropic superconductivity, Dirac fermions, topological phases, and strain‐engineered electronic properties, opening new avenues in quantum materials research. Emerging technologies leverage these features in heterostructures, nanoscale energy conversion, spintronics, and nanoelectromechanical systems (NEMS), while computational design and AI‐guided synthesis are accelerating discovery, positioning borophene as a frontier material bridging fundamental science and transformative technological applications.

## Conflicts of Interest

The authors declare no conflict of interest.

## References

[1] D. N. Basov , R. D. Averitt , and D. Hsieh , “Towards Properties on Demand in Quantum Materials,” Nature Materials 16, no. 11 (2017): 1077–1088, 10.1038/nmat5017. 29066824

[2] B. Keimer and J. E. Moore , “The Physics of Quantum Materials,” Nature Physics 13, no. 11 (2017): 1045–1055, 10.1038/nphys4302.

[3] “Twenty Years of 2D Materials,” Nature Physics 2024, 20 (1), 1–1.

[4] H. Tang , Y. Wang , X. Ni , et al., “On‐Chip Multi‐Degree‐of‐Freedom Control of Two‐Dimensional Materials,” Nature 632, no. 8027 (2024): 1038–1044, 10.1038/s41586-024-07826-x. 39169189

[5] Y. Guo , J. Li , X. Zhan , et al., “Van der Waals Polarity‐engineered 3D Integration of 2D Complementary Logic,” Nature 630, no. 8016 (2024): 346–352, 10.1038/s41586-024-07438-5. 38811731 PMC11168927

[6] S. Chen , Z. Liang , J. Miao , et al., “Infrared Optoelectronics in Twisted Black Phosphorus,” Nature Communications 15, no. 1 (2024): 8834, 10.1038/s41467-024-53125-4. PMC11471851 39397018

[7] Q. Lin , H. Fang , A. Kalaboukhov , et al., “Moiré‐engineered Light‐matter Interactions in MoS_2_/WSe_2_ Heterobilayers at Room Temperature,” Nature Communications 15, no. 1 (2024): 8762, 10.1038/s41467-024-53083-x. PMC11464769 39384821

[8] H. Lee , S. Kim , S. Eom , et al., “Quantum Tunneling High‐speed Nano‐excitonic Modulator,” Nature Communications 15, no. 1 (2024): 8725, 10.1038/s41467-024-52813-5. PMC11461740 39379364

[9] Y. Wu , Z. Zeng , H. Lu , et al., “Coexistence of Ferroelectricity and Antiferroelectricity in 2D van der Waals Multiferroic,” Nature Communications 15, no. 1 (2024): 8616, 10.1038/s41467-024-53019-5. PMC11452644 39366986

[10] S. Lee , D. H. Ho , J. Jekal , et al., “Fabric‐based Lamina Emergent MXene‐based Electrode for Electrophysiological Monitoring,” Nature Communications 15, no. 1 (2024): 5974, 10.1038/s41467-024-49939-x. PMC11446925 39358330

[11] M. C. Lemme , D. Akinwande , C. Huyghebaert , and C. Stampfer , “2D materials for Future Heterogeneous Electronics,” Nature Communications 13, no. 1 (2022): 1392, 10.1038/s41467-022-29001-4. PMC8927416 35296657

[12] M. Turunen , M. Brotons‐Gisbert , Y. Dai , et al., “Quantum Photonics with Layered 2D Materials,” Nature Reviews Physics 4, no. 4 (2022): 219–236, 10.1038/s42254-021-00408-0.

[13] P. Ranjan , S. Gaur , H. Yadav , et al., “2D materials: Increscent Quantum Flatland with Immense Potential for Applications,” Nano Convergence 9, no. 1 (2022): 26, 10.1186/s40580-022-00317-7. 35666392 PMC9170864

[14] P. Kumar , “Laser Flash Synthesis of Graphene and Its Inorganic Analogues: an Innovative Breakthrough with Immense Promise,” RSC Advances 3, no. 30 (2013): 11987–12002, 10.1039/c3ra41149d.

[15] K. S. Novoselov , A. K. Geim , S. V. Morozov , et al., “Electric Field Effect in Atomically Thin Carbon Films,” Science 306, no. 5696 (2004): 666–669, 10.1126/science.1102896. 15499015

[16] A. K. Geim and K. S. Novoselov , “The Rise of Graphene,” Nature Materials 6, no. 3 (2007): 183–191, 10.1038/nmat1849. 17330084

[17] A. K. Geim , “Graphene: Status and Prospects,” Science 324, no. 5934 (2009): 1530–1534, 10.1126/science.1158877. 19541989

[18] K. S. Subrahmanyam , P. Kumar , U. Maitra , et al., “Chemical Storage of Hydrogen in Few‐layer Graphene,” Proceedings of the National Academy of Sciences 108, no. 7 (2011): 2674–2677, 10.1073/pnas.1019542108. PMC3041143 21282617

[19] P. Kumar , A. Dey , J. Roques , et al., “Photoexfoliation Synthesis of 2D Materials,” ACS Materials Letters 4, no. 2 (2022): 263–270, 10.1021/acsmaterialslett.1c00651.

[20] P. Kumar , S. S. R. K. C. Yamijala , and S. K. Pati , “Optical Unzipping of Carbon Nanotubes in Liquid Media,” The Journal of Physical Chemistry C 120, no. 30 (2016): 16985–16993, 10.1021/acs.jpcc.6b02524.

[21] H. O. H. Churchill and P. Jarillo‐Herrero , “Phosphorus Joins the family,” Nature Nanotechnology 9, no. 5 (2014): 330–331, 10.1038/nnano.2014.85. 24801536

[22] S. Chahal , R. Bhushan , P. Kumari , et al., “Microwave Nanoarchitectonics of Black Phosphorene for Energy Storage,” Matter 7, no. 1 (2024): 237–254, 10.1016/j.matt.2023.10.030.

[23] L. Tao , E. Cinquanta , D. Chiappe , et al., “Silicene Field‐effect Transistors Operating at Room Temperature,” Nature Nanotechnology 10, no. 3 (2015): 227–231, 10.1038/nnano.2014.325. 25643256

[24] M. E. Dávila , L. Xian , S. Cahangirov , A. Rubio , and G. Le Lay , “Germanene: a Novel Two‐dimensional Germanium Allotrope Akin to Graphene and Silicene,” New Journal of Physics 16, no. 9 (2014): 095002, 10.1088/1367-2630/16/9/095002.

[25] F.‐F. Zhu , W.‐F. Chen , Y. Xu , et al., “Epitaxial Growth of Two‐dimensional Stanene,” Nature Materials 14, no. 10 (2015): 1020–1025, 10.1038/nmat4384. 26237127

[26] M. Pumera and Z. Sofer , “2D Monoelemental Arsenene, Antimonene, and Bismuthene: Beyond Black Phosphorus,” Advanced Materials 29, no. 21 (2017): 1605299, 10.1002/adma.201605299. 28185366

[27] A. J. Mannix , X.‐F. Zhou , B. Kiraly , et al., “Synthesis of Borophenes: Anisotropic, Two‐dimensional Boron Polymorphs,” Science 350, no. 6267 (2015): 1513–1516. 26680195 10.1126/science.aad1080 PMC4922135

[28] P. Ranjan , T. K. Sahu , R. Bhushan , et al., “Freestanding Borophene and its Hybrids,” Advanced Materials 31, no. 27 (2019): 1900353, 10.1002/adma.201900353. 31044470

[29] P. Ranjan , J. M. Lee , P. Kumar , and A. Vinu , “Borophene: New Sensation in Flatland,” Advanced Materials 32, no. 34 (2020): 2000531. 10.1002/adma.202000531 32666554

[30] S. Chahal , P. Ranjan , M. Motlag , et al., “Borophene via Micromechanical Exfoliation,” Advanced Materials 33, no. 34 (2021): 2102039. 10.1002/adma.202102039 34270846

[31] X. Guan , P. Kumar , Z. Li , et al., “Borophene Embedded Cellulose Paper for Enhanced Photothermal Water Evaporation and Prompt Bacterial Killing,” Advanced Science 10, no. 7 (2023): 2205809. 36698305 10.1002/advs.202205809 PMC9982542

[32] K. Vishwakarma , S. Rani , S. Chahal , et al., “Quantum‐coupled Borophene‐based Heterolayers for Excitonic and Molecular Sensing Applications,” Physical Chemistry Chemical Physics 24, no. 21 (2022): 12816–12826, 10.1039/D2CP01712A. 35608151

[33] V. Kochat , A. Samanta , Y. Zhang , et al., “Atomically Thin Gallium Layers from Solid‐melt Exfoliation,” Science Advances 4, no. 3 (2018): 1701373, 10.1126/sciadv.1701373. PMC5844710 29536039

[34] S. Chahal , A. Bandyopadhyay , C.‐S. Yang , and P. Kumar , “Beryllene, the Lightest Xene,” npj 2D Materials and Applications 7, no. 1 (2023): 55.

[35] S. Chahal , A. Bandyopadhyay , S. P. Dash , and P. Kumar , “Microwave Synthesized 2D Gold and Its 2D‐2D Hybrids,” The Journal of Physical Chemistry Letters 13, no. 28 (2022): 6487–6495, 10.1021/acs.jpclett.2c01540. 35819242

[36] T. K. Sahu , N. Kumar , S. Chahal , et al., “Microwave Synthesis of Molybdenene from MoS_2_ ,” Nature Nanotechnology 18, no. 12 (2023): 1430–1438, 10.1038/s41565-023-01484-2. PMC10716048 37666941

[37] Q. Mao , X. Mu , W. Wang , et al., “Atomically Dispersed Cu Coordinated Rh Metallene Arrays for Simultaneously Electrochemical Aniline Synthesis and Biomass Upgrading,” Nature Communications 14, no. 1 (2023): 5679, 10.1038/s41467-023-41423-2. PMC10502102 37709775

[38] Q. Dang , H. Lin , Z. Fan , et al., “Iridium Metallene Oxide for Acidic Oxygen Evolution Catalysis,” Nature Communications 12, no. 1 (2021): 6007, 10.1038/s41467-021-26336-2. PMC8516950 34650084

[39] S. Manzeli , D. Ovchinnikov , D. Pasquier , O. V. Yazyev , and A. Kis , “2D transition Metal Dichalcogenides,” Nature Reviews Materials 2, no. 8 (2017): 17033, 10.1038/natrevmats.2017.33.

[40] B. Anasori , M. R. Lukatskaya , and Y. Gogotsi , “2D metal Carbides and Nitrides (MXenes) for Energy Storage,” Nature Reviews Materials 2, no. 2 (2017): 16098, 10.1038/natrevmats.2016.98.

[41] S. Chahal , S. M. Kauzlarich , and P. Kumar , “Microwave Synthesis of Hematene and Other Two‐Dimensional Oxides,” ACS Materials Letters 3, no. 5 (2021): 631–640, 10.1021/acsmaterialslett.1c00102.

[42] S. Chahal , T. K. Sahu , S. Kar , H. Ranjan , S. J. Ray , and P. Kumar , “Free‐Standing δ‐MnO_2_ Atomic Sheets,” Engineering Reports 6, no. 2 (2024): 12787, 10.1002/eng2.12787.

[43] P. Ranjan and P. Kumar , “White Lead: A New Naturally Occurring 2D Material,” Journal of Materials Research 37, no. 20 (2022): 3352–3361, 10.1557/s43578-022-00655-6.

[44] I. Pradhan , A. Mahapatra , A. Bandyopadhyay , A. Nayak , and P. Kumar , “Ag‐Doped Free‐Standing 2D TiO_2_ Sheets: Electronic, Optical, Magnetic, and Self‐Healing Behaviour,” Chemphyschem 24, no. 24 (2023): 202300447, 10.1002/cphc.202300447. 37732481

[45] I. Pradhan , A. Mahapatra , P. P. Samal , P. Mishra , P. Kumar , and A. Nayak , “Liquid–Liquid Interface‐Assisted Self‐Assembly of Ag‐Doped ZnO Nanosheets for Atomic Switch Application,” The Journal of Physical Chemistry Letters 15, no. 1 (2024): 165–172, 10.1021/acs.jpclett.3c02791. 38150295

[46] P. Kumar , J. Liu , P. Ranjan , et al., “Alpha Lead Oxide (α‐PbO): a New 2D Material with Visible Light Sensitivity,” Small 14, no. 12 (2018): 1703346. 10.1002/smll.201703346 29430851

[47] S. Mishra , T. K. Sahu , P. Verma , P. Kumar , and S. K. Samanta , “Microwave‐Assisted Catalytic Degradation of Brilliant Green by Spinel Zinc Ferrite Sheets,” ACS Omega 4, no. 6 (2019): 10411–10418, 10.1021/acsomega.9b00914. 31460135 PMC6648797

[48] S. Mishra , P. Kumar , and S. K. Samanta , “Microwave Catalytic Degradation of Antibiotic Molecules by 2D Sheets of Spinel Nickel Ferrite,” Industrial & Engineering Chemistry Research 59, no. 36 (2020): 15839–15847.

[49] S. Mishra , S. Kumari , P. Kumar , and S. K. Samanta , “Microwave Synthesized Strontium Hexaferrite 2D Sheets as Versatile and Efficient Microwave Catalysts for Degradation of Organic Dyes and Antibiotics,” Science of The Total Environment 790 (2021): 147853, 10.1016/j.scitotenv.2021.147853. 34087737

[50] T. K. Sahu , P. Ranjan , and P. Kumar , “Chemical Exfoliation Synthesis of Boron Nitride and Molybdenum Disulfide 2D Sheets via Modified Hummers' method,” Emergent Materials 4, no. 3 (2021): 645–654, 10.1007/s42247-021-00170-0.

[51] P. Kumar , G. Singh , X. Guan , et al., “Multifunctional Carbon Nitride Nanoarchitectures for Catalysis,” Chemical Society Reviews 52, no. 21 (2023): 7602–7664, 10.1039/D3CS00213F. 37830178

[52] A. M. Sadanandan , J.‐H. Yang , V. Devtade , et al., “Carbon Nitride Based Nanoarchitectonics for Nature‐inspired Photocatalytic CO2 Reduction,” Progress in Materials Science 142 (2024): 101242, 10.1016/j.pmatsci.2024.101242.

[53] P. Kumar , D. Laishram , R. K. Sharma , A. Vinu , J. Hu , and M. G. Kibria , “Boosting Photocatalytic Activity Using Carbon Nitride Based 2D/2D van der Waals Heterojunctions,” Chemistry of Materials 33, no. 23 (2021): 9012–9092, 10.1021/acs.chemmater.1c03166.

[54] S. Kim , M. Hankel , W. Cha , et al., “Theoretical and Experimental Investigations of Mesoporous C_3_N_5_/MoS_2_ Hybrid for Lithium and Sodium Ion Batteries,” Nano Energy 72 (2020): 104702, 10.1016/j.nanoen.2020.104702.

[55] T. K. Sahu , S. P. Sahu , K. P. S. S. Hembram , J.‐K. Lee , V. Biju , and P. Kumar , “Free‐standing 2D Gallium Nitride for Electronic, Excitonic, Spintronic, Piezoelectric, Thermoplastic, and 6G Wireless Communication Applications,” NPG Asia Materials 15, no. 1 (2023): 49, 10.1038/s41427-023-00497-6.

[56] J. Jin and U. Schwingenschlögl , “Exploration of the Two‐dimensional Transition Metal Phosphide MoP_2_ as Anode for Na/K Ion Batteries,” npj 2D Materials and Applications 8, no. 1 (2024): 31.

[57] J. Jin and U. Schwingenschlögl , “Exploration of Two‐dimensional Molybdenum‐borides and Potential Applications,” npj 2D Materials and Applications 6, no. 1 (2022): 49.

[58] M. Motlag , P. Kumar , K. Y. Hu , et al., “Asymmetric 3D Elastic–Plastic Strain‐Modulated Electron Energy Structure in Monolayer Graphene by Laser Shocking,” Advanced Materials 31, no. 19 (2019): 1900597, 10.1002/adma.201900597. 30924972

[59] S. Chahal , A. K. Nair , S. J. Ray , J. Yi , A. Vinu , and P. Kumar , “Microwave Flash Synthesis of Phosphorus and Sulphur Ultradoped Graphene,” Chemical Engineering Journal 450 (2022): 138447, 10.1016/j.cej.2022.138447.

[60] R. Bhushan , A. Bandyopadhyay , S. Kallatt , A. K. Thakur , S. K. Pati , and P. Kumar , “Microwave Graphitic Nitrogen/Boron Ultradoping of Graphene,” npj 2D Materials and Applications 8, no. 1 (2024): 19, 10.1038/s41699-024-00457-w.

[61] Z. Li , X. Guan , G. Pandey , et al., “Microwave Doping of Sulfur and Iron in β12 Borophene,” Small 20, no. 39 (2024): 2307610, 10.1002/smll.202307610. 38342695

[62] P. Kumar , J. Liu , M. Motlag , et al., “Laser Shock Tuning Dynamic Interlayer Coupling in Graphene–Boron Nitride Moiré Superlattices,” Nano Letters 19, no. 1 (2019): 283–291, 10.1021/acs.nanolett.8b03895. 30525695

[63] T. K. Sahu , M. Motlag , A. Bandyopadhyay , N. Kumar , G. J. Cheng , and P. Kumar , “2+δ‐Dimensional Materials via Atomistic Z‐Welding,” Advanced Science 9, no. 32 (2022): 2202695. 36089664 10.1002/advs.202202695 PMC9661819

[64] A. Pal , S. Zhang , T. Chavan , et al., “Quantum‐Engineered Devices Based on 2D Materials for Next‐Generation Information Processing and Storage,” Advanced Materials 35, no. 27 (2023): 2109894, 10.1002/adma.202109894. 35468661

[65] “Doping in 2D,” Nature Electronics 2021, 4 (10), 699–699.

[66] G. Guan and M.‐Y. Han , “Functionalized Hybridization of 2D Nanomaterials,” Advanced Science 6, no. 23 (2019): 1901837, 10.1002/advs.201901837. 31832321 PMC6891915

[67] L. Jia , J. Wu , Y. Zhang , et al., “Fabrication Technologies for the on‐Chip Integration of 2D Materials,” Small Methods 6, no. 3 (2022): 2101435, 10.1002/smtd.202101435. 34994111

[68] D. Li , J. Gao , P. Cheng , et al., “2D Boron Sheets: Structure, Growth, and Electronic and Thermal Transport Properties,” Advanced Functional Materials 30, no. 8 (2020): 1904349.

[69] B. Feng , J. Zhang , S. Ito , et al., “Discovery of 2D Anisotropic Dirac Cones,” Advanced Materials 30, no. 2 (2018): 1704025, 10.1002/adma.201704025. 29171690

[70] Y. V. Kaneti , D. P. Benu , X. Xu , B. Yuliarto , Y. Yamauchi , and D. Golberg , “Borophene: Two‐dimensional Boron Monolayer: Synthesis, Properties, and Potential Applications,” Chemical Reviews 122, no. 1 (2022): 1000–1051, 10.1021/acs.chemrev.1c00233. 34730341

[71] D. Sun , H. Liu , H. Liang , et al., “Unravelling the Mechanical and Superconducting Properties in Borophene with Multicentered Bonds,” The Journal of Physical Chemistry Letters 16 (2025): 494–502, 10.1021/acs.jpclett.4c03294. 39749897

[72] T. Abasi , A. Boochani , and S. R. Masharian , “Metallic and Intra‐band Investigation of Optical Properties for Borophene Nano‐sheet: a DFT Study,” International Nano Letters 10, no. 1 (2020): 33–41, 10.1007/s40089-019-00288-4.

[73] K. Wang , S. Choyal , J. F. Schultz , et al., “Borophene: Synthesis, Chemistry, and Electronic Properties,” ChemPlusChem 89, no. 10 (2024): 202400333, 10.1002/cplu.202400333. 39031807

[74] B. N. Šoškić , J. Bekaert , C. Sevik , and M. V. Milošević , “Enhanced Superconductivity of Hydrogenated β12 Borophene,” Nano Letters 24, no. 40 (2024): 12650–12657. 39316522 10.1021/acs.nanolett.4c03845

[75] H. Zhou , Y. Cai , G. Zhang , and Y.‐W. Zhang , “Superior Lattice Thermal Conductance of Single‐layer Borophene,” npj 2D Materials and Applications 1, no. 1 (2017): 14, 10.1038/s41699-017-0018-2.

[76] Y. Duo , Z. Xie , L. Wang , et al., “Borophene‐based Biomedical Applications: Status and Future Challenges,” Coordination Chemistry Reviews 427 (2021): 213549.

[77] Y. Abdi , A. Mazaheri , S. Hajibaba , et al., “A Two‐Dimensional Borophene Supercapacitor,” ACS Materials Letters 4, no. 10 (2022): 1929–1936, 10.1021/acsmaterialslett.2c00475.

[78] J. Yu , M. Zhou , M. Yang , et al., “Pristine and Defective 2D Borophene/Graphene Heterostructure as the Potential Anode of Lithium‐Ion Batteries,” Advanced Materials Interfaces 9, no. 12 (2022): 2102088, 10.1002/admi.202102088.

[79] W. Mabhulusa , K. E. Sekhosana , and X. Fuku , “Incorporation of Pd Catalyst into Highly Effective Borophene Nanosheet Co‐Catalyst for Electrokinetics and Electrochemical Oxygen Reduction Reactions,” Journal of Electronic Materials 53, no. 7 (2024): 4236–4249, 10.1007/s11664-024-11113-w.

[80] J. C. Alvarez‐Quiceno , R. H. Miwa , G. M. Dalpian , and A. Fazzio , “Oxidation of Free‐standing and Supported Borophene,” 2D Materials 4, no. 2 (2017): 025025.

[81] J. Casanova‐Chafer , “Roadmap for Borophene Gas Sensors,” ACS Sensors 10, no. 1 (2025): 76–99, 10.1021/acssensors.4c03164. 39754599

[82] Z. Wu , X. Liang , Y. Liu , M. Xu , R. Zhu , and G. Tai , “Synthesis and Anisotropic Memristive Behavior of Borophene Nanosheets,” Angewandte Chemie International Edition n/a, no. n/a (2025): 202416041. 10.1002/anie.202416041 39223089

[83] W. L. Scopel , F. Crasto de Lima , P. H. Souza , J. E. Padilha , and R. H. Miwa , “Bridging Borophene and Metal Surfaces: Structural, Electronic, and Electron Transport Properties,” The Journal of Physical Chemistry C 127, no. 35 (2023): 17556–17566, 10.1021/acs.jpcc.3c03123.

[84] I. Boustani , “Structure and Stability of Small Boron Clusters. A Density Functional Theoretical Study,” Chemical Physics Letters 240, no. 1 (1995): 135–140, 10.1016/0009-2614(95)00510-B.

[85] W.‐L. Li , X. Chen , T. Jian , T.‐T. Chen , J. Li , and L.‐S. Wang , “From Planar Boron Clusters to Borophenes and Metalloborophenes,” Nature Reviews Chemistry 1, no. 10 (2017): 0071, 10.1038/s41570-017-0071.

[86] K. C. Lau and R. Pandey , “Stability and Electronic Properties of Atomistically‐Engineered 2D Boron Sheets,” The Journal of Physical Chemistry C 111, no. 7 (2007): 2906–2912, 10.1021/jp066719w.

[87] Z. A. Piazza , H.‐S. Hu , W.‐L. Li , Y.‐F. Zhao , J. Li , and L.‐S. Wang , “Planar Hexagonal B36 as a Potential Basis for Extended Single‐atom Layer Boron Sheets,” Nature Communications 5, no. 1 (2014): 3113, 10.1038/ncomms4113. 24445427

[88] N. Karmodak , E. D. Jemmis , and B. I. Yakobson , “Borophenes: Insights and Predictions from Computational Analyses,” in 2D Boron: Boraphene, Borophene, Boronene, ed. I. Matsuda and K. Wu (Springer International Publishing, 2021), 27–49.

[89] G. Parakhonskiy , N. Dubrovinskaia , E. Bykova , R. Wirth , and L. Dubrovinsky , “Experimental Pressure‐temperature Phase Diagram of Boron: Resolving the Long‐standing Enigma,” Scientific Reports 1, no. 1 (2011): 96, 10.1038/srep00096. 22355614 PMC3216582

[90] S. Zhang , H. D. Whitley , and T. Ogitsu , “Phase Transformation in Boron under Shock Compression,” Solid State Sciences 108 (2020): 106376, 10.1016/j.solidstatesciences.2020.106376.

[91] Y. Park , Y. Wang , V. Gladkikh , D. Hedman , X. Kong , and F. Ding , “High Temperature Phases of Borophene: Borophene Glass and Liquid,” Nanoscale Horizons 8, no. 3 (2023): 353–360, 10.1039/D2NH00518B. 36722748

[92] B. Feng , J. Zhang , Q. Zhong , et al., “Experimental Realization of Two‐dimensional Boron Sheets,” Nature Chemistry 8, no. 6 (2016): 563–568, 10.1038/nchem.2491. 27219700

[93] C. Chen , H. Lv , P. Zhang , et al., “Synthesis of Bilayer Borophene,” Nature Chemistry 14, no. 1 (2022): 25–31, 10.1038/s41557-021-00813-z. 34764470

[94] G. Tai , T. Hu , Y. Zhou , et al., “Synthesis of Atomically Thin Boron Films on Copper Foils,” Angewandte Chemie International Edition 54, no. 51 (2015): 15473–15477, 10.1002/anie.201509285. 26510179

[95] A. Mazaheri , M. Javadi , and Y. Abdi , “Chemical Vapor Deposition of Two‐Dimensional Boron Sheets by Thermal Decomposition of Diborane,” ACS Applied Materials & Interfaces 13, no. 7 (2021): 8844–8850, 10.1021/acsami.0c22580. 33565849

[96] X. Liang , C. Hou , Z. Wu , Z. Wu , and G. Tai , “Multilayer α′‐4H‐borophene Growth on Gallium Arsenide towards High‐performance near‐infrared Photodetector,” Nanotechnology 34, no. 20 (2023): 205701, 10.1088/1361-6528/acba1e. 36753755

[97] Z. Wu , G. Tai , R. Liu , W. Shao , C. Hou , and X. Liang , “Synthesis of Borophene on Quartz towards Hydroelectric Generators,” Journal of Materials Chemistry A 10, no. 15 (2022): 8218–8226, 10.1039/D1TA10855G.

[98] Z. Wu , G. Tai , R. Liu , et al., “van der Waals Epitaxial Growth of Borophene on a Mica Substrate toward a High‐Performance Photodetector,” ACS Applied Materials & Interfaces 13, no. 27 (2021): 31808–31815, 10.1021/acsami.1c03146. 34213879

[99] T. Aizawa , S. Suehara , and S. Otani , “Phonon Dispersion of a Two‐dimensional Boron Sheet on Ag(111),” Physical Review Materials 5, no. 6 (2021): 064004, 10.1103/PhysRevMaterials.5.064004.

[100] X. Liu , L. Wang , B. I. Yakobson , and M. C. Hersam , “Nanoscale Probing of Image‐Potential States and Electron Transfer Doping in Borophene Polymorphs,” Nano Letters 21, no. 2 (2021): 1169–1174, 10.1021/acs.nanolett.0c04869. 33455160

[101] Y. Xu , X. Xuan , T. Yang , Z. Zhang , S.‐D. Li , and W. Guo , “Quasi‐Freestanding Bilayer Borophene on Ag(111),” Nano Letters 22, no. 8 (2022): 3488–3494, 10.1021/acs.nanolett.1c05022. 35341246

[102] B. Kiraly , X. Liu , L. Wang , et al., “Borophene Synthesis on Au(111),” ACS Nano 13, no. 4 (2019): 3816–3822. 30844248 10.1021/acsnano.8b09339

[103] C. Sharma , M. K. Gupta , S. Badatya , A. K. Srivastava , and N. Sathish , “Blue Light Emitting Piezoelectric Few‐layered Borophene Nanosheets for Flexible Nanogenerators,” Communications Materials 4, no. 1 (2023): 54, 10.1038/s43246-023-00375-2.

[104] H. Li , L. Jing , W. Liu , et al., “Scalable Production of Few‐Layer Boron Sheets by Liquid‐Phase Exfoliation and Their Superior Supercapacitive Performance,” ACS Nano 12, no. 2 (2018): 1262–1272, 10.1021/acsnano.7b07444. 29378394

[105] J. Qin , X. Wang , Q. Jiang , and M. Cao , “Optimizing Dispersion, Exfoliation, Synthesis, and Device Fabrication of Inorganic Nanomaterials Using Hansen Solubility Parameters,” Chemphyschem 20, no. 9 (2019): 1069–1097, 10.1002/cphc.201900110. 30900364

[106] A. Sharma , U. Bhardwaj , M. Marinova , et al., “Borophene: a Piezocatalyst for Water Remediation,” Chemical Communications 60, no. 43 (2024): 5614–5617, 10.1039/D4CC00463A. 38713495

[107] Q. Li , V. S. C. Kolluru , M. S. Rahn , et al., “Synthesis of Borophane Polymorphs through Hydrogenation of Borophene,” Science 371, no. 6534 (2021): 1143–1148, 10.1126/science.abg1874. 33707261

[108] X. Zeng , Y. Jing , S. Gao , et al., “Hydrogenated Borophene Enabled Synthesis of Multielement Intermetallic Catalysts,” Nature Communications 14, no. 1 (2023): 7414, 10.1038/s41467-023-43294-z. PMC10654666 37973849

[109] K. Sielicki , K. Maślana , X. Chen , and E. Mijowska , “Bottom up Approach of Metal Assisted Electrochemical Exfoliation of Boron towards Borophene,” Scientific Reports 12, no. 1 (2022): 15683, 10.1038/s41598-022-20130-w. 36127387 PMC9489866

[110] L. Guo , H. Wang , Z. Wu , et al., “Li‐intercalation Chemistry of Few‐layer Borophene with Ultrahigh Capacity and Fast Kinetics,” Chemical Engineering Journal 473 (2023): 145017, 10.1016/j.cej.2023.145017.

[111] B. Yong , Y. Li , G. Li , et al., “New Strategy for Preparation of High Quality Borophene and Thermodynamics Analysis,” Materials Today Communications 44 (2025): 111887.

[112] A. A. Balandin , “Thermal Properties of Graphene and Nanostructured Carbon Materials,” Nature Materials 10, no. 8 (2011): 569–581, 10.1038/nmat3064. 21778997

[113] S. Milana , “Phosphorus in the Flatland,” Nature Nanotechnology 16, no. 10 (2021): 1055–1055, 10.1038/s41565-021-00993-2. 34625718

[114] A. Castellanos‐Gomez , L. Vicarelli , E. Prada , et al., “Isolation and Characterization of Few‐layer Black Phosphorus,” 2D Materials 1, no. 2 (2014): 025001.

[115] Y. Kubota , K. Watanabe , O. Tsuda , and T. Taniguchi , “Deep Ultraviolet Light‐Emitting Hexagonal Boron Nitride Synthesized at Atmospheric Pressure,” Science 317, no. 5840 (2007): 932–934, 10.1126/science.1144216. 17702939

[116] F. Roccaforte and M. Leszczynski , “Introduction to Gallium Nitride Properties and Applications,” In Nitride Semiconductor Technology (2020): 1–39.

[117] A. A. Tedstone , D. J. Lewis , and P. O'Brien , “Synthesis, Properties, and Applications of Transition Metal‐Doped Layered Transition Metal Dichalcogenides,” Chemistry of Materials 28, no. 7 (2016): 1965–1974, 10.1021/acs.chemmater.6b00430.

[118] X. Duan and H. Zhang , “Introduction: Two‐Dimensional Layered Transition Metal Dichalcogenides,” Chemical Reviews 124, no. 19 (2024): 10619–10622, 10.1021/acs.chemrev.4c00586. 39380397

[119] M. Naguib , V. N. Mochalin , M. W. Barsoum , and Y. Gogotsi , “25th Anniversary Article: MXenes: a New Family of Two‐Dimensional Materials,” Advanced Materials 26, no. 7 (2014): 992–1005. 24357390 10.1002/adma.201304138

[120] Y. Gogotsi and B. Anasori , “The Rise of MXenes,” ACS Nano 13, no. 8 (2019): 8491–8494, 10.1021/acsnano.9b06394. 31454866

[121] J. L. Hart , K. Hantanasirisakul , A. C. Lang , et al., “Control of MXenes' electronic Properties through Termination and Intercalation,” Nature Communications 10, no. 1 (2019): 522, 10.1038/s41467-018-08169-8. PMC6355901 30705273

[122] M. Tawalbeh , H. A. Khan , and A. Al‐Othman , “Insights on the Applications of Metal Oxide Nanosheets in Energy Storage Systems,” Journal of Energy Storage 60 (2023): 106656, 10.1016/j.est.2023.106656.

[123] J. E. ten Elshof , H. Yuan , and P. Gonzalez Rodriguez , “Two‐Dimensional Metal Oxide and Metal Hydroxide Nanosheets: Synthesis, Controlled Assembly and Applications in Energy Conversion and Storage,” Advanced Energy Materials 6, no. 23 (2016): 1600355, 10.1002/aenm.201600355.

[124] A. J. Khan , S. S. Shah , S. Khan , et al., “2D metal Borides (MBenes): Synthesis Methods for Energy Storage Applications,” Chemical Engineering Journal 497 (2024): 154429, 10.1016/j.cej.2024.154429.

[125] M. S. Javed , X. Zhang , T. Ahmad , et al., “MXenes to MBenes: Latest Development and Opportunities for Energy Storage Devices,” Materials Today 74 (2024): 121–148.

[126] M. Osada and T. Sasaki , “Two‐Dimensional Dielectric Nanosheets: Novel Nanoelectronics from Nanocrystal Building Blocks,” Advanced Materials 24, no. 2 (2012): 210–228, 10.1002/adma.201103241. 21997712

[127] T. A. Chowdhury , M. A. Bin Zafar , M. Sajjad‐Ul Islam , M. Shahinuzzaman , M. A. Islam , and M. U. Khandaker , “Stability of Perovskite Solar Cells: Issues and Prospects,” RSC Advances 13, no. 3 (2023): 1787–1810. 36712629 10.1039/d2ra05903g PMC9828105

[128] J. Zhao , H. Liu , Z. Yu , et al., “Rise of Silicene: a Competitive 2D Material,” Progress in Materials Science 83 (2016): 24–151, 10.1016/j.pmatsci.2016.04.001.

[129] A. Nijamudheen , R. Bhattacharjee , S. Choudhury , and A. Datta , “Electronic and Chemical Properties of Germanene: the Crucial Role of Buckling,” The Journal of Physical Chemistry C 119, no. 7 (2015): 3802–3809, 10.1021/jp511488m.

[130] S. Rani , K. Suganthi , and S. C. Roy , “Stanene: State of the Art and Future Prospects,” Journal of Electronic Materials 52, no. 6 (2023): 3563–3575, 10.1007/s11664-023-10377-y.

[131] S. Grillo , S. Postorino , M. Palummo , and O. Pulci , “Tellurene Polymorphs: a New Frontier for Solar Harvesting with Strong Exciton Anisotropy and High Optical Absorbance,” Advanced Energy Materials 14, no. 44 (2024): 2400674, 10.1002/aenm.202400674.

[132] Z. Gao , G. Liu , and J. Ren , “High Thermoelectric Performance in Two‐Dimensional Tellurium: an Ab Initio Study,” ACS Applied Materials & Interfaces 10, no. 47 (2018): 40702–40709, 10.1021/acsami.8b11836. 30394087

[133] J. A. Carrasco , P. Congost‐Escoin , M. Assebban , and G. Abellán , “Antimonene: a Tuneable Post‐graphene Material for Advanced Applications in Optoelectronics, Catalysis, Energy and Biomedicine,” Chemical Society Reviews 52, no. 4 (2023): 1288–1330, 10.1039/D2CS00570K. 36744431 PMC9987414

[134] R. K. Mishra , J. Sarkar , K. Verma , I. Chianella , S. Goel , and H. Y. Nezhad , “Borophene: a 2D Wonder Shaping the Future of Nanotechnology and Materials Science,” Nano Materials Science 7, no. 2 (2024): 198–230.

[135] X. Yan , S. Wang , Y. Sun , Y. Liu , Y. Wang , and G. Yang , “Semiconducting Bilayer Borophene with High Carrier Mobility,” The Journal of Physical Chemistry Letters 14, no. 43 (2023): 9698–9704, 10.1021/acs.jpclett.3c02684. 37875810

[136] J. Zhang , J. Zhang , L. Zhou , et al., “Universal Scaling of Intrinsic Resistivity in Two‐Dimensional Metallic Borophene,” Angewandte Chemie International Edition 57, no. 17 (2018): 4585–4589, 10.1002/anie.201800087. 29485742

[137] T. Cheng , H. Lang , Z. Li , Z. Liu , and Z. Liu , “Anisotropic Carrier Mobility in Two‐dimensional Materials with Tilted Dirac Cones: Theory and Application,” Physical Chemistry Chemical Physics 19, no. 35 (2017): 23942–23950, 10.1039/C7CP03736H. 28808705

[138] D. Li , J. He , G. Ding , et al., “Stretch‐Driven Increase in Ultrahigh Thermal Conductance of Hydrogenated Borophene and Dimensionality Crossover in Phonon Transmission,” Advanced Functional Materials 28, no. 31 (2018): 1801685, 10.1002/adfm.201801685.

[139] Y. Jiao , F. Ma , J. Bell , A. Bilic , and A. Du , “Two‐Dimensional Boron Hydride Sheets: High Stability, Massless Dirac Fermions, and Excellent Mechanical Properties,” Angewandte Chemie International Edition 55, no. 35 (2016): 10292–10295, 10.1002/anie.201604369. 27460282

[140] Y. He , N. Cheng , C. Chen , S. Xiong , and J. Zhao , “Tuning the Electronic Transport Anisotropy in Borophene via Oxidation Strategy,” Science China Technological Sciences 62, no. 5 (2019): 799–810, 10.1007/s11431-018-9385-x.

[141] Y.‐L. Zhang , J.‐H. Yang , H. Xiang , and X.‐G. Gong , “Fully Boron‐Sheet‐Based Field Effect Transistors from First‐Principles: Inverse Design of Semiconducting Boron Sheets,” The Journal of Physical Chemistry Letters 12, no. 1 (2021): 576–584, 10.1021/acs.jpclett.0c03333. 33382274

[142] C. Hou , G. Tai , B. Liu , Z. Wu , and Y. Yin , “Borophene‐graphene Heterostructure: Preparation and Ultrasensitive Humidity Sensing,” Nano Research 14, no. 7 (2021): 2337–2344, 10.1007/s12274-020-3232-8.

[143] B. Peng , H. Zhang , H. Shao , Y. Xu , R. Zhang , and H. Zhu , “The Electronic, Optical, and Thermodynamic Properties of Borophene from First‐principles Calculations,” Journal of Materials Chemistry 4, no. 16 (2016): 3592–3598.

[144] R. Yang and M. Sun , “Electronic Structures and Optical Properties of Monolayer Borophenes,” Spectrochimica Acta Part A: Molecular and Biomolecular Spectroscopy 272 (2022): 121014, 10.1016/j.saa.2022.121014. 35182919

[145] Z. Xie , X. Meng , X. Li , et al., “Two‐Dimensional Borophene: Properties, Fabrication, and Promising Applications,” Research 2020 (2020): 2624617. 32607497 10.34133/2020/2624617 PMC7312787

[146] T. Kambe , S. Imaoka , M. Shimizu , et al., “Liquid Crystalline 2D Borophene Oxide for Inorganic Optical Devices,” Nature Communications 13, no. 1 (2022): 1037, 10.1038/s41467-022-28625-w. PMC8873452 35210423

[147] G. Maity , P. K. Mishra , G. Patel , and S. Dubey , “Advances in Borophene Based Photodetectors for a Sustainable Tomorrow: a Comprehensive Review,” Nanoscale 16, no. 39 (2024): 18295–18318, 10.1039/D4NR02638A. 39279467

[148] C. Zhang , Z. Zhang , W. Yan , and X. Qin , “Effect of Doping on the Photoelectric Properties of Borophene,” Advances in Condensed Matter Physics 2021, no. 1 (2021): 3718040.

[149] H. Xiao , W. Cao , T. Ouyang , S. Guo , C. He , and J. Zhong , “Lattice Thermal Conductivity of Borophene from First Principle Calculation,” Scientific Reports 7, no. 1 (2017): 45986, 10.1038/srep45986. 28374853 PMC5379675

[150] J. Chen , Z. Wang , J. Ma , Z. Cao , K. Li , and J. Zhang , “Thermal and Electronic Properties of Borophene in Two‐dimensional Lateral Graphene‐borophene Heterostructures Empowered by Machine‐learning Approach,” Carbon 229 (2024): 119533.

[151] Y. Hu , Y. Yin , S. Li , H. Zhou , D. Li , and G. Zhang , “Three‐Fold Enhancement of in‐Plane Thermal Conductivity of Borophene through Metallic Atom Intercalation,” Nano Letters 20, no. 10 (2020): 7619–7626, 10.1021/acs.nanolett.0c03135. 32852213

[152] S. M. Mozvashi , M. A. Mohebpour , S. I. Vishkayi , and M. B. Tagani , “Mechanical Strength and Flexibility in $$∖Alpha '$$‐4H Borophene,” Scientific Reports 11, no. 1 (2021): 7547. 33824388 10.1038/s41598-021-87246-3 PMC8024380

[153] M. Faghihnasiri , H. Jafari , A. Ramazani , M. Shabani , S. M. Estalaki , and R. G. Larson , “Nonlinear Elastic Behavior and Anisotropic Electronic Properties of Two‐dimensional Borophene,” Journal of Applied Physics 125, no. 14 (2019): 145107.

[154] C. Zhong , X. Li , and P. Yu , “Strain‐tunable Dirac Semimetal Phase Transition and Emergent Superconductivity in a Borophane,” Communications Physics 7, no. 1 (2024): 38, 10.1038/s42005-024-01523-x.

[155] V.‐T. Pham and T.‐H. Fang , “Understanding Porosity and Temperature Induced Variabilities in Interface, Mechanical Characteristics and Thermal Conductivity of Borophene Membranes,” Scientific Reports 11, no. 1 (2021): 12123, 10.1038/s41598-021-91705-2. 34108570 PMC8190318

[156] S. M. Mozvashi , M. R. Givi , and M. B. Tagani , “The Effects of Substrate and Stacking in Bilayer Borophene,” Scientific Reports 12, no. 1 (2022): 13661, 10.1038/s41598-022-18076-0. 35953694 PMC9372144

[157] V. V. Kulish , “Surface Reactivity and Vacancy Defects in Single‐layer Borophene Polymorphs,” Physical Chemistry Chemical Physics 19, no. 18 (2017): 11273–11281, 10.1039/C7CP00637C. 28417128

[158] X. Yu , H. Cheng , M. Zhang , Y. Zhao , L. Qu , and G. Shi , “Graphene‐based Smart Materials,” Nature Reviews Materials 2, no. 9 (2017): 17046, 10.1038/natrevmats.2017.46.

[159] X. Fan , C. He , J. Ding , et al., “Graphene MEMS and NEMS,” Microsystems & Nanoengineering 10, no. 1 (2024): 154. 39468030 10.1038/s41378-024-00791-5 PMC11519522

[160] A. Carvalho , M. Wang , X. Zhu , A. S. Rodin , H. Su , and A. H. Castro Neto , “Phosphorene: from Theory to Applications,” Nature Reviews Materials 1, no. 11 (2016): 16061, 10.1038/natrevmats.2016.61.

[161] S. K. Sahoo and K.‐H. Wei , “A Perspective on Recent Advances in 2D Stanene Nanosheets,” Advanced Materials Interfaces 6, no. 18 (2019): 1900752.

[162] J.‐K. Lyu , S.‐F. Zhang , C.‐W. Zhang , and P.‐J. Wang , “Stanene: a Promising Material for New Electronic and Spintronic Applications,” Annalen der Physik 531, no. 10 (2019): 1900017.

[163] N. Liu , G. Bo , Y. Liu , X. Xu , Y. Du , and S. X. Dou , “Recent Progress on Germanene and Functionalized Germanene: Preparation, Characterizations, Applications, and Challenges,” Small 15, no. 32 (2019): 1805147. 10.1002/smll.201805147 30756479

[164] G. W. Mudd , S. A. Svatek , T. Ren , et al., “Tuning the Bandgap of Exfoliated InSe Nanosheets by Quantum Confinement,” Advanced Materials 25, no. 40 (2013): 5714–5718, 10.1002/adma.201302616. 23966225 PMC4065344

[165] D. A. Bandurin , A. V. Tyurnina , G. L. Yu , et al., “High Electron Mobility, Quantum Hall Effect and Anomalous Optical Response in Atomically Thin InSe,” Nature Nanotechnology 12, no. 3 (2017): 223–227, 10.1038/nnano.2016.242. 27870843

[166] S. Tongay , H. Sahin , C. Ko , et al., “Monolayer Behaviour in Bulk ReS_2_ due to Electronic and Vibrational Decoupling,” Nature Communications 5, no. 1 (2014): 3252, 10.1038/ncomms4252. 24500082

[167] C. Hou , G. Tai , Y. Liu , Z. Wu , X. Liang , and X. Liu , “Borophene‐based Materials for Energy, Sensors and Information Storage Applications,” Nano Research Energy 2 (2023): 9120051, 10.26599/NRE.2023.9120051.

[168] S.‐W. Chen , S.‐M. Huang , H.‐S. Wu , et al., “A Facile, Fabric Compatible, and Flexible Borophene Nanocomposites for Self‐Powered Smart Assistive and Wound Healing Applications,” Advanced Science 9, no. 22 (2022): 2201507, 10.1002/advs.202201507. 35657078 PMC9353498

[169] N. K. Das , S. Chahal , and S. Badhulika , “Highly Electronegative Borophene/PVDF Composite Hybrid Nanofibers Based Triboelectric Nanogenerator for Self‐powered Sensor for human Motion Monitoring and Energy Harvesting from Rain,” Materials Science in Semiconductor Processing 180 (2024): 108555, 10.1016/j.mssp.2024.108555.

[170] R. Kaimal , M. C. Maridevaru , A. Dube , J. J. Wu , A. Sambandam , and M. Ashokkumar , “Borophene Nanosheet‐Based Electrochemical Sensing toward Groundwater Arsenic Detection,” Industrial & Engineering Chemistry Research 62, no. 38 (2023): 15418–15427.

[171] S. M. Hosseini , A. Imanpour , M. Rezavand , et al., “Electrochemoresistance Sensor: a Borophene‐Based Sensor with Simultaneous Electrochemical and Chemoresistance Sensing Capability,” ACS Materials Letters 6, no. 3 (2024): 933–942, 10.1021/acsmaterialslett.3c01120.

[172] C. Hou , G. Tai , Y. Liu , et al., “Borophene Pressure Sensing for Electronic Skin and human‐machine Interface,” Nano Energy 97 (2022): 107189, 10.1016/j.nanoen.2022.107189.

[173] G. Baytemir , İ. Gürol , S. Karakuş , C. Taşaltın , and N. Taşaltın , “Nickel Phthalocyanine‐borophene Nanocomposite‐based Electrodes for Non‐enzymatic Electrochemical Detection of Glucose,” Journal of Materials Science: Materials in Electronics 33, no. 20 (2022): 16586–16596.

[174] C. Taşaltın , “Glucose Sensing Performance of PAN: Β‐rhombohedral Borophene Based Non‐enzymatic Electrochemical Biosensor,” Inorganic Chemistry Communications 133 (2021): 108973.

[175] C. Taşaltın , T. A. Türkmen , N. Taşaltın , and S. Karakuş , “Highly Sensitive Non‐enzymatic Electrochemical Glucose Biosensor Based on PANI: Β12 Borophene,” Journal of Materials Science: Materials in Electronics 32, no. 8 (2021): 10750–10760.

[176] S. Güngör , C. Taşaltın , İ. Gürol , G. Baytemir , S. Karakuş , and N. Taşaltın , “Copper Phthalocyanine‐borophene Nanocomposite‐based Non‐enzymatic Electrochemical Urea Biosensor,” Applied Physics A 128, no. 1 (2022): 89.

[177] G. Baytemir , “A Non‐enzymatic Electrochemical Sensor Based on Polyaniline/Borophene Nanocomposites for Dopamine Detection,” Applied Physics A 129, no. 2 (2023): 85, 10.1007/s00339-022-06364-5.

[178] Z. Wu , X. Liang , Z. Zhao , et al., “Ultrasensitive and Durable Borophene‐based Humidity Sensors for Advanced human‐centric Applications,” Chemical Engineering Journal 500 (2024): 156881, 10.1016/j.cej.2024.156881.

[179] Y. Kumar , S. Shankar , R. Chandra , and S. Kumar , “Highly Bendable and Smoke Free Degradable Nanomaterials Modified Paper Based Electrochemical Biosensor for Efficient Detection of Protein Biomarker,” Microchemical Journal 194 (2023): 109318, 10.1016/j.microc.2023.109318.

[180] E. P. Randviir , D. A. C. Brownson , and C. E. Banks , “A Decade of Graphene Research: Production, Applications and Outlook,” Materials Today 17, no. 9 (2014): 426–432, 10.1016/j.mattod.2014.06.001.

[181] M. Sup Choi , G.‐H. Lee , Y.‐J. Yu , et al., “Controlled Charge Trapping by Molybdenum Disulphide and Graphene in Ultrathin Heterostructured Memory Devices,” Nature Communications 4, no. 1 (2013): 1624, 10.1038/ncomms2652. 23535645

[182] S. S. T. Nibhanupudi , A. Roy , D. Veksler , M. Coupin , K. C. Matthews , and M. Disiena , “Ultra‐fast Switching Memristors Based on Two‐dimensional Materials,” Nature Communications 15, no. 1 (2024): 2334. 10.1038/s41467-024-46372-y PMC10940724 38485722

[183] L. Li , Y. Yu , G. J. Ye , et al., “Black Phosphorus Field‐effect Transistors,” Nature Nanotechnology 9, no. 5 (2014): 372–377, 10.1038/nnano.2014.35. 24584274

[184] Y. Zhou , D. Wu , Y. Zhu , et al., “Out‐of‐Plane Piezoelectricity and Ferroelectricity in Layered α‐In2Se3 Nanoflakes,” Nano Letters 17, no. 9 (2017): 5508–5513, 10.1021/acs.nanolett.7b02198. 28841328

[185] H. Fang , S. Chuang , T. C. Chang , K. Takei , T. Takahashi , and A. Javey , “High‐Performance Single Layered WSe_2_ p‐FETs with Chemically Doped Contacts,” Nano Letters 12, no. 7 (2012): 3788–3792, 10.1021/nl301702r. 22697053

[186] M. Zhou , W. Ming , Z. Liu , Z. Wang , P. Li , and F. Liu , “Epitaxial Growth of Large‐gap Quantum Spin Hall Insulator on Semiconductor Surface,” Proceedings of the National Academy of Sciences 111, no. 40 (2014): 14378–14381, 10.1073/pnas.1409701111. PMC4210051 25246584

[187] P. Vogt , P. De Padova , C. Quaresima , et al., “Silicene: Compelling Experimental Evidence for Graphenelike Two‐Dimensional Silicon,” Physical Review Letters 108, no. 15 (2012): 155501, 10.1103/PhysRevLett.108.155501. 22587265

[188] C. Hou , G. Tai , J. Hao , L. Sheng , B. Liu , and Z. Wu , “Ultrastable Crystalline Semiconducting Hydrogenated Borophene,” Angewandte Chemie International Edition 59, no. 27 (2020): 10819–10825, 10.1002/anie.202001045. 32243024

[189] R. Liu , C. Hou , X. Liang , Z. Wu , and G. Tai , “Borophene‐ZnO Heterostructures: Preparation and Application as Broadband Photonic Nonvolatile Memory,” Nano Research 16, no. 4 (2023): 5826–5833, 10.1007/s12274-022-5185-6.

[190] X. Liang , J. Hao , P. Zhang , C. Hou , and G. Tai , “Freestanding α‐rhombohedral Borophene Nanosheets: Preparation and Memory Device Application,” Nanotechnology 33, no. 50 (2022): 505601, 10.1088/1361-6528/ac8f9a. 36067735

[191] J. Liu , Z. Zeng , X. Cao , et al., “Preparation of MoS_2_ ‐Polyvinylpyrrolidone Nanocomposites for Flexible Nonvolatile Rewritable Memory Devices with Reduced Graphene Oxide Electrodes,” Small 8, no. 22 (2012): 3517–3522, 10.1002/smll.201200999. 22887650

[192] C. Tan , Z. Liu , W. Huang , and H. Zhang , “Non‐volatile Resistive Memory Devices Based on Solution‐processed Ultrathin Two‐dimensional Nanomaterials,” Chemical Society Reviews 44, no. 9 (2015): 2615–2628, 10.1039/C4CS00399C. 25877687

[193] A. Sebastian , M. Le Gallo , R. Khaddam‐Aljameh , and E. Eleftheriou , “Memory Devices and Applications for in‐memory Computing,” Nature Nanotechnology 15, no. 7 (2020): 529–544, 10.1038/s41565-020-0655-z. 32231270

[194] E. Yoo , J. Kim , E. Hosono , H. Zhou , T. Kudo , and I. Honma , “Large Reversible Li Storage of Graphene Nanosheet Families for Use in Rechargeable Lithium Ion Batteries,” Nano Letters 8, no. 8 (2008): 2277–2282, 10.1021/nl800957b. 18651781

[195] J. Hassoun , F. Bonaccorso , M. Agostini , et al., “An Advanced Lithium‐Ion Battery Based on a Graphene Anode and a Lithium Iron Phosphate Cathode,” Nano Letters 14, no. 8 (2014): 4901–4906, 10.1021/nl502429m. 25026051

[196] B. Anasori , Y. Xie , M. Beidaghi , et al., “Two‐Dimensional, Ordered, Double Transition Metals Carbides (MXenes),” ACS Nano 9, no. 10 (2015): 9507–9516, 10.1021/acsnano.5b03591. 26208121

[197] K. Chang and W. Chen , “l‐Cysteine‐Assisted Synthesis of Layered MoS_2_/Graphene Composites with Excellent Electrochemical Performances for Lithium Ion Batteries,” ACS Nano 5, no. 6 (2011): 4720–4728, 10.1021/nn200659w. 21574610

[198] J. Sun , G. Zheng , H.‐W. Lee , et al., “Formation of Stable Phosphorus–Carbon Bond for Enhanced Performance in Black Phosphorus Nanoparticle–Graphite Composite Battery Anodes,” Nano Letters 14, no. 8 (2014): 4573–4580, 10.1021/nl501617j. 25019417

[199] J. Sun , H.‐W. Lee , M. Pasta , et al., “A Phosphorene–graphene Hybrid Material as a High‐capacity Anode for Sodium‐ion Batteries,” Nature Nanotechnology 10, no. 11 (2015): 980–985, 10.1038/nnano.2015.194. 26344183

[200] X. Zhang , J. Hu , Y. Cheng , H. Y. Yang , Y. Yao , and S. A. Yang , “Borophene as an Extremely High Capacity Electrode Material for Li‐ion and Na‐ion Batteries,” Nanoscale 8, no. 33 (2016): 15340–15347, 10.1039/C6NR04186H. 27502997

[201] Y. Zhang , Z.‐F. Wu , P.‐F. Gao , S.‐L. Zhang , and Y.‐H. Wen , “Could Borophene Be Used as a Promising Anode Material for High‐Performance Lithium Ion Battery?,” ACS Applied Materials & Interfaces 8, no. 34 (2016): 22175–22181, 10.1021/acsami.6b05747. 27487298

[202] S. T.E. , D. T. Tran , S. Jena , et al., “Flexible 2D Borophene‐stacked MXene Heterostructure for High‐performance Supercapacitors,” Chemical Engineering Journal 481 (2024): 148266, 10.1016/j.cej.2023.148266.

[203] B. Murugesan , D. kumar Chinnalagu , A. Rajaiah , et al., “Integration of 2D Borophene into Semiconducting N, P, S, and F‐doped 1D Carbon for Energy Storage and Conversion Devices,” Chemical Engineering Journal 499 (2024): 156619, 10.1016/j.cej.2024.156619.

[204] W. Shao , Z. Wu , Y. Liu , and G. Tai , “Stacked Borophene‐based Electric Double‐layer Supercapacitors,” Chemical Engineering Journal 500 (2024): 157258, 10.1016/j.cej.2024.157258.

[205] O. P. Nanda , C. Y. Kong , and S. Badhulika , “Rapid Microwave‐Assisted Synthesis of a 2D Borophene‐Graphene Composite Embedded in a 3D Porous Hydrogel for Flexible Solid‐State Supercapacitors with High Energy Density,” ACS Applied Energy Materials 7, no. 18 (2024): 7844–7853, 10.1021/acsaem.4c01437.

[206] H. Lin , H. Shi , Z. Wang , et al., “Scalable Production of Freestanding Few‐Layer β12‐Borophene Single Crystalline Sheets as Efficient Electrocatalysts for Lithium–Sulfur Batteries,” ACS Nano 15, no. 11 (2021): 17327–17336, 10.1021/acsnano.1c04961. 34549941

[207] J. Ding , H. Zheng , S. Wang , and X. Ji , “Hydrogenated Borophene Nanosheets Based Multifunctional Quasi‐solid‐state Electrolytes for Lithium Metal Batteries,” Journal of Colloid and Interface Science 615 (2022): 79–86, 10.1016/j.jcis.2022.01.163. 35124508

[208] Y. Jiao , Y. Zheng , M. Jaroniec , and S. Z. Qiao , “Design of Electrocatalysts for Oxygen‐ and Hydrogen‐involving Energy Conversion Reactions,” Chemical Society Reviews 44, no. 8 (2015): 2060–2086, 10.1039/C4CS00470A. 25672249

[209] J. Xu , G. Shao , X. Tang , et al., “Frenkel‐defected Monolayer MoS_2_ Catalysts for Efficient Hydrogen Evolution,” Nature Communications 13, no. 1 (2022): 2193, 10.1038/s41467-022-29929-7. PMC9033855 35459263

[210] Y. Lu , B. Li , N. Xu , et al., “One‐atom‐thick Hexagonal Boron Nitride co‐catalyst for Enhanced Oxygen Evolution Reactions,” Nature Communications 14, no. 1 (2023): 6965, 10.1038/s41467-023-42696-3. PMC10618520 37907502

[211] Y. Gu , N. Nie , J. Liu , et al., “Enriching H_2_O through Boron Nitride as a Support to Promote Hydrogen Evolution from Non‐Filtered Seawater,” EcoEnergy 1, no. 2 (2023): 405–413, 10.1002/ece2.9.

[212] Y. Chang , P. Zhai , J. Hou , J. Zhao , and J. Gao , “Excellent HER and OER Catalyzing Performance of Se‐Vacancies in Defects‐Engineered PtSe_2_: from Simulation to Experiment,” Advanced Energy Materials 12, no. 1 (2022): 2102359, 10.1002/aenm.202102359.

[213] Y. Zuo , N. Antonatos , L. Děkanovský , et al., “Defect Engineering in Two‐Dimensional Layered PdTe_2_ for Enhanced Hydrogen Evolution Reaction,” ACS Catalysis 13, no. 4 (2023): 2601–2609, 10.1021/acscatal.2c04968.

[214] B. Pratihar , A. Jana , A. Kumar De , and S. De , “2D Boron Nanosheets for Photo‐ and Electrocatalytic Applications,” Chemcatchem 16, no. 12 (2024): 202301527, 10.1002/cctc.202301527.

[215] S. Ajmal , J. Huang , M. Singh , et al., “Realizing Electrochemical Energy Conversion and Storage with Borophene: Science, Engineering, Obstacles, and Future Opportunities,” Small 21, no. 10 (2025): 2411311. 10.1002/smll.202411311 39930846

[216] K. Chen , Z. Wang , L. Wang , et al., “Boron Nanosheet‐Supported Rh Catalysts for Hydrogen Evolution: a New Territory for the Strong Metal‐Support Interaction Effect,” Nano‐Micro Letters 13, no. 1 (2021): 138, 10.1007/s40820-021-00662-y. 34138393 PMC8187687

[217] G. Tai , M. Xu , C. Hou , R. Liu , X. Liang , and Z. Wu , “Borophene Nanosheets as High‐Efficiency Catalysts for the Hydrogen Evolution Reaction,” ACS Applied Materials & Interfaces 13, no. 51 (2021): 60987–60994, 10.1021/acsami.1c15953. 34918510

[218] K. Wenelska , A. Dymerska , and E. Mijowska , “Promotion of Borophene/NiO‐based Electrocatalyst for Oxygen Evolution Reaction,” Chemical Engineering Journal 476 (2023): 146714, 10.1016/j.cej.2023.146714.

[219] A. Saad , D. Liu , Y. Wu , et al., “Ag Nanoparticles Modified Crumpled Borophene Supported Co_3_O_4_ Catalyst Showing Superior Oxygen Evolution Reaction (OER) Performance,” Applied Catalysis B: Environmental 298 (2021): 120529, 10.1016/j.apcatb.2021.120529.

[220] W. Mabhulusa , K. E. Sekhosana , and X. Fuku , “PdNiONF−Borophene Nanocomposite as a Promising Catalyst for Ethanol Electro‐Oxidation Reaction,” ChemElectroChem 11, no. 12 (2024): 202400138, 10.1002/celc.202400138.

[221] S. Gao , Y. Zhang , J. Bi , et al., “2D hydrogenated Boride as a Reductant and Stabilizer for in Situ Synthesis of Ultrafine and Surfactant‐free Carbon Supported Noble Metal Electrocatalysts with Enhanced Activity and Stability,” Journal of Materials Chemistry A 8, no. 36 (2020): 18856–18862, 10.1039/D0TA06542K.

[222] M. Ou , X. Wang , L. Yu , et al., “The Emergence and Evolution of Borophene,” Advanced Science 8, no. 12 (2021): 2001801, 10.1002/advs.202001801. 34194924 PMC8224432

[223] P. Kumar , “Electric‐field Mediated Nickel‐induced Nanocrystallization of Amorphous Silicon Thin Films in the Complete Absence of External Heating,” Applied Physics A 98, no. 2 (2010): 473–479, 10.1007/s00339-009-5430-9.

[224] P. Kumar , “Magnetic Behavior of Surface Nanostructured 50‐nm Nickel Thin Films,” Nanoscale Research Letters 5, no. 10 (2010): 1596–1602, 10.1007/s11671-010-9682-2. 21076670 PMC2956046

[225] P. Kumar , Q. Nian , G. Xiong , T. S. Fisher , and G. J. Cheng , “Laser Engineering of Heterostructured Graphitic Petals on Carbon Nanotube Forests for Robust Thermal Interface Capable of Swift Heat Transfer,” Materials Advances 4, no. 11 (2023): 2402–2409, 10.1039/D3MA00134B.

[226] L. Li , J. F. Schultz , S. Mahapatra , Z. Lu , X. Zhang , and N. Jiang , “Chemically Identifying Single Adatoms with Single‐bond Sensitivity during Oxidation Reactions of Borophene,” Nature Communications 13, no. 1 (2022): 1796, 10.1038/s41467-022-29445-8. PMC8979967 35379784

[227] Y. Mu and S.‐D. Li , “First‐Principles Study on the Oxidation of Supported β12‐Borophene,” The Journal of Physical Chemistry C 124, no. 51 (2020): 28145–28151, 10.1021/acs.jpcc.0c09297.

[228] C. Martella , G. Faraone , M. H. Alam , et al., “Disassembling Silicene from Native Substrate and Transferring onto an Arbitrary Target Substrate,” Advanced Functional Materials 30, no. 42 (2020): 2004546, 10.1002/adfm.202004546.

[229] J. D. Wood , S. A. Wells , D. Jariwala , et al., “Effective Passivation of Exfoliated Black Phosphorus Transistors against Ambient Degradation,” Nano Letters 14, no. 12 (2014): 6964–6970, 10.1021/nl5032293. 25380142

[230] M. M. Morey , R. Bahadur , Z. Li , et al., “Experimental Realization of Fluoroborophene,” Small (2025): 2407763. 10.1002/smll.202407763 39479754

[231] R. Jain , Y. Singh , S.‐Y. Cho , et al., “Ambient Stabilization of Few Layer Phosphorene via Noncovalent Functionalization with Surfactants: Systematic 2D NMR Characterization in Aqueous Dispersion,” Chemistry of Materials 31, no. 8 (2019): 2786–2794, 10.1021/acs.chemmater.8b04984.

[232] V. Wang and W. T. Geng , “Lattice Defects and the Mechanical Anisotropy of Borophene,” The Journal of Physical Chemistry C 121, no. 18 (2017): 10224–10232, 10.1021/acs.jpcc.7b02582.

[233] N. Honari , S. M. Tabatabaei , M. Pourfath , and M. Fathipour , “Semiconducting Phase and Anisotropic Properties in Borophene via Chemical Surface Functionalization,” The Journal of Physical Chemistry C 124, no. 10 (2020): 5807–5816, 10.1021/acs.jpcc.9b06614.

[234] L. Li , J. F. Schultz , S. Mahapatra , et al., “Atomic‐Scale Insights into the Interlayer Characteristics and Oxygen Reactivity of Bilayer Borophene,” Angewandte Chemie International Edition 62, no. 32 (2023): 202306590, 10.1002/anie.202306590. 37321970

[235] K. S. Novoselov , A. Mishchenko , A. Carvalho , and A. H. Castro Neto , “2D materials and van der Waals Heterostructures,” Science 353, no. 6298 (2016): aac9439. 27471306 10.1126/science.aac9439

[236] Z. Wu , C. Shifan , Z. Wu , et al., “Epitaxial Growth of Borophene on Graphene Surface towards Efficient and Broadband Photodetector,” Nano Research 17, no. 4 (2024): 3053–3060, 10.1007/s12274-023-6109-9.

[237] Q. Ruan , L. Wang , K. V. Bets , and B. I. Yakobson , “Step‐Edge Epitaxy for Borophene Growth on Insulators,” ACS Nano 15, no. 11 (2021): 18347–18353, 10.1021/acsnano.1c07589. 34766759

[238] Y. Liu , Y. Huang , and X. Duan , “Van der Waals Integration before and beyond Two‐Dimensional Materials,” Nature 567, no. 7748 (2019): 323–333, 10.1038/s41586-019-1013-x. 30894723

[239] S. H. Choi , S. J. Yun , Y. S. Won , et al., “Large‐scale Synthesis of Graphene and Other 2D Materials Towards Industrialization,” Nature Communications 13, no. 1 (2022): 1484, 10.1038/s41467-022-29182-y. PMC8933535 35304474

[240] M. Rajapakse , B. Karki , U. O. Abu , et al., “Intercalation as a Versatile Tool for Fabrication, Property Tuning, and Phase Transitions in 2D Materials,” npj 2D Materials and Applications 5, no. 1 (2021): 30.

[241] Q. Zhang , L. Mei , X. Cao , Y. Tang , and Z. Zeng , “Intercalation and Exfoliation Chemistries of Transition Metal Dichalcogenides,” Journal of Materials Chemistry A 8, no. 31 (2020): 15417–15444, 10.1039/D0TA03727C.

[242] Y. Zhao , Y. Su , Y. Guo , and C. Wu , “Intercalation‐assisted Exfoliation Strategy for Two‐dimensional Materials Preparation,” Chemical Research in Chinese Universities 36, no. 4 (2020): 518–524, 10.1007/s40242-020-0159-2.

[243] A. Raza , J. Z. Hassan , M. Ikram , et al., “Advances in Liquid‐Phase and Intercalation Exfoliations of Transition Metal Dichalcogenides to Produce 2D Framework,” Advanced Materials Interfaces 8, no. 14 (2021): 2002205, 10.1002/admi.202002205.

[244] Y. Wang , Y. Liu , J. Zhang , et al., “Cryo‐mediated Exfoliation and Fracturing of Layered Materials into 2D Quantum Dots,” Science Advances 3, no. 12 (2017): 1701500, 10.1126/sciadv.1701500. PMC5731999 29250597

[245] V. Patil , S. Ghosh , A. Basu , et al., “Pick‐up and Assembling of Chemically Sensitive van Der Waals Heterostructures Using Dry Cryogenic Exfoliation,” Scientific Reports 14, no. 1 (2024): 11097, 10.1038/s41598-024-58935-6. 38750043 PMC11096354

[246] J. Ouyang , L. Zhang , L. Li , et al., “Cryogenic Exfoliation of 2D Stanene Nanosheets for Cancer Theranostics,” Nano‐Micro Letters 13, no. 1 (2021): 90, 10.1007/s40820-021-00619-1. 34138343 PMC8006518

[247] C. Zhang , Y. Xu , P. Lu , et al., “Cryogenic Exfoliation of Non‐Layered Magnesium into Two‐Dimensional Crystals,” Angewandte Chemie International Edition 58, no. 26 (2019): 8814–8818, 10.1002/anie.201903485. 31038834

[248] S. Li , H. Gunda , K. G. Ray , et al., “Spontaneous Dynamical Disordering of Borophenes in MgB_2_ and Related Metal Borides,” Nature Communications 12, no. 1 (2021): 6268, 10.1038/s41467-021-26512-4. PMC8560812 34725350

[249] F. Zhang , C. Jia , N. Zhang , et al., “Few‐layer Mg‐deficient Borophene Nanosheets: I2 Oxidation and Ultrasonic Delamination from MgB_2_ ,” Nanoscale 14, no. 11 (2022): 4195–4203, 10.1039/D1NR07353B. 35234763

[250] S. Yadav , M. A. Sadique , A. Kaushik , P. Ranjan , R. Khan , and A. K. Srivastava , “Borophene as an Emerging 2D Flatland for Biomedical Applications: Current Challenges and Future Prospects,” Journal of Materials Chemistry B 10, no. 8 (2022): 1146–1175, 10.1039/D1TB02277F. 35107476

[251] M. A. Sadique , S. Yadav , P. Ranjan , and R. Khan , “Potential Advancements and Upcoming Smart Research in Borophene: Challenges to Future,” in 2D Boron Nanosheets: Synthesis and Applications, ed. R. Khan , M. A. Sadique , S. Yadav , and A. Rotaru , (Springer Nature Singapore, 2024), 175–199.

[252] J.‐J. Zhang , T. Altalhi , J.‐H. Yang , and B. I. Yakobson , “Semiconducting α′‐boron Sheet with High Mobility and Low all‐boron Contact Resistance: a First‐principles Study,” Nanoscale 13, no. 18 (2021): 8474–8480, 10.1039/D1NR00329A. 33984112

[253] P. Sang , Q. Wang , W. Wei , Y. Li , and J. Chen , “Hydrogenated Borophene as a Promising Two‐Dimensional Semiconductor for Nanoscale Field‐Effect Transistors: a Computational Study,” ACS Applied Nano Materials 4, no. 11 (2021): 11931–11937, 10.1021/acsanm.1c02490.

[254] L.‐C. Xu , A. Du , and L. Kou , “Hydrogenated Borophene as a Stable Two‐dimensional Dirac Material with an Ultrahigh Fermi Velocity,” Physical Chemistry Chemical Physics 18, no. 39 (2016): 27284–27289, 10.1039/C6CP05405F. 27711580

[255] A. Vatankhahan and T. Movlarooy , “DFT Study of High‐Curie‐Temperature Ferromagnetism in α‐borophene Nanoribbons for Spintronic Applications,” Advanced Theory and Simulations 6, no. 11 (2023): 2200925.

[256] J. Yang , X. Liang , and Z. Yu , “Magnetoresistance and Spin‐dependent Seebeck Effects in Phthalocyanine‐based Molecular Junctions with Borophene Electrodes,” Physica E: Low‐dimensional Systems and Nanostructures 151 (2023): 115731.

[257] A. Vatankhahan and T. Movlarooy , “Modulating Spintronic Properties of Nitrogen Passivated Borophene Nanoribbons,” Materials Science and Engineering: B 281 (2022): 115744, 10.1016/j.mseb.2022.115744.

[258] F. Ghasemzadeh , M. Farokhnezhad , and M. Esmaeilzadeh , “Ultrafast Switching in Spin Field‐effect Transistors Based on Borophene Nanoribbons,” Physical Chemistry Chemical Physics 26, no. 17 (2024): 13061–13069, 10.1039/D4CP00239C. 38628071

[259] J. Ye , Z. Feng , H. Li , and X. Dai , “DFT+U Study on the Magnetic Properties of 3d Transition Metal Doped β12 Borophene,” Physica E: Low‐dimensional Systems and Nanostructures 147 (2023): 115576.

[260] F. I. M. Bidgoli , H. Nikoofard , N. Nikoofard , and M. Esmaeilzadeh , “Spin and Valley Filtering Properties in a Ferromagnetic 8‐pmmn Borophene Monolayer,” Journal of Physics and Chemistry of Solids 188 (2024): 111933, 10.1016/j.jpcs.2024.111933.

[261] C. Li , A. K. Tareen , K. Khan , et al., “Highly Efficient, Remarkable Sensor Activity and Energy Storage Properties of MXenes and Borophene Nanomaterials,” Progress in Solid State Chemistry 70 (2023): 100392, 10.1016/j.progsolidstchem.2023.100392.

[262] C. Hou , G. Tai , Y. Liu , and X. Liu , “Borophene Gas Sensor,” Nano Research 15, no. 3 (2022): 2537–2544, 10.1007/s12274-021-3926-6.

[263] L. Yan , R. Ku , J. Zou , et al., “Prediction of Superconductivity in Bilayer Borophenes,” RSC Advances 11, no. 63 (2021): 40220–40227, 10.1039/D1RA08014H. 35494119 PMC9044785

[264] C. Zhong , M. Sun , T. Altalhi , and B. I. Yakobson , “Superhard and Superconducting Bilayer Borophene,” Materials 17, no. 9 (2024): 1967, 10.3390/ma17091967. 38730773 PMC11084974

[265] J. Song , Y. Cao , J. Dong , and M. Sun , “Superior Thermoelectric Properties of Twist‐Angle Superlattice Borophene Induced by Interlayer Electrons Transport,” Small 19, no. 25 (2023): 2301348, 10.1002/smll.202301348. 36919623

[266] C. Ma , P. Yin , K. Khan , et al., “Broadband Nonlinear Photonics in Few‐Layer Borophene,” Small 17, no. 7 (2021): 2006891, 10.1002/smll.202006891. 33502109

[267] M. Cheng , P. Fu , and S. Chen , “Giant Photonic Spin Hall Effect in Bilayer Borophene Metasurfaces,” Optics Express 30, no. 22 (2022): 40075–40086, 10.1364/OE.473351. 36298946

[268] N. Ashraf and Y. Abghoui , “Borophene Potential for Developing Next‐Generation Battery Applications: a Comprehensive Review,” Energy & Fuels 37, no. 19 (2023): 14589–14603, 10.1021/acs.energyfuels.3c02319.

[269] X. Li , Y. Li , and Y. Wang , “Adsorption of Ca on Borophene for Potential Anode for Ca‐ion Batteries,” Journal of Molecular Modeling 29, no. 10 (2023): 308, 10.1007/s00894-023-05714-1. 37682404

[270] W. Shao , C. Hou , Z. Wu , P. Zhang , and G. Tai , “Stacking and Freestanding Borophene for Lithium‐ion Battery Application,” Nanotechnology 34, no. 31 (2023): 315401, 10.1088/1361-6528/acd121. 37116479

[271] J. Song , J. Wang , J. Yu , and M. Sun , “Electric, Optoelectronic, and Thermoelectric Properties of Moiré Superlattices of Bilayer Borophene with Different Twist Angles,” Advanced Electronic Materials 10, no. 5 (2024): 2300638, 10.1002/aelm.202300638.

[272] J. He , Y. Hu , D. Li , and J. Chen , “Ultra‐low Lattice Thermal Conductivity and Promising Thermoelectric Figure of Merit in Borophene via Chlorination,” Nano Research 15, no. 4 (2022): 3804–3811, 10.1007/s12274-021-3908-8.

[273] S. Ali , A. U. Rahman , and M. Sun , “Superior Optical and Thermoelectric Properties of Bilayer β12‐Like Phase Borophene Synthesized on Cu(111) Film,” Materials Advances 5, no. 7 (2024): 3029–3036, 10.1039/D3MA01121F.

[274] B. Mortazavi , M. Makaremi , M. Shahrokhi , et al., “Borophene Hydride: a Stiff 2D Material with High Thermal Conductivity and Attractive Optical and Electronic Properties,” Nanoscale 10, no. 8 (2018): 3759–3768, 10.1039/C7NR08725J. 29411815

[275] H. L. Stern , C. M. Gilardoni , Q. Gu , et al., “A Quantum Coherent Spin in Hexagonal Boron Nitride at Ambient Conditions,” Nature Materials 23, no. 10 (2024): 1379–1385, 10.1038/s41563-024-01887-z. 38769205 PMC11442369

[276] C. Li , Y.‐F. Zhao , A. Vera , et al., “Proximity‐induced Superconductivity in Epitaxial Topological Insulator/Graphene/Gallium Heterostructures,” Nature Materials 22, no. 5 (2023): 570–575, 10.1038/s41563-023-01478-4. 36781950

[277] Z. Xie , Y. Duo , T. Fan , et al., “Light‐induced Tumor Theranostics Based on Chemical‐exfoliated Borophene,” Light: Science & Applications 11, no. 1 (2022): 324, 10.1038/s41377-022-00980-9. PMC9652458 36369148

[278] S. Ahmed , A. Ansari , S. Kashif Ali , et al., “Pioneering Sensing Technologies Using Borophene‐Based Composite/Hybrid Electrochemical Biosensors for Health Monitoring: a Perspective,” Analysis & Sensing 4, no. 5 (2024): 202400034, 10.1002/anse.202400034.

[279] D. Gogoi , C. Hazarika , G. Neog , et al., “Borophene Quantum Dots as Novel Peroxidase‐Mimicking Nanozyme: a Dual‐Mode Assay for the Detection of Oxytetracycline and Tetracycline Antibiotics,” ACS Applied Materials & Interfaces 16, no. 12 (2024): 14645–14660. 38478795 10.1021/acsami.3c12108

[280] E. Czarniewska , K. Sielicki , K. Maślana , and E. Mijowska , “In Vivo Study on Borophene Nanoflakes Interaction with Tenebrio Molitor Beetle: Viability of Hemocytes and Short‐term Immunity Effect,” Scientific Reports 13, no. 1 (2023): 11823, 10.1038/s41598-023-38595-8. 37479709 PMC10361989

[281] A. K. Yagati , T. Wu , S. G. Chavan , M.‐K. Lee , M.‐H. Lee , and J. Min , “DNA Aptamers on Borophene Nanosheets as an Electrochemical System for Omicron Detection,” ACS Applied Nano Materials 6, no. 18 (2023): 17239–17250, 10.1021/acsanm.3c03669.

